# Ethnobotany of Anti-hypertensive Plants Used in Northern Pakistan

**DOI:** 10.3389/fphar.2018.00789

**Published:** 2018-07-24

**Authors:** Khafsa Malik, Mushtaq Ahmad, Rainer W. Bussmann, Akash Tariq, Riaz Ullah, Ali S. Alqahtani, Abdelaaty A. Shahat, Neelam Rashid, Muhammad Zafar, Shazia Sultana, Syed N. Shah

**Affiliations:** ^1^Department of Plant Sciences, Quaid-i-Azam University, Islamabad, Pakistan; ^2^Center for Natural Products Lab, Chengdu Institute of Biology, Sichuan, China; ^3^Department of Ethnobotany, Institute of Botany, Ilia State University, Tbilisi, Georgia; ^4^Key Laboratory of Mountain Ecological Restoration, Bioresource Utilization and Ecological Restoration Biodiversity Conservation Key Laboratory of Sichuan Province, Chengdu Institute of Biology, Chinese Academy of Sciences, Chengdu, China; ^5^University of Chinese Academy of Sciences, Beijing, China; ^6^Medicinal Aromatic and Poisonous Plants Research Center, College of Pharmacy King Saud University, Riyadh, Saudi Arabia; ^7^Phytochemistry Department, National Research Centre, Giza, Egypt

**Keywords:** hypertension, Northern Pakistan, medicinal plant, disease consensus index, ethnobotany

## Abstract

Hypertension is one of the most important factors responsible for cardiovascular ailments worldwide. It has been observed that herbal products and alternative herbal therapies played a significant role in decreasing hypertension. The aim of the current study is to provide significant ethnopharmacological information, both qualitative and quantitative on medicinal plants related to hypertension from Northern Pakistan. The documented data were quantitatively analyzed for the first time in this area. A total of 250 participants were interviewed through semi-structured discussions and questionnaires. Quantitative indices including FC (Frequency citation), FIV (Family importance value), RFC (Relative frequency of citation) and DCI (Disease Consensus index) were calculated. A total of 192 plant species, belonging to 77 families were reported to be used in treatment of hypertension in Northern Pakistan. The most dominant life form reported was herbs (54%), with decoction (72 reports) and leaves (55.1%) were commonly utilized plant part. Highest FIV was recorded in Lamiaceae (327 FIV). RFC ranged from 0.08 to 1.08% while DCI varied from 0.233 to 0.000. In this study original data was compared with thirty one previous national and international published papers from neighboring region to compare the medicinal uses and obtain some novel plant species. About 42% of the medicinal plant species were reported for the first time in treatment of hypertension in comparison to these 31 published papers. Different phytochemical activities of antihypertensive plants were also reported from literature. This research work documents the traditional knowledge of medicinal plants usage and provides baseline in designing clinical trials and pharmacological analysis for treatment of hypertension.

## Introduction

Hypertension is one of the most common cardiovascular diseases that become major health concern in various parts of the world. Arterial hypertension is a chronic medical condition in which the pressure in the arteries raised above 140/90 mmHg (Osamor and Owumi, 2010). There are two types of hypertension, systolic and diastolic hypertension. Based on detected blood pressure (BP) rates there are different groups of hypertension, mild or grade I (BP 140–159/90–99 mm Hg), moderate or grade II (BP 160–179/100–109 mm Hg), and severe or grade III and IV (BP>180–210/110–120 mm Hg) (Salud, 2001).

Family history, extensive use of alcohol, high sodium intake and high sugar intake might be one of the cause hypertension (Eddouks et al., 2002). Smoking and coffee consumption is also reported to be cause of hypertension. Ecological aspects such as lead polluted drinking water and cadmium contamination have also been shown to favor hypertension (Pirkle et al., 1985). The incidences of uncontrolled hypertension occur among individuals of above 50 years (Tee et al., 2010). Each year about half million strokes and more than a million heart attacks are caused due to hypertension (Jaffer and Weissleder, 2005; Sarafidis et al., 2008; Grassi et al., 2013). Many other conditions such as insulin resistance, obesity, kidney failure, nervous system, concomitance, atherosclerosis and cardiovascular diseases have been found to be related with high blood pressure (Ghosh and Bandyopadhyay, 2007; Heisler et al., 2008). Hypertension is estimated to cause 4.5% of the disease burden globally (World Health Organization, 2002; Cardoso and Salles, 2016).

Hypertension has become a worldwide concern with important scale of morbidity and mortality. It has been observed that 1 billion persons all over the World suffer from hypertension triggering up to 7.1 million casualties per year (Hajjar and Kotchen, 2003; Tahraoui et al., 2007). In Africa particularly in Gabon, it is reported (World Health Organization, 2001) that 26.4% of the inhabitants (26.1% female and 26.6% male) suffered from hypertension (Opie and Seedat, 2005). Similarly the prevalence of hypertension in the Asia-Pacific areas ranges from 7 to 38% in women and from 5 to 47% in men (Lawes et al., 2004). Around the globe, the lowest percentage of people with hypertensions (less than 5.2%) live in rural North India (Kearney et al., 2005).

In Pakistan a health survey (NHSP) between 1990 and 1994 emphasized on the extent of the burden of hypertension in Pakistan. Hypertension affected 18% of adults above 15 years and 33% of adults above 45 years, with 3% suffering from low hypertension at 140/90 mm Hg or below. In Pakistan it is reported that every third person over the above 40 years of ageis affected by hypertension (Saleem et al., 2010). In Ayurvedic and Greek traditional medicinal systems, hypertension was diagnosed by its apparent symptoms. In the current study local healers gave information about symptoms and signs of hypertension and indicated also which other sources of information about hypertension they might have had. The local healers spend much time listening to the patients suffering from any illness, and discussing the possible causes of the disease and the course of treatment with the patients. The perceived causes of hypertension by traditional healers include diet and hereditary causes, as found in other studies (Meli et al., 2009). The traditional healers also described other symptoms such as severe headache, fatigue, chest pain, irregular heart beat among others for a diagnosis of hypertension.

In homeopathy and traditional Chinese herbal medicinal system there are numerous treatments for hypertension (Gress et al., 2000). However, there is still a need for supplementary means for treatment, and also for non-pharmacological management (Gallagher et al., 2006). Such on-pharmacological methods include biofeedback, relaxation, weight reduce, drug treatment and dietary modifications, e.g., reduced salt intake, avoidance of excessive alcohol use and exercise (Farpour-Lambert et al., 2009), and non-smoking (Puddey and Beilin, 2006) for controlling mild hypertension.

The traditional use of plants as medicines started with the evolution of societies (Bandaranayake, 2006). Plants and animals have promising possibilities for drugs discovery (Newman and Cragg, 2007; Sahraie-Rad et al., 2015; Sharifi-Rad et al., 2017; Salehi et al., 2018a). Pure compounds might be helpful for treatment of various ailments (Bandaranayake, 2006; Sharifi-Rad et al., 2017, 2018; Salehi et al., 2018b). Alternative herbal medicines are often preferred over modern medicines (Nuwaha and Muganzi, 2008). According to the WHO about 80% of people rely on traditional medicines (Calixto, 2005). A recent study showed that 25% of modern drug and 75% of new medicines against virulent diseases are obtained from natural plant resources (Bedoya et al., 2009). Hypertension, has become one of the most common non-communicable diseases internationally and it is affecting up to 20% of the world's adult population (Osamor and Owumi, 2010). Educated people have more awareness about the side effects of allopathic drugs so they are more expected to take herbal extracts for treatment of hypertension (Gulla and Singer, 2000).

The aim of this study was to document indigenous knowledge related to herbal remedies for the treatment of hypertension by indigenous communities of Northern Pakistan. It is hoped that these results may help the conservation of traditional medicinal knowledge for future generations.

## Materials and methods

### Study area

**Figure 1:**
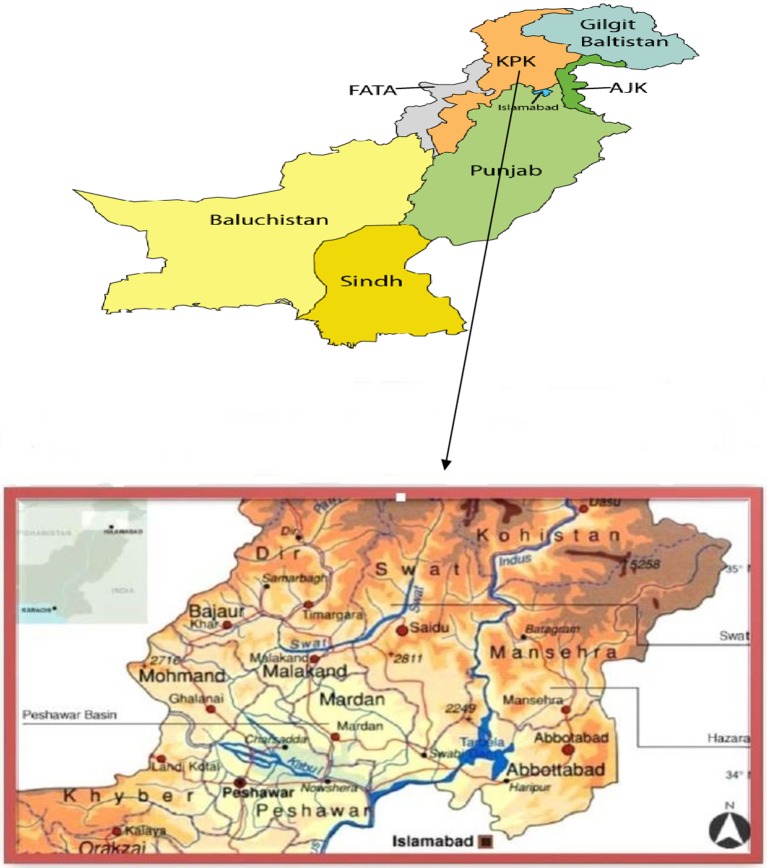
Map of the study area.

Northern Pakistan includes Swat valley, Hazara division, Manshera, Naran, Dir and other tribal areas (Figure 1). It shares a border with Gilgit Baltistan in the north east, FATA (Federal Administered Tribal Areas) in the West-South, Punjab in the South east, and Azad Jammu and Kashmir in the North. The northern regions of Pakistan are home of its largest mountain ranges, and these cover 72,496 km^2^. This region includes the foothills of Himalayas, Hindukush and Karakorum ranges (Hamayun, 2003), and harbor particular plant species used as edible plants, medicinal and aromatic plants (Ali and Qaiser, 1986). The temperature varies from 3.4°C to 34.3°C, with winter snowfall in hilly areas (Chevallier et al., 2011). The area has old cultural traditions, festival, dresses and ceremonies. The majority of people speak Pushto, other local languages are Potohari, Gujrati, and Hindko.

### Ethics approval and consent to participate

Before conducting interviews, the individual prior informed consent was obtained from all participants. No further ethics approval was required. All work conducted was carried out under the stipulations of the Nagoya Protocol on Access to Genetic Resources and the Fair and Equitable Sharing of Benefits Arising from their Utilization to the Convention on Biological Diversity. The right to use and authorship of any traditional knowledge of all participants is maintained, and any use of this information, other than for scientific publication, does require additional prior consent of the traditional owners, as well as a consensus on access to benefits resulting from subsequent use.

### Field survey and demographic data

Field surveys were conducted during July 2015 to March 2016 in order to document traditional knowledge in different areas of Northern Pakistan. During the field surveys, the indigenous uses of plants for the treatment of hypertension were recorded through interviews with experienced Traditional Health Practitioners (THPs) and local people. The objectives of the study were clearly explained, prior informed oral consent was obtained, and interviews were conducted in local languages to obtain the knowledge of medicinal plants. Semi-structured questionnaires were used (Martin, 1995). Data documentation and field surveys were assessed by using quantitative indices. Questionnaire (Table 1) was mainly focused on the ethnobotanical knowledge of native communities. In these interviews, the first part of the questionnaire was concerned with demographic information of the participants including gender, age, informant category, educational background and residence while second part contain informative questions about the local names of plant, identification, habit, mode of preparation, plant part used, medicinal use and method of administration. Research articles, relevant web pages and books were also studied with the aim to collect data of phytochemical compounds and toxicity present in the plants. Data documentation and field surveys were assessed by using quantitative ethnobotanical indices.

**Table 1:** Demographic profile of participants.

**S/No**	**Variables**	**Categories**	**No. of person**	**Percentages**
1.	Gender	Female	55	22
		Male	195	78
2.	Informant category	Local people	175	70
		Herbalist	75	30
3.	Age group	35–45	16	7
		45–55	72	28
		55–65	114	46
		Above than 65	48	19
4.	Educational background	Illiterate	78	31
		Completed 6 years of education	65	26
		Completed 10 years of education	46	18
		Completed 12 years of education	31	12
		16 years of education	20	8
		Higher education	10	4
5.	Resident	Urban	78	31
		Rural	172	69

In this, total of 250 traditional healers (175 local people and 75 herbalists) were interviewed. The local healers diagnosed hypertension by watching for the following symptoms: severe headache, fatigue, chest pain, vision problems, breathing problems, irregular heartbeat, blood in the urine, pounding in the chest, cardiovascular problems and dizziness. The majority of informant falls in the age category of 35–45 years (7%) followed by 45–55 years (28%), 55–65 years (46%) and above than 65 years of age (19%). The participants were divided into different categories on the basis of educational background i-e illiterate (31%), completed 6 years of education (26%), 10 years of education (18%), 12 years of education (12%), and 16 years of education (8%) while (4%) were of higher education (Table 1). Large number of herbal plants were used for treatment of diseases by illiterate villagers showing that in under developed areas people still depend on ethno medicinal plants. Other demographic data was collected with 31% of the participants living in urban and 69% in rural communities (Table 1).

### Plant collection and identification

The plants were collected from different areas of northern Pakistan in different seasons during the year. In present study medicinal plants reported by the local informants were identified by vernacular names and collected in the field. These specimens were later reconfirmed for correct identification using the services of senior Plant Taxonomist Professor Dr. Mir Ajab Khan (PhD in Plant Taxonomy), from department of Plant Sciences Quaid-i- Azam University, Islamabad. The collected plant specimens were dried and preserved by following standard herbarium techniques recommended by Jain and Rao (1977). The plant names were verified by using databases such as KEW medicinal plant name services (mpns: http://www.kew.org/mpns). Voucher specimens were deposited in the herbarium, of Department of Plant Sciences, Quaid-i-Azam University (ISL), Islamabad.

### Quantitative analysis

Results were analyzed using quantitative indices like Disease Consensus Index (DCI), Frequency of Citation (FC), Relative Frequency Citation (RFC) and Family Importance Value (FIV).

### Diseases consensus index (DCI)

It is used to evaluate the plant knowledge to cure specific ailment and the degree of consensus that how people recommend plant to treat specific disease. Diseases Consensus Index (DCI) is calculated by using formula followed by Andrade-Cetto et al. (2006).

$$\text{DCI}=\left(\frac{\sum \text{V}\text{xi}}{\text{CC}}\text{m}(\text{Vx})\;\right){\text{Pm}}^{-0.1}$$

Where∑(*Vxi*) = Total sum of the ideal uses for a species. Vx is calculated by using formula Vx = No of questions answered for a species/Total questions asked. The value of Vx ranges from 0 to 1. mVx represents statistical mean of total ideal values (Vxi); CC is correlation coefficient, Pm−0.1 is the compensation factor that analyzes the dispersion of indigenous knowledge considering the mode of preparation and parts used.

### Relative frequency of citation (RFC)

The RFC is calculated to determine the level of traditional knowledge about the use of medicinal plants in the study areas. Relative Frequency of Citation (RFC) was calculated by using the formula.

$$\begin{array}{l} RFC\;=\;Fc/N \end{array}$$

Where the number of informants who mentioned the use of the species is “Fc” and “N” is the total number of informants (Tardío and Pardo-De-Santayana, 2008).

### Family importance value (FIV)

FIV values show the knowledge of informant about the families of plant species used. FIV of the medicinal plant being calculated by using formula as under (Molares and Ladio, 2009).

$$\begin{array}{l} \text{FIV}=\text{No of families cited by authors }/\text{ total no of authors }\times 100 \end{array}$$

High FIV value reveals that there is plenty of knowledge and several authors are more known while less FIV values indicates that there is less awareness about the use of family.

### Jaccard index (JI)

Jaccard Index (JI) is calculated by comparing previously published research articles from local, regional and global level by calculating the percentages of cited plants species and their medicinal utilization by using the following formula:

$$\begin{array}{l} \text{JI}=\text{c multiply }100/\text{a}+\text{b}-\text{c} \end{array}$$

where “a” is the number of plants of the region A, “b” is the number of plants of the region B, and “c” is the number of plants common to A and B (Kayani et al., 2015) (Table 2).

**Table 2:** Comparison of present study with previous studies at neighboring, regional and global level.

**S. No**	**Study area**	**Year**	**Number of recorded plant spp. Of aligned areas**	**Plants with similar uses**	**Plants with dissimilar uses**	**Total spp. Common in both area**	**%age of plant spp. Common in both areas**	**Species enlisted only in aligned areas**	**Species enlisted only in study area**	**% of spp. Enlisted only in study area**	**% of plants with similar uses**	**% of dissimilar uses**	**Jaccard index (JI)**	**Citation**
1	UK	2001	3	0	0	0	0	3	196	100	0	0	0	Mansoor, 2001b
2	South Africa	2003	1	1	0	1	100	0	195	99.4898	0.510204	0	0.515464	Somova et al., 2003
3	Morocco	2001	90	1	23	24	26.66667	66	172	87.7551	0.510204	11.73469	11.21495	Jouad et al., 2001
4	Morocco	2002	92	43	0	43	46.73913	49	153	78.06122	21.93878	0	27.04403	Eddouks et al., 2002
5	Morocco	2006	64	36	0	36	56.25	28	160	81.63265	18.36735	0	23.68421	Tahraoui et al., 2007
6	Loja and Zamora-Chinchipe, Ecuador	2007	275	6	24	30	10.90909	245	166	84.69388	3.061224	12.2449	7.874016	Tene et al., 2007
7	Malaysia	2010	2	0	0	0	0	2	196	100	0	0	0	Tee et al., 2010
8	DIR Pakistan 2015	2015	46	12	0	12	26.08696	34	184	93.87755	6.122449	0	5.825243	Ahmad et al., 2015
9	Chennai	2011	10	5	0	5	50	5	191	97.44898	2.55102	0	2.617801	Roy, 2011
10	Ghana	2005	10	2	0	2	20	8	194	98.97959	1.020408	0	1	Abel and Busia, 2005
11	Nepal	2008	3	0	1	1	33.33333	2	195	99.4898	0	0.510204	0.510204	Kunwar and Bussmann, 2008
12	Nepal	2006	84	.0	2	2	2.380952	82	194	98.97959	0	1.020408	0.729927	Kunwar et al., 2006
13	Uttar Pardesh	2007	2	0	0	0	0	2	196	100	0	0	0	Verma et al., 2007
14	India	2011	57	0	2	2	3.508772	55	194	98.97959	0	1.020408	0.809717	Kumar et al., 2011
15	Nepal	2010	48	0	1	1	2.083333	47	195	99.4898	0	0.510204	0.414938	Kunwar et al., 2010
16	Swat, Pakistan	2013	54	0	15	15	27.77778	39	181	92.34694	0	7.653061	7.317073	Akhtar et al., 2013
17	Lesser Himalaya Pakistan	2013	45	0	3	3	6.666667	42	193	98.46939	0	1.530612	1.293103	Abbasi et al., 2013b
18	Swat Pakistan	2014	50	0	6	6	12	44	190	96.93878	0	3.061224	2.631579	Ahmad et al., 2014
19	Karakoram range Pakistan	2014	50	0	3	3	6	47	193	98.46939	0	1.530612	1.265823	Bano et al., 2014a
20	Pakistan	2014	26	0	3	3	11.53846	23	193	98.46939	0	1.530612	1.408451	Sher et al., 2014
21	South East Asia	2015	183	0	0	0	0	183	196	100	0	0	0	Hidayati et al., 2015
22	Bhutan	2015	5	0	0	0	0	5	196	100	0	0	0	Wangchuk and Tobgay, 2015
23	Afghanistan	2016	72	0	5	5	6.944444	67	191	97.44898	0	2.55102	1.976285	Soelberg and Jäger, 2016
24	China	2016	54	0	5	5	9.259259	49	191	97.44898	0	2.55102	2.12766	Kang et al., 2016
25	Iran	1999	1	1	0	1	100	0	195	99.4898	0.510204	0	0.515464	Faraji and Tarkhani, 1999
26	Cameroon	2002	1	1	0	1	100	0	195	99.4898	0.510204	0	0.515464	Dimo et al., 2002
27	Pakistan	2017	2	0	0	0	0	2	196	100	0	0	0	Saqib and Janbaz, 2016
28	Nigeria	2012	70	13	0	13	18.57143	57	183	93.36735	6.632653	0	5.726872	Gbolade, 2012
29	Chinese	2014	5	0	0	0	0	5	196	100	0	0	0	Tsai et al., 2014
30	Buner Pakistan	2011	216	0	22	22	10.18519	194	174	88.77551	0	11.22449	6.358382	Sher et al., 2011
31	Swat Pakistan	2011	90	0	9	9	10	81	187	95.40816	0	4.591837	3.474903	Ali et al., 2011
Average			55.19355	3.903226	4	7.903226	22.48069	47.29032	188.0968	95.96774	1.991442	2.040816	3.769405	

## Results

### Diversity of medicinal plants hypertension

**Figure 2:**
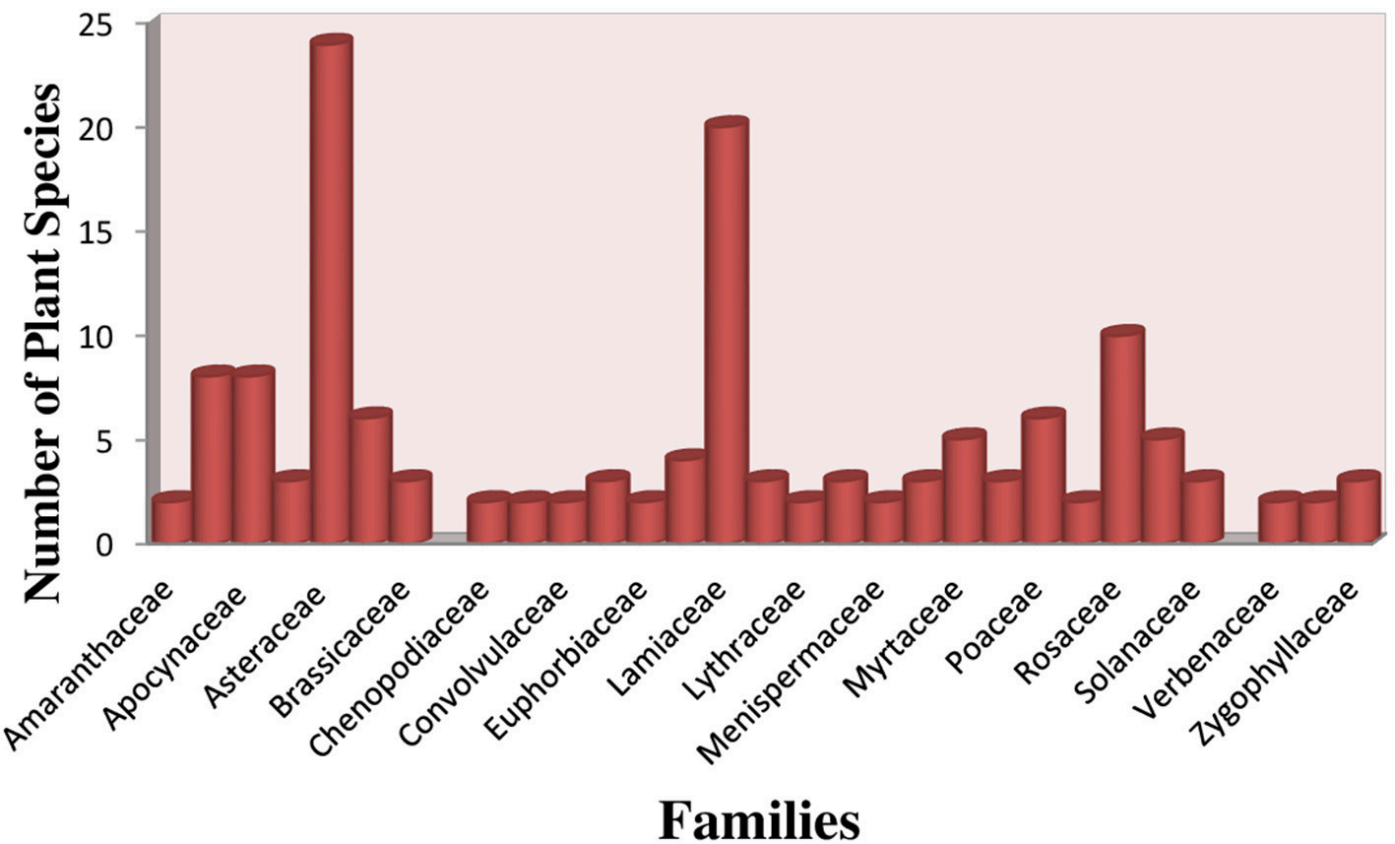
Dominant families of medicinal plants in the study area.

Total 192 plant species have been reported for the treatment of hypertension. Plant information with botanical, English, vernacular and family name, mode of utilization, habit, phytochemistry, toxicity, RFC, FIV, and DCI are given in Supplementary Table 1. In this study medicinal plants belonging to 77 plant families and 171 Genera were reported. Among these Asteraceae was observed to be predominant family (23 species), followed by Lamiaceae (19 species), Rosaceae (10 species), Apiaceae and Apocynaceae (8 species each), Boraginaceae and Poaceae (6 species each) Rutaceae (5 species). Fabaceae (4 species), Asclepiadaceae, Caesalpiniaceae, Brassicaceae, Cucurbitaceae, Moraceae, Zygophyllaceae and Solanaceae (3 species each), Amaranthaceae, Chenopodiaceae, Commelinaceae, Crassulaceae, Euphorbiaceae, Lythraceae, Malvaceae, Menispermaceae, Papaveraceae, Polygonaceae, Valerianaceae, Verbenaceae, Vitaceae (2 species each) and rest of families reported to have only (1 species each) used in hypertension treatment (Figure 2).

### Plant parts used and life forms

**Figure 3:**
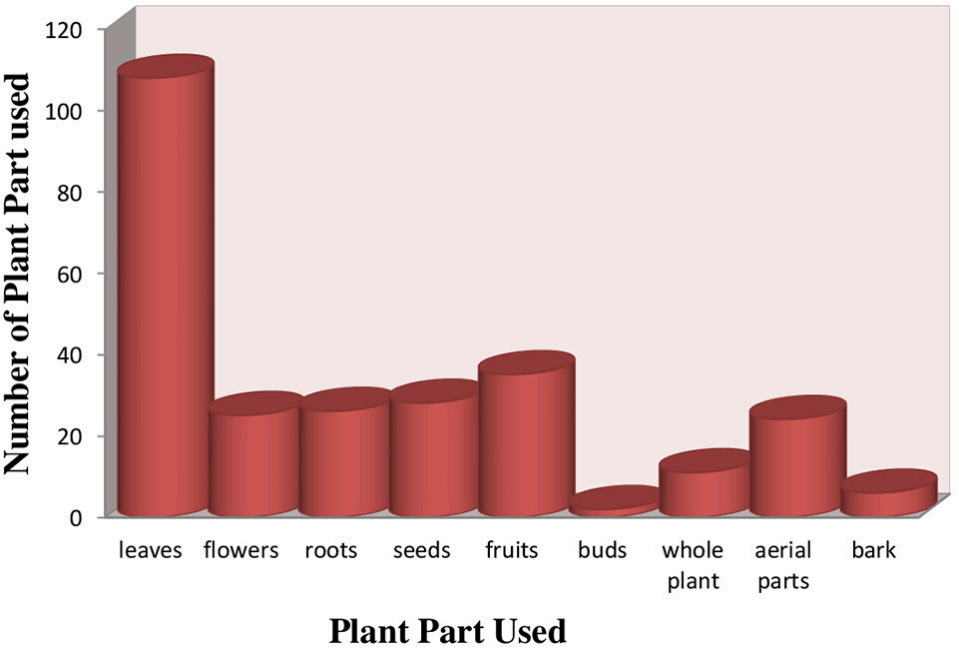
Plant part used for treatment of hypertension.

**Figure 4:**
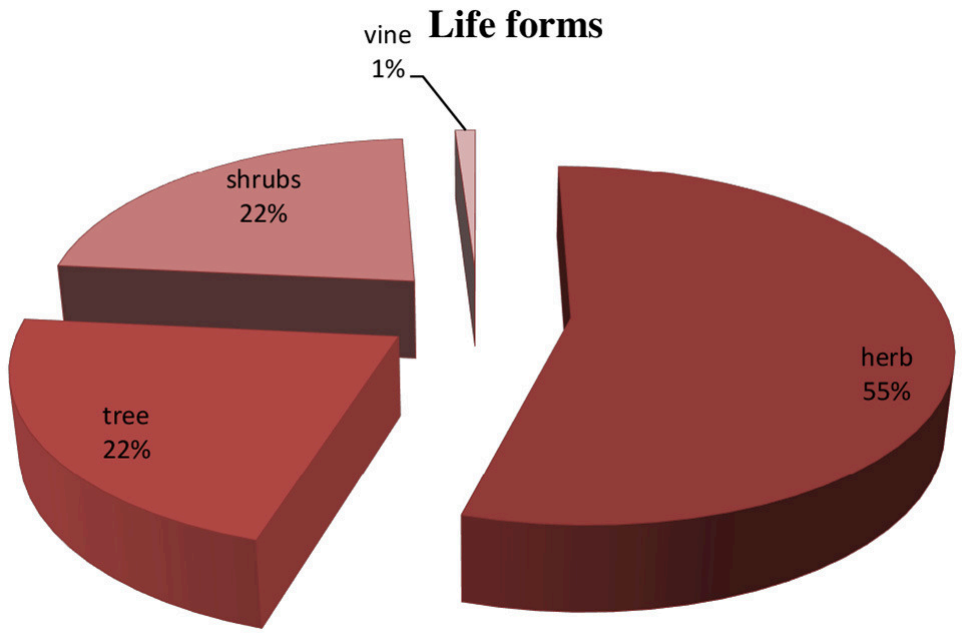
Life forms of medicinal plants.

Different plant part used in preparation of herbal recipes include root, stem, leaves, flower, seed, fruit, bark, aerial parts, and whole plant are also used when small herbal plants was practiced. Leaves were most frequently part used (55.1%), followed by fruit (17.8%), seed (14.2%), roots, aerial part and flower (12.7% each), whole plant (5.6%) and bark (3.0%) (Figure 3) (Supplementary Table 1). Essential source of native medicines were as herbs (54%), shrubs (23%), trees (22%) (Figure 4).

### Preparation of herbal medicines

**Figure 5:**
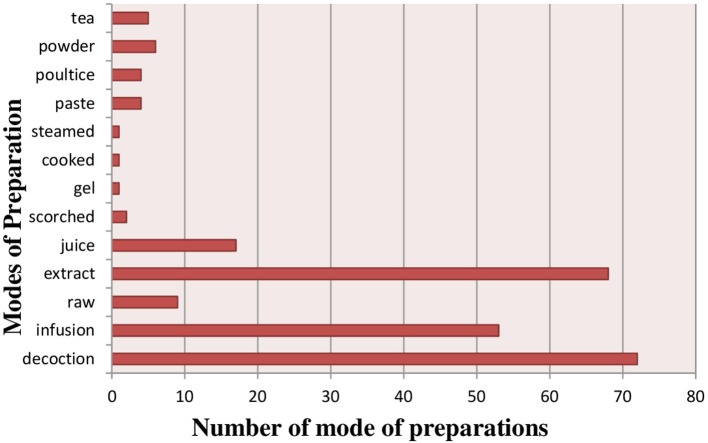
Mode of utilization of medicinal plants.

Herbal preparations were made by using different preparation modes i.e., infusion, powder, decoction, extract, juice, paste, tea, poultice, scorched, gel, cooked and steamed (Figure 5) (Supplementary Table 1). The most frequently reported mode of preparation was decoction (72 species), followed by extracts (68 species), infusions (53 species), juice (17 species) and Powder (7 species). Infusions were prepared by soaking a plant in water for more than an hour at room temperature, while decoctions were obtained from boiling the plant part in water until the water volume is reduced to half of its original volume. Juice was obtained by extracting and grinding the fresh part of plants and then mix in any liquid. Paste was made by grinding fresh plant material with water. Different dosage of herbal preparations was used by local communities for treatment of various ailments.

### Quantitative data analysis

#### FIV (family importance value)

**Figure 6:**
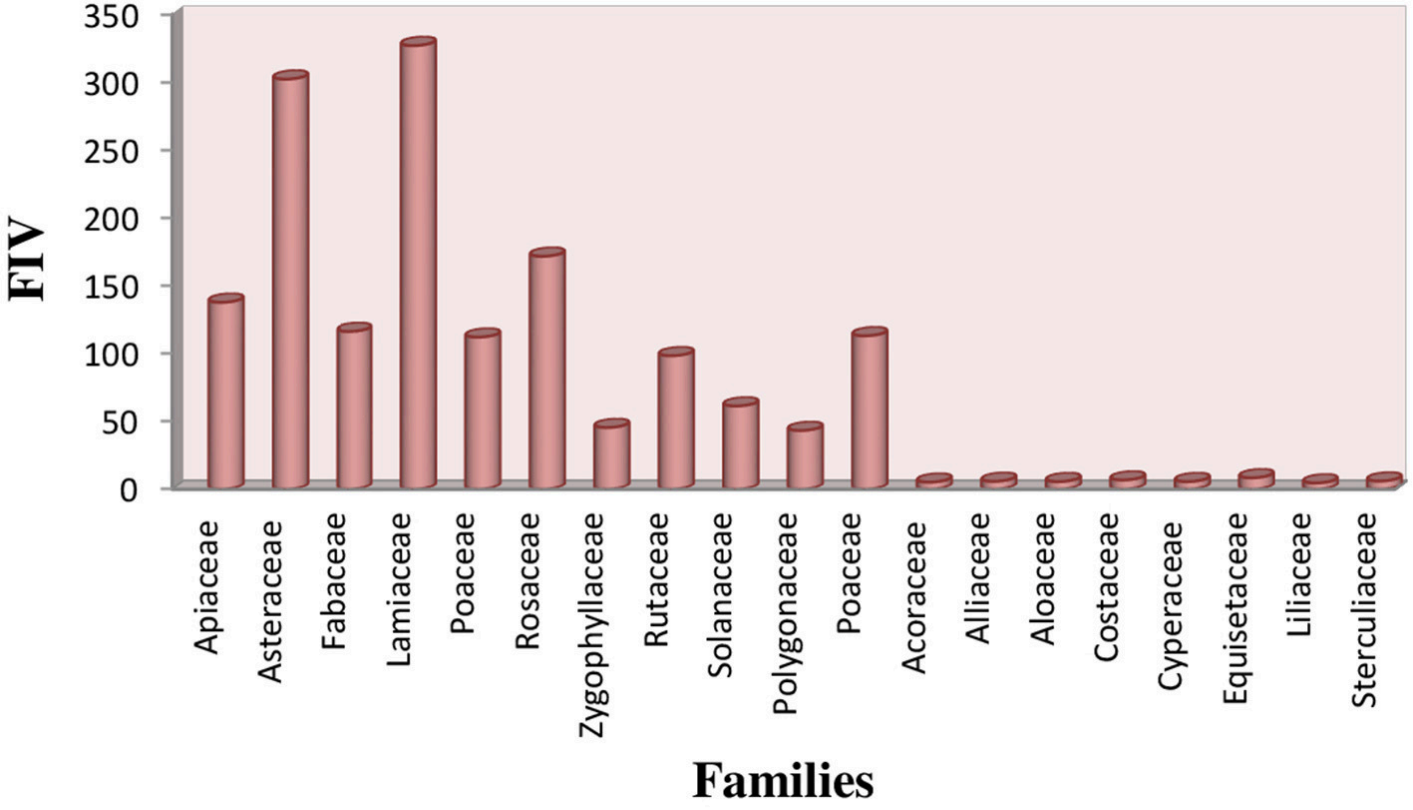
Family importance value of medicinal plants.

The most common families of indigenous herbal plant species as represented by its FIV were Lamiaceae (327 FIV) and Asteraceae (302 FIV), followed by Rosaceae (174 FIV), Apiaceae (138 FIV), Fabaceae (117 FIV). The least value of FIV was observed for Acoraceae, Alliaceae, Alocaceae and Cyperaceae (5 FIV) (Figure 6).

#### Disease-consensus index DCI

In this study DCI value ranges from 0.233 to 0.000. DCI values are found to be the highest in *Citrus limon* (0.233). Followed by *Crataegus aronia* (0.071), *Citrus aurantifolia* (0.063), *Crataegus oxycantha* (0.048), *Coccinia grandis* (0.047), *Butea monosperma* (0.044), *Dodonaea viscosa* (0.042), *Carex baccans* (0.034), *Caralluma tuberculata* (0.025), *Allium cepa* (0.022), *Acorus calamus* (0.020), *Bidens pilosa* (0.019), *Gnaphalium uliginosum* (0.018), *Cissus simsiana* (0.017), *Amaranthus spinosus* (0.015) and the lowest value observed in *Syzygium aromaticum* (0.000) (Supplementary Table 1).

#### RFC (relative frequency of citation)

RFC value ranges from 0.08 to 1.08. The highest RFC value was recorded for Bidens biternata (1.08), followed by Marrubium vulgare (0.97), Stevia rebaudiana, Syzygium aromaticum, Rheum ribes, Phytolacca dioica, Heliotropium lasiocarpum, Foeniculum vulgare, Phyllanthus emblica (0.54) and lowest value was observed for Allium cepa (0.08) (Supplementary Table 1).

#### Systematic reviews of herbal medicines used for hypertension therapies

The ethnobotanical data is compared with 31 published articles on the use of medicinal plants for hypertension. The analysis showed that some medicinal herbal plants are used for same purposes globally, but some novel uses were also recorded. Out of 192 plant species for hypertension, 81 species were reported with similar uses, while 112 species were documented for hypertension for the first time. These newly reported species for hypertension should be further investigated for detailed clinical and phytochemical studies (Supplementary Table 1). Some potential species reported first time against hypertension include *Abrus precatorius, Amarathus spinosus, Cissus simsiana, Citrus aurantifolia, Ficus palmata, Senna tora, Teucrium rogleanom, Valeriana officinalis, Ziziphus mauritiana* and others.

#### Phytochemical data of antihypertensive plants

The active phytochemicals of the plants have been shown in (Supplementary Table 1). These phytochemicals have variety of pharmacological activities that are used to treat hypertension. In this study documentation of plants species indicates, few species are pharmacologically assessed in the literature while other plant species needs more screening in future.

## Discussion

In communities of Northern Pakistan people rely mostly on herbal medicines for treatment of various diseases. Mostly the herbal remedies are practiced in the rural communities (Ibrar et al., 2007). Most of the local participants who showed the interest in traditional medicinal knowledge belong to the older age group (Ahmad et al., 2015). Local healers used a large number of different herbs for the treatment of hypertension, and seemed to play a relevant role in the management of hypertension in rural communities, which has important implications for health care workers. Majority of the informants stated that the transmission knowledge about usage of medicinal plants was not efficient due to lack of interest shown by the younger people. It was also observed that some people avoid to used traditional medicines due to availability and convenience of allopathic drugs (Kayani et al., 2015).

The most dominant family reported in terms of medicinal plants was Asteraceae. Of about 350,000 species of identified flowering plants, nearly 10% belong to Asteraceae, and almost every environment in different regions (Barker et al., 2008). This fact could possibly describe the usual occurrence of plant family Asteraceae. Alongside the Lamiaceae has maximum proportion in ethno medicine (Amira and Okubadejo, 2007). However, in study area, more species belonging to Apiaceae and Fabaceae were used as reported in previous literature by Asase et al. (2010).

Participants recognize diverse kinds of plant species with a variability of medicinal properties present in a single family (Abbasi et al., 2013a). Another reason of high citation of Lamiaceae and Asteraceae may be their higher occurrence in mountainous areas as reported in earlier studies (Pieroni, 2008; Mustafa et al., 2011).

Among the medicinal plant species herbs were most commonly used, because of the large number of species naturally abundant in these geographical regions (Abbasi et al., 2013a; Butt et al., 2015) and easily accessible to communities residing in these areas (Tabuti et al., 2003; Uniyal et al., 2006; Sanz-Biset et al., 2009). Herbs are easily accessible having high healing potential and yield secondary metabolites having therapeutical properties against diseases (Bano et al., 2014a; Yaseen et al., 2015a). It was noted that herbalists utilized herbs commonly for formulations due to ease in collection and availability (Uniyal et al., 2006).

In this study leaves were the major plant parts used. It was found that leaves preferably used in treatment of ailments due to significant amount of bioactive compounds present in the leaves (Chaudhary et al., 2010; Rashid et al., 2015). Similar results were reported from studies carried out the in previous literature (Mahishi et al., 2005; Abo et al., 2008; Shil et al., 2014) The flower, stem and leaves of different medicinal plants are used for curing various ailments like hypertension, digestive disorders, fever and others (Lev, 2000; Leporatti and Ivancheva, 2003).

Decoction was the most common method of utilization in present study, this result is in accord with the previous studies (Nadembega et al., 2011; Rehecho et al., 2011). In Pakistan most people prefer to use decoctions of herbal medicines (Mahmood et al., 2011; Khan et al., 2015). In our study *Mentha longifolia* leaf extract made from the leaves are used for treating hypertension this result is similar with previous study of (Hwang et al., 1987) in which aqueous-methanolic extract of this plant, showed a significant decrease in the blood pressure and heart rate. *Tinospora malabarica, Sonchus asper, Cestrum racemosum, Callisia gracilis*, and *Cymbopogon citratus* were also used against hypertension in other areas (Poffenberger and Singh, 1992; Steyn et al., 2001; Dharmananda, 2012). In high altitudes areas in winter season, dried powder is favored for curing different diseases (Ahmad et al., 2014; Bano et al., 2014b; Kayani et al., 2014). The most common method of administration of herbal drugs was oral (70%), like in other areas (Hammond et al., 1998; De-La-Cruz et al., 2007). Herbal medicines are mostly bitter in taste and prepared by mixing with sugar and honey (Sadeghi et al., 2014). In study area, the dosage of herbal remedies varied, like found in others studies (Ahmed et al., 2015). Many plants have been reported in previous literature for treating hypertension, blood pressure diseases such as *Allium sativum, Berberis vulgaris, Coriandrum sativum* and various other similar plants have been identified in present study are reported for curing hypertension (Sher et al., 2016).

DCI was evaluated in present study to analyze the disease consensus of informants for traditional remedies of hypertension (Choudhury et al., 2015; Yaseen et al., 2015b). Species having higher DCI value can be preferred for further future studies (Andrade-Cetto and Wiedenfeld, 2011). Pharmacological studies show that *Allium sativum, Lepidium sativum*, and *Ocimum basilicum, Mentha sp., Trigonella foenum-graecum, Urtica dioica, Olea species*, and *Eucalyptus globulus* are effective species in treating hypertension (Jouad et al., 2001; El-Hilaly et al., 2003; Gurib-Fakim, 2006). The root infusion of *Acorus calamus* is used as plant for anti-hypertension properties in this study which is in accord with the study of (Patel et al., 2012) who reported antihypertensive effect of rhizome part of *Acorus calamus* on renal artery occlusion induced hypertension in rats.

RFC refers the local importance of each plant species with reference to informant who cited this specie (Vitalini et al., 2013). The reason for high RFC value may be the easy availability of species, wide distribution and high medicinal properties for treating various ailments (Mukherjee and Wahile, 2006). *Foeniculum vulgare, Nerium oleander, Olea europaea, Allium sativum, Mentha sp., Eucalyptus globulus, Nigella sativa*, and *Lepidium sativum* were documented for the first time for treating hypertension (Eddouks et al., 2002; Zaoui et al., 2002) (Supplementary Table 1).

The comparison of our results shows broad variations with previous studies regarding JI when the findings of these studies were compared with the present work (Leonti et al., 2003; Ladio et al., 2007). In our study JI varies from 0 to 27.04%. The highest degree of similarity was recorded with (Eddouks et al., 2002; Tahraoui et al., 2007), while lowest similarity was found with (Mansoor, 2001a; Somova et al., 2003; Abel and Busia, 2005; Ahmad et al., 2005; Tee et al., 2010). The phytochemicals have variety of pharmacological activities that are used to treat hypertension. The study indicates many important medicinal plant species which are pharmacologically active but still unexplored and need to be explored further.

## Conclusion

The present study gave an overview about traditional medicinal knowledge of plants as anti-hypertensive drug. 192 medicinal plant species belonging to 77 families were reported to be in present study to treat hypertension. It was first attempt to document ethno-botanical information using quantitative approaches on hypertension in the study area. The leaves were reported to be most used plant part (55.1%) while herbs were the most used life form (54%) and decoction being the most common mode of administration (72 reports). The quantitative approaches such as Family importance value (FIV), Relative Frequency of Citation (RFC) and Disease Consensus index (DCI) were used to assess the importance of traditional knowledge obtained in the study used in the present study. Highest DCI and FIV values were reported for Rutaceae and Lamiaceae. Meanwhile there are so many of these naturally occurring plant substances cover a wide range they offers a good opportunity of delivering useful medicinal complexes for the management of hypertension. To the best of our knowledge, *Ficus palmata, Senna tora, Teucrium polium, Valeriana officinalis*, and *Ziziphus mauritiana* were recorded for the first time as anti-hypertensive medicinal drugs. The study provides basic leads for future pharmacological and phytochemical investigation to explore the potential of such plants in herbal drug discovery. It is thus recommended that strategies for cultivation and conservation of important species be designed.

## Author contributions

KM carried out field surveys and data collection. MZ and NR helped in analysis of data while MA and RB revised the manuscript critically to its present form. AT, RU, AA, AS, SS, and SNS helped in the revision of the manuscript and helps in checking the consistency of data. All authors read the final manuscript and agreed to its submission.

### Conflict of interest statement

The authors declare that the research was conducted in the absence of any commercial or financial relationships that could be construed as a potential conflict of interest.
