# Supplementary material for: Ethnobotany of Anti-hypertensive Plants Used in Northern Pakistan
Source: Front Pharmacol. 2018 Jul 24;9:789. doi: 10.3389/fphar.2018.00789 (PMC6066661; doi:10.3389/fphar.2018.00789)
Supplement: Supplementary file 1 [file Table_1.docx]

**Supplementary Table 1|** List of Medicinal Plants used against hypertension

| **Family**  **Scientific name**  **Voucher No** | **Local name**  **(Common name)** | **Life form** | **Part used** | **Preparation of remedy for hypertension** | **Chemicals** | **FC** | **RFC** | **DCI** | **Comparative studies Dissimilar ○**  **Similar ●** | **Previously reported use** |
| --- | --- | --- | --- | --- | --- | --- | --- | --- | --- | --- |
| Acoraceae  *Acorus calamus* L.  KM03 | Waj  (Vacha) | Herb | Root | Infusion of Root | Z-3-(2,4,5-trimethoxy phenyl)-2-propenal and a new phenyl indane Sesquiterpenoids ([Saxena, 1986](#_ENREF_226);[Nawamaki and Kuroyanagi, 1996](#_ENREF_185)) | 25 | 0.10 | 0.020 | 1 ○, 2 ○, 3 ○, 4 ○, 5 ○, 6 ○, 7 ○, 8 ○, 9 ○, 10 ○, 11 ○, 12 ●, 13 ○, 14 ○, 15 ○, 16 ○, 17 ○, 18 ●, 19 ○, 20 ●, 21 ○, 22 ○, 23 ○, 24 ○, 25 ○, 26 ○, 27 ○, 28 ○, 29 ○, 30 ●, 31 ○ | Plant for treatment of (12) medicinal purposes in the area, (18) digestive disorders and chronic dysentery, (20) for various medicinal purposes in the area, (30) colic, diarrhea. |
| Amaranthaceae  *Amaranthus spinosus* L.  KM09 | Khardar Julai  (Prickly amaranth) | Herb | Leaf, Whole plant | Fresh Leaf cooked and used as a vegetable. | Steroids Alkaloids Terpenoids ([Tatiya et al., 2007](#_ENREF_255)) | 25 | 0.10 | 0.015 | 1 ○, 2 ○, 3 ○, 4 ○, 5 ○, 6 ○, 7 ○, 8 ○, 9 ○, 10○, 11 ○, 12 ○, 13 ○, 14 ○, 15 ○, 16 ○, 17 ●, 18 ●, 19 ○, 20 ○, 21 ○, 22 ○, 23 ○, 24 ○, 25 ○, 26 ○, 27 ○, 28 ○, 29 ○, 30 ○, 31 ● | Plant is used in treatment of (17) eye vision, (18) Biliousness, (31) pot herb and fodder. |
| Amaranthaceae  *Gomphrena globosa* L.  KM91 | Gule Makhmal  (Bachelor button) | Herb | Leaf | Leaf tea | Alkaloids, flavonoids ([Dinda et al., 2004](#_ENREF_77)) | 54 | 0.22 | 0.003 | 1 ○, 2 ○, 3 ○, 4 ○, 5 ○, 6 ○, 7 ○, 8 ○, 9 ○, 10 ○, 11 ○, 12 ○, 13 ○, 14 ○, 15 ○, 16 ○, 17 ○, 18 ○, 19 ○, 20 ○, 21 ○, 22 ○, 23 ○, 24 ○, 25 ○, 26 ○, 27 ○, 28 ○, 29 ○, 30 ○, 31○ | Not reported |
| Amaranthaceae Haloxylon scoparium Pomel KM92 | Eremt, Assay | Shrub | Leaf, Flower | Leaf decoction and Leaf extraction . | Flavonol, flavanones, dihydroflavonol ([Lamchouri et al., 2012](#_ENREF_1)) | 51 | 0.20 | 0.003 | 1 ○, 2 ○, 3 ○, 4 ●, 5 ○, 6 ○, 7 ○, 8 ○, 9 ○, 10 ○, 11 ○, 12 ○, 13 ○, 14 ○, 15 ○, 16 ○, 17 ○, 18 ○, 19 ○, 20 ○, 21 ○, 22 ○, 23 ○, 24 ○, 25 ○, 26 ○, 27 ○, 28 ○, 29 ○, 30 ○, 31○ | The plant is used in the treatment of (4) diabetes mellitus, hypertension and cardiac diseases |
| Amaryllidaceae Allium cepa L. KM05 | Pyaz  (Onion, sogan) | Herb | Seed and Fruits | Fruit are taken orally as a medicine.  Decoction of seed is also effective | Proteins, alkaloids, saponins, flavonoids, acid compounds, reducing sugars and oils are present ([Shenoy et al., 2009](#_ENREF_230)) | 21 | 0.08 | 0.022 | 1 ○, 2 ○, 3 ●, 4 ●, 5 ●, 6 ○, 7 ○, 8 ○, 9 ○, 10 ○, 11 ○, 12 ○, 13 ○, 14 ○, 15 ○, 16 ●, 17 ○, 18 ○, 19 ○, 20 ○, 21 ○, 22 ○, 23 ○, 24 ○, 25 ○, 26 ○, 27 ○, 28 ●, 29 ○, 30 ●, 31○ | Plant for (3) diabetes, cardiac and renal diseases, (4) hypertension and cardiac diseases, (5) hypertension and diabetes, (16) dermatological and topical diseases, (28) hypertension, (30) Stimulant, diuretic, aphrodisiac and vegetables |
| Amaryllidaceae Allium sativum L. KM06 | Lehsan  (Garlic, Touma, Sarimsak, Lehsan) | Herb | Bulb, Root and Whole plant | Juice of the bulbs is taken orally | Flavonoids,sulphur containing compounds: diallyl sulphate, alliin, ajoene, allicin ([Mikail, 2010](#_ENREF_173)) | 28 | 0.11 | 0.011 | 1 ○, 2 ○, 3 ○ , 4 ●, 5 ●, 6 ○, 7 ○, 8 ○, 9 ●, 10 ●, 11 ○, 12 ○, 13 ○, 14 ○, 15 ○, 16 ●, 17 ○, 18 ○, 19 ○, 20 ○, 21 ○, 22 ○, 23 ○, 24 ○, 25 ○, 26 ○, 27 ○, 28 ●, 29 ○, 30 ●, 31○ | Plant is for treatment of (4) hypertension and cardiac diseases, (5) hypertension and diabetes, (9) hypertension, (10) hypertension, (16) gastrointestinal disorder, (28) hypertension, (30) heart diseases, asthma and whooping  Cough and vegetables |
| Anacardiaceae  *Mangifera indica* L.  KM117 | Aam  (Mango) | Tree | Leaf | Leaf extract or infusion | Alkaloids, gums, phenols, saponins, gums, tannins, flavonoids, steroids and xanthoproteins ([Rajan et al., 2011](#_ENREF_206)) | 70 | 0.28 | 0.037 | 1 ○, 2 ○, 3 ○, 4 ○, 5 ○, 6 ○, 7 ○, 8 ○, 9 ○, 10 ○, 11 ○, 12 ○, 13 ○, 14 ○, 15 ○, 16 ○, 17 ○, 18 ○, 19 ○, 20 ○, 21 ○, 22 ○, 23 ○, 24 ○, 25 ○, 26 ○, 27 ○, 28 ○, 29 ○, 30 ○, 31○, | Not reported |
| Annonaceae *Rollinia mucosa* (Jacq.) Baill.  KM11 | Shareefa  (Pinha) | Tree | Leaf& Seed | Scorch of Leaf and Seed | Isoquinoline alkaloids and lignanins ([Chen et al., 1996](#_ENREF_64)) | 54 | 0.22 | 0.006 | 1 ○, 2 ○, 3 ○, 4 ○, 5 ○, 6 ○, 7 ○, 8 ○, 9 ○, 10 ○, 11 ○, 12 ○, 13 ○, 14 ○, 15 ○, 16 ○, 17 ○, 18 ○, 19 ○, 20 ○, 21 ○, 22 ○, 23 ○, 24 ○, 25 ○, 26 ○, 27 ○, 28 ○, 29 ○, 30 ○, 31 ○ | Not reported |
| Apiaceae  *Apium graveolens* L.  KM12 | Ajmod  (Celery, soepgroen  te) | Herb | Aerial parts, Leaf, Root | Infusion of Leaf and Root are taken orally. | Fatty oil with the fatty acids: petroselenic , oleic, linoleic, linolenic, palmitic acids. Phthalides andespecially sedanenaloide ([Sowbhagya, 2014](#_ENREF_243)) | 108 | 0.43 | 0.004 | 1 ○, 2 ○, 3 ○, 4 ○, 5 ○, 6 ●, 7 ○, 8 ○, 9 ○, 10 ○, 11 ○, 12 ○, 13 ○, 14 ○, 15 ○, 16 ○, 17 ○, 18 ○, 19 ○, 20 ○, 21 ○, 22 ○, 23 ○, 24 ○, 25 ○, 26 ○, 27 ○, 28 ○, 29 ○, 30 ○, 31○ | Plant is used in treatment of (6) antiacid, stomach pain,  menstruation pain, diarrhea and  kidney problems |
| Apiaceae  *Centella asiatica* (L.) Urb.  KM40 | Brahmi Booti  (Mandukparni) | Herb | Whole plant | Whole plant juice | Triterpenoid and saponin ([Mangas et al., 2008](#_ENREF_161)), | 108 | 0.43 | 0.003 | 1 ○, 2 ○, 3 ○, 4 ○, 5 ○, 6 ○, 7 ○, 8 ○, 9 ●, 10 ○, 11 ○, 12 ○, 13 ○, 14 ○, 15 ○, 16 ○, 17 ○, 18 ○, 19 ○, 20 ○, 21 ○, 22 ○, 23 ○, 24 ○, 25 ○, 26 ○, 27 ○, 28 ○, 29 ○, 30 ○, 31○ | Plant is used in treatment of (9) hypertension |
| Apiaceae  *Coriandrum sativum* L.  KM62 | Dhanya  (Kasbour, coriander) | Herb | Fruit, Seed | Fruit extract and infusion of Seeds | Tannins, flavonoids, alkaloids, saponins ([Sreelatha et al., 2009](#_ENREF_244)) | 32 | 0.13 | 0.004 | 1 ○, 2 ○, 3 ○, 4 ●, 5 ●, 6 ○, 7 ○, 8 ●, 9 ○, 10 ○, 11 ○, 12 ○, 13 ○, 14 ○, 15 ○, 16 ●, 17 ○, 18 ○, 19 ○, 20 ○, 21 ○, 22 ○, 23 ○, 24 ○, 25 ○, 26 ○, 27 ○, 28 ○, 29 ○, 30 ●, 31○ | Plant is used in treatment of (4) diabetes mellitus, hypertension and cardiac diseases, (5) hypertension and diabetes, (8) hypertension, (16) gastrointestinal, wounds, cuts, narcotic, tonic, tumor,  anticancer and stimulant, (30) Carminative and vegetables |
| Apiaceae  *Cuminum cyminum* L.  KM69 | Jeero  (Cumin, jeera) | Herb | Leaf | Leaf decoction | Tannins, flavonoids and alkaloids ([Bettaieb et al., 2010](#_ENREF_51)) | 108 | 0.43 | 0.003 | 1 ○, 2 ○, 3 ●, 4 ○, 5 ○, 6 ○, 7 ○, 8 ○, 9 ○, 10 ○, 11 ○, 12 ○, 13 ○, 14 ○, 15 ○, 16 ○, 17 ○, 18 ○, 19 ○, 20 ○, 21 ○, 22 ○, 23 ○, 24 ○, 25 ○, 26 ○, 27 ○, 28 ○, 29 ○, 30 ○, 31○ | Plant is used in the treatment of (3) diabetes, cardiac and renal diseases |
| Apiaceae  *Foeniculum vulgare* Mill.  KM85 | Sonf  (Saunf) | Herb | Seed | Collect healthy Seed, wash thoroughly i and dry at room temperature for 15 days. Then 20 g Seed powdered and soaked in 100 ml water | Alkaloids, flavonoids, tannins, saponins and cardiac  Glycosides ([Kaur and Arora, 2009](#_ENREF_131)) | 135 | 0.54 | 0.002 | 1 ○, 2 ○, 3 ○, 4 ●, 5 ●, 6 ●, 7 ○, 8 ●, 9 ○, 10 ○, 11 ○, 12 ○, 13 ○, 14 ○, 15 ○, 16 ●, 17 ○, 18 ○, 19 ○, 20 ○, 21 ○, 22 ○, 23 ○, 24 ○, 25 ○, 26 ○, 27 ○, 28 ○, 29 ○, 30 ○, 31○ | The plant is used in the treatment of (4) diabetes mellitus, hypertension and cardiac diseases, (5) hypertension and diabetes, (6) cancer, conjunctivitis,  gastritis, nursing and relaxant , (8) hypertension, (16) gastrointestinal disorder and urinary complaints |
| Apiaceae  *Pimpinella hazariensis* H. Wolff. KM148 | (Aniseed, Anise, habbat halwa) | Herb | Seed &Fruit | Take 2 cup of hot water add 3 teaspoons of grinded plant mix well and use twice a day. | None | 54 | 0.22 | 0.009 | 1 ○, 2 ○, 3○ , 4 ○, 5 , 6 ○, 7 ○, 8 ○, 9 ○, 10 ○, 11 ○, 12 ○, 13 ○, 14 ○, 15 ○, 16 ○, 17 ○, 18 ○, 19 ○, 20 ○, 21 ○, 22 ○, 23 ○, 24 ○, 25 ○, 26 ○, 27 ○, 28 ○, 29 ○, 30 ○, 31○ | Not Reported |
| Apiaceae  *Visnaga daucoides* Gaertn  KM10 | Ammi  (Bisnaga, Ajwine) | Herb | Fruits | Fruit extract is prepared and taken with honey | None | 29 | 0.12 | 0.012 | 1 ○, 2 ○, 3 ○, 4 ●, 5 ●, 6 ○, 7 ○, 8 ○, 9 ○, 10○, 11 ○, 12 ○, 13 ○, 14 ○, 15 ○, 16 ○, 17 ○, 18 ○, 19 ○, 20 ○, 21 ○, 22 ○, 23 ○, 24 ○, 25 ○, 26 ○, 27 ○, 28 ○, 29 ○, 30 ○, 31 ○ | Plant is used in treatment of (4) diabetes, hypertension and cardiac diseases, (5) hypertension and diabetes |
| Apocynaceae  *Alstonia boonei* De Wild.  KM08 | (God's tree, Onyame dua) | Herb | Buds | Dried buds are used to make decoction and drink 1 cup after every meal. | Alkaloids, triterpenoids ([Adebayo et al., 2004](#_ENREF_9)) | 23 | 0.09 | 0.014 | 1 ○, 2 ○, 3 ○, 4 ○, 5 ○, 6 ○, 7 ○, 8 ○, 9 ○, 10 ○, 11 ○, 12 ○, 13 ○, 14 ○, 15 ○, 16 ○, 17 ○, 18 ○, 19 ○, 20 ○, 21 ○, 22 ○, 23 ○, 24 ○, 25 ○, 26 ○, 27 ○, 28 ○, 29 ○, 30 ○, 31○ | Not reported |
| Apocynaceae  *Catharanthus roseus* (L.) G.Don  KM39 | Ratanjot  (Kiua, Nitya kanagale) | Herb | Leaf | Juice of the fruit (about 6 teaspoons 3 times a day) is given to cure the disease. | Alkaloids, steroids, phenols, saponin, aminoacid, lipids and Ortho-dihydroxy phenols ([Govindasamy and Srinivasan, 2012](#_ENREF_98)) | 162 | 0.65 | 0.003 | 1 ○, 2 ○, 3 ○, 4 ○, 5 ○, 6 ○, 7 ○, 8 ●, 9 ○, 10 ○, 11 ○, 12 ○, 13 ○, 14 ○, 15 ○, 16 ○, 17 ○, 18 ○, 19 ○, 20 ○, 21 ○, 22 ○, 23 ○, 24 ○, 25 ○, 26 ○, 27 ○, 28 ○, 29 ○, 30 ○, 31○ | Plant is used in treatment of (8) hypertension |
| Apocynaceae  *Holarrhena pubescens* Wall. ex G. Don  KM99 | Inderjo  (Kurchi) | Shrub | Leaf | Leaf extract | Steroidal alkaloids and an androstane derivative ([Siddiqui et al., 1993](#_ENREF_236)) | 56 | 0.22 | 0.003 | 1 ○, 2 ○, 3 ○, 4 ○, 5 ○, 6 ○, 7 ○, 8 ○, 9 ○, 10 ○, 11 ○, 12 ○, 13 ○, 14 ○, 15 ○, 16 ○, 17 ○, 18 ○, 19 ○, 20 ○, 21 ○, 22 ○, 23 ○, 24 ○, 25 ○, 26 ○, 27 ○, 28 ○, 29 ○, 30 ○, 31○, | Not reported |
| Apocynaceae  *Nerium oleander* L.  KM135 | Kaneer  (Defla) | Shrub | Leaf | Extract of Leaf are mixed with oil | Polysaccharides, cardenolides, glycosides, triterpenoides ([Gupta and Mittal, 2010](#_ENREF_108)) | 54 | 0.22 | 0.008 | 1 ○, 2 ○, 3 ●, 4 ●, 5 ●, 6 ○, 7 ○, 8 ●, 9 ○, 10 ○, 11 ○, 12 ○, 13 ○, 14 ○, 15 ○, 16 ○, 17 ○, 18 ○, 19 ○, 20 ○, 21 ○, 22 ○, 23 ○, 24 ○, 25 ○, 26 ○, 27 ○, 28 ○, 29 ○, 30 ○,31○ | Plant is used in treatment of (3) diabetes, cardiac and renal diseaases, (4) diabetes, hypertension and cardiac diseases, (5) hypertension and diabetes, (8) hypertension |
| Apocynaceae  *Rauvolfia caffra* Sond.  KM158 | Sarab Gandh  (luofumu (H)) | Shrub | Stem , Root | Stem and Root are dried under shade and then ground to makepowder and mix with sugar and boiled | Alkaloids were the indolenine compounds raucaffrinoline, perakine, vomilenine, indole alkaloids peraksine and adihydroperaksine *(*[Nasser, 1983](#_ENREF_184)) | 27 | 0.11 | 0.029 | 1 ○, 2 ○, 3 ○, 4 ○, 5 ○, 6 ○, 7 ○, 8 ○, 9 ○, 10 ○, 11 ○, 12 ○, 13 ○, 14 ○, 15 ○, 16 ○, 17 ○, 18 ○, 19 ○, 20 ○, 21 ○, 22 ○, 23 ○, 24 ○, 25 ○, 26 ○, 27 ○, 28 ○, 29 ○, 30 ○, 31 ○ | Not reported |
| Apocynaceae  *Rauvolfia serpentina* (L.) Benth. ex Kurz  KM159 | Asraul  (Sharpagondha, Indian Snakeroot ) | Herb | Leaf, stems, Root | Soaked Leaf in vinegar and diluted in water is taken orally | Alkaloids, carbohydrates, flavonoids, glycosides, phlobatannins, phenols, resins, saponins sterols, tannins, terpenes([Harisaranraj et al., 2009](#_ENREF_112)) | 54 | 0.22 | 0.019 | 1 ○, 2 ○, 3 ○, 4 ○, 5 ○, 6 ○, 7 ○, 8 ○, 9 ○, 10 ○, 11 ○, 12 ○, 13 ○, 14 ○, 15 ○, 16 ○, 17 ○, 18 ○, 19 ○, 20 ○, 21 ○, 22 ○, 23 ○, 24 ○, 25 ○, 26 ○, 27 ○, 28 ○, 29 ○, 30 ○, 31 ○ | Not reported |
| Apocynaceae  *Rhazya stricta* Decne.  KM160 | Sanwar  (Eshoark, harishak) | Shrub | Leaf, Stem, Bark | Leaf and bark are crushed and are mixed and boiled in water and taken for 2-3 days. | Alkaloids (antirhine, geissoschizine, rhazimanine), flavonoids ([Gilani et al., 2007](#_ENREF_95)) | 81 | 0.32 | 0.004 | 1 ○, 2 ○, 3 ○, 4 ○, 5 ○, 6 ○, 7 ○, 8 ○, 9 ○, 10 ○, 11 ○, 12 ○, 13 ○, 14 ○, 15 ○, 16 ○, 17 ○, 18 ○, 19 ○, 20 ○, 21 ○, 22 ○, 23 ○, 24 ○, 25 ○, 26 ○, 27 ○, 28 ○, 29 ○, 30 ○, 31 ○ | Not reported |
| Araliaceae *Hydrocotyle javanica* Thunb. KM101 | Brahmi Booti | Herb | Leaf, Stems | Decoction and juice of Leaf and Stems | Alkaloids, flavonoids, phenols,  tannins, leucoanthocyanidins and cardiac glycosides ([Mandal et al., 2016](#_ENREF_160)) | 57 | 0.23 | 0.003 | 1 ○, 2 ○, 3 ○, 4 ○, 5 ○, 6 ○, 7 ○, 8 ○, 9 ○, 10 ○, 11 ○, 12 ○, 13 ○, 14 ○, 15 ○, 16 ○, 17 ○, 18 ○, 19 ○, 20 ○, 21 ○, 22 ○, 23 ○, 24 ○, 25 ○, 26 ○, 27 ○, 28 ○, 29 ○, 30 ○, 31○ | Not reported |
| Arecaceae  *Phoenix dactylifera* L.  KM145 | Khajoor  (Date palm) | Tree | Leaf, Fruit | Leaf are crushed and soaked in water to make infusion | Anthocyanins, phenolics, sterols, carotenoids, procyanidins, flavonoids ([Mossa et al., 1986](#_ENREF_177)) | 90 | 0.36 | 0.007 | 1 ○, 2 ○, 3 ○, 4 ○, 5 ●, 6 ○, 7 ○, 8 ○, 9 ○, 10 ○, 11 ○, 12 ○, 13 ○, 14 ○, 15 ○, 16 ○, 17 ○, 18 ○, 19 ○, 20 ○, 21 ○, 22 ○, 23 ○, 24 ○, 25 ○, 26 ○, 27 ○, 28 ○, 29 ○, 30 ○, 31○ | Plant is used in the treatment of (5) hypertension and diabetes |
| Arecaceae  *Trachycarpus fortunei* (Hook.) H. Wendl.  KM184 | Zungliusu’iab | Tree | Leaf | Leaf are dipped in water and boiled and decoction is for 3 weeks. | Asisovanillic acid,β-sitostero,dioscin,5-O-caffeoylshikimic acid, caffeic acid andrutin ([Lu et al., 2011](#_ENREF_154)) | 112 | 0.45 | 0.003 | 1 ○, 2 ○, 3 ○, 4 ○, 5 ○, 6 ○, 7 ○, 8 ○, 9 ○, 10 ○, 11 ○, 12 ○, 13 ○, 14 ○, 15 ○, 16 ○, 17 ○, 18 ○, 19 ○, 20 ○, 21 ○, 22 ○, 23 ○, 24 ○, 25 ○, 26 ○, 27 ○, 28 ○, 29 ○, 30 ○, 31 ○ | Not reported |
| Asclepiadacea  *Caudanthera edulis* (Edgew.) Meve & Liede KM34 | Koong  (Marmortk) | Shrub | Stem | A dried stem of the plant is boiled in water to form decoction and drink this decoction 1 cup daily to avoid the disease. |  | 67 | 0.27 | 0.009 | 1 ○, 2 ○, 3 ○, 4 ○, 5 ○, 6 ○, 7 ○, 8 ○, 9 ○, 10 ○, 11 ○, 12 ○, 13 ○, 14 ○, 15 ○, 16 ○, 17 ○, 18 ○, 19 ○, 20 ○, 21 ○, 22 ○, 23 ○, 24○, 25 ○, 26 ○, 27 ○, 28 ○, 29 ○, 30 ●, 31● | Plant is used in treatment of (30) diabetes and vegetable (31) carminative, anti-biabetic,  febrifuge and stomachic. |
| Asclepiadaceae  *Calotropis procera*  (Aiton) W.T. Aiton  KM29 | Aak  (Akk) | Herb | Leaf | Infusion made from Leaf | Cardenolides, flavonoids, and saponins ([Moustafa et al., 2010](#_ENREF_5)). | 87 | 0.35 | 0.003 | 1 ○, 2 ○, 3 ○, 4 ○, 5 ○, 6 ○, 7 ○, 8 ○, 9 ○, 10 ○, 11 ○, 12 ○, 13 ○, 14 ● , 15 ○, 16 ○ , 17 ○, 18 ●, 19 ○, 20 ○, 21 ○, 22 ○, 23 ○, 24 ○, 25 ○, 26 ○, 27 ○, 28 ○, 29 ○, 30 ●, 31○ | Plant is used in treatment of (14) expectorant, cough, cold and asthma, (18) malarial fever, Ulcer, Eczema and  Ring worms, (30) asthma and cough |
| Asclepiadaceae Caralluma tuberculata N.E.Br. [Meve & Liede](http://mpns.kew.org/mpns-portal/plantDetail?plantId=500640&query=Caralluma+tuberculata+&filter=&fuzzy=false&nameType=all&dbs=wcs) KM35 | Chaung  (Marmoot) | Herb | Whole plant | Tea makes from dried plant material and takes one cup daily before breakfast | Acylated pregnane glycosides ([Abdel-Sattar et al., 2008](#_ENREF_3)) | 27 | 0.11 | 0.025 | 1 ○, 2 ○, 3 ○, 4 ○, 5 ○, 6 ○, 7 ○, 8 ○, 9 ○, 10 ○, 11 ○, 12 ○, 13 ○, 14 ○, 15 ○, 16 ○, 17 ○, 18 ○, 19 ○, 20 ○, 21 ○, 22 ○, 23 ○, 24 ○, 25 ○, 26 ○, 27 ○, 28 ○, 29 ○, 30 ○, 31○ | Not reported |
| Asparagaceae  *Asparagus recemosus* Willd.  KM17 | Marchob  (Shatavari, shatamull) | Shrub | Root, Leaf | Root and Leaf extract | Steroidal saponins, isoflavones, Oligospirostanoside,cyclohydrocarbon, flavonoids and sterols ([Alok et al., 2013](#_ENREF_30)) | 59 | 0.24 | 0.005 | 1 ○, 2 ○, 3 ○, 4 ○, 5 ○, 6 ○, 7 ○, 8 ○, 9 ○, 10 ○, 11 ○, 12 ○, 13 ○, 14 ○, 15 ○, 16 ○, 17 ○, 18 ○, 19 ○, 20 ○, 21 ○, 22 ○, 23 ○, 24 ●, 25 ○, 26 ○, 27 ○, 28 ○, 29 ○, 30 ○, 31○ | Plant is for (24) food served on such occasions as new year or at funerals |
| Asteraceae  *Achillea millefolium* L.  KM02 | Biranjasif, Gomada  (Yarrow) | Herb | Flower and Leaf | Flower and Leaf are dipped whole night in water and taken twice a day. | Sabinene, β-pinene, 1,8-cineole, artemisia ketone, linalool, α-thujone, β-thujone, camphor, borneol, fenchyl acetate, bornyl acetate, (E)-β-caryophyllene, germacrene D, caryophyllene oxide and β-bisabolol([Orav et al., 2006](#_ENREF_191)) , | 22 | 0.09 | 0.017 | 1 ○, 2 ○, 3 ○, 4 ○, 5 ○, 6 ○, 7 ○, 8 ○, 9 ●, 10 ○, 11 ○, 12 ○, 13 ○, 14 ○, 15 ○, 16 ○, 17 ○, 18 ○, 19 ○, 20 ○, 21 ○, 22 ○, 23 ○, 24 ○, 25 ○, 26 ○, 27 ○, 28 ○, 29 ○, 30 ○, 31○ | Plant for treatment of (9) hypertension. |
| Asteraceae  *Artemisia scoparia* Waldst. & Kit.  KM14 | Afsanteen  (Chiba, wormwood) | Herb | Leaf, Fruits | Decoction of dried Leaf | Oleanolic acid, β-sitosterol, stigmasterol, and the four flavones artemetin, bonanzin, eupalitin and chrysosplenetin ([Tang et al., 2000](#_ENREF_252)) | 56 | 0.22 | 0.003 | 1 ○, 2 ○, 3 ○, 4 ○, 5 ○, 6 ○, 7 ○, 8 ○, 9 ○, 10 ○, 11 ○, 12 ○, 13 ○, 14 ○, 15 ○, 16 ●, 17 ○, 18 ○, 19 ○, 20 ○, 21 ○, 22 ○, 23 ○, 24 ●, 25 ○, 26 ○, 27 ○, 28 ○, 29 ○, 30 ○, 31○ | Plant is used in treatment of  (16) respiratory stimulant, anthelmintic,  purgative and against earache, (24) mycophilous |
| Asteraceae  *Artemisia sieversiana* Ehrh. ex Willd KM15 | Zoon  (Shih, chih, white mugwort) | Shrub | Aerial parts, Leaf, Root | The extract made from Leaf and Root | Lignans and Sesquiterpene ([Tan et al., 1998](#_ENREF_10)) | 57 | 0.23 | 0.003 | 1 ○, 2 ○, 3 ○, 4 ○, 5 ○, 6 ○, 7 ○, 8 ○, 9 ○, 10 ○, 11 ○, 12 ○, 13 ○, 14 ○, 15 ○, 16 ○, 17 ○, 18 ○, 19 ●, 20 ○, 21 ○, 22 ○, 23 ●, 24 ○, 25 ○, 26 ○, 27 ○, 28 ○, 29 ○, 30 ○, 31○ , 32 ○. | Plant is used in treatment of  (19) Pneumonia, Joints and boils, (23) burns and Veterinary:uses |
| Asteraceae  *Artemisia parviflora* [Roxb. ex D.Don](http://mpns.kew.org/mpns-portal/plantDetail?plantId=gcc-46794&query=Artemisia+parviflora+&filter=&fuzzy=false&nameType=all&dbs=gcc) Buch.-  KM16 | Jhau  (Kok-ka-ya, Dawna-pan) | Herb | Seed, Leaf, Fruits | Seed are grinded into fine powder and take this powder 2 table spoons per day. | Alkaloids, steroids, terpenoids, di- and triterpenoids, flavonoids, tannins, phenols and volatile oils ([Ahameethunisa and Hopper, 2012](#_ENREF_11)) | 58 | 0.23 | 0.003 | 1 ○, 2 ○, 3 ○, 4 ○, 5 ○, 6 ○, 7 ○, 8 ○, 9 ○, 10 ○, 11 ○, 12 ○, 13 ○, 14 ○, 15 ○, 16 ○, 17 ○, 18 ○, 19 ○, 20 ○, 21 ○, 22 ○, 23 ○, 24 ●, 25 ○, 26 ○, 27 ○, 28 ○, 29 ○, 30 ○, 31○ | Plant is for (24) food served on such occasions as new year or at funerals |
| Asteraceae  *Bidens biternata* (Lour.) Merr.& Sherff., KM20 | Chirchita  (Devil’s needle, Needle grass) | Herb | Aerial parts | Aerial part of plant are used to prepare extract. | Reducing sugar, glycosides, flavonoids, alkaloids, tannins, steroids, terpenoids, coumarins, saponins, anthraquinones, phlobatannins and iridoids ([Sukumaran et al., 2012](#_ENREF_6)) | 270 | 1.08 | 0.004 | 1 ○, 2 ○, 3 ○, 4 ●, 5 ○, 6 ○, 7 ○, 8 ○, 9 ○, 10 ○, 11 ○, 12 ○, 13 ○, 14 ○, 15 ○, 16 ○, 17 ○, 18 ○, 19 ○, 20 ○, 21 ○, 22 ○, 23 ○, 24 ○, 25 ○, 26 ○, 27 ○, 28 ○, 29 ○, 30 ○, 31○ | Plant is used in treatment of (4)hypertension and cardiac diseases |
| Asteraceae  *Bidens pilosa* L.  KM23 | Chirchita  (Devil’s needle, Needle grass) | Herb | Leaf | Infusion can be prepared by using ½ cup of plant Leaf in ½ liter of water and used this 1 cup 6 times a day. | Flavonoids ([Brandão et al., 1997](#_ENREF_56)) | 27 | 0.11 | 0.019 | 1 ○, 2 ○, 3 ○, 4 ○, 5 ○, 6 ●, 7 ○, 8 ○, 9 ○, 10 ○, 11 ○, 12 ○, 13 ○, 14 ○, 15 ○, 16 ○, 17 ○, 18 ○, 19 ○, 20 ○, 21 ○, 22 ○, 23 ○, 24 ○, 25 ○, 26 ●, 27 ○, 28 ○, 29 ○, 30 ○, 31○ | Plant is used in treatment of (6) stomach pain, menstruation  pain, scurvy, influenza,  prostate disturbances and  pneumonia, (26) hypertension |
| Asteraceae  *Cichorium intybus* L.  KM44 | Kasni  Cicoria | Herb | Leaf, Bark, stem | An infusion of 30 g of Leaf in 1 liter water is prepared and taken, 100 cc, two times a day. | Lactucin and Lactucopicrin, ([Bischoff et al., 2004](#_ENREF_54)) | 81 | 0.32 | 0.003 | 1 ○, 2 ○, 3 ○, 4 ○, 5 ○, 6 ○, 7 ○, 8 ●, 9 ○, 10 ○, 11 ○, 12 ○, 13 ○, 14 ○, 15 ○, 16 ○, 17 ○, 18 ○, 19 ○, 20 ○, 21 ○, 22 ○, 23 ○, 24 ○, 25 ○, 26 ○, 27 ○, 28 ○, 29 ○, 30 ○, 31○ | Plant is used in treatment of (8) hypertension |
| Asteraceae  *Centaurea benedicta* L.  KM54 |  | Herb | Flower, Leaf | Leaf (about 20 g) are boiled in 200 ml water for about 20 min., strained, and this liquid (about 6 teaspoons twice a day) | None | 27 | 0.11 | 0.003 | 1 ○, 2 ○, 3 ○, 4 ○, 5 ○, 6 ○, 7 ○, 8 ○, 9 ○, 10 ○, 11 ○, 12 ○, 13 ○, 14 ○, 15 ○, 16 ○, 17 ○, 18 ○, 19 ○, 20 ○, 21 ○, 22 ○, 23 ○, 24 ○, 25 ○, 26 ○, 27 ○, 28 ○, 29 ○, 30 ○, 31○ | Not reported |
| Asteraceae  *Crepis titingana* Balb.  KM68 | Ahiro | Herb | Leaf | Leaf decoction | None | 36 | 0.14 | 0.028 | 1 ○, 2 ○, 3 ○, 4 ○, 5 ○, 6 ○, 7 ○, 8 ○, 9 ○, 10 ○, 11 ○, 12 ○, 13 ○, 14 ○, 15 ○, 16 ○, 17 ○, 18 ○, 19 ○, 20 ○, 21 ○, 22 ○, 23 ○, 24 ○, 25 ○, 26 ○, 27 ○, 28 ○, 29 ○, 30 ○, 31○ | Not reported |
| Asteraceae  *Cynara cardunculus* L.  KM73 | Oont Katara  () | Herb | Fruit | Dried fruit decoction | Polyphenols and Phenolic acids([Pandino et al., 2011](#_ENREF_196)) | 38 | 0.15 | 0.004 | 1 ○, 2 ○, 3 ●, 4 ○, 5 ●, 6 ○, 7 ○, 8 ○, 9 ○, 10 ○, 11 ○, 12 ○, 13 ○, 14 ○, 15 ○, 16 ○, 17 ○, 18 ○, 19 ○, 20 ○, 21 ○, 22 ○, 23 ○, 24 ○, 25 ○, 26 ○, 27 ○, 28 ○, 29 ○, 30 ○, 31○ | Plant is used in the treatment of (3) diabetes, cardiac and renal diseases, (5) hypertension and diabetes |
| Asteraceae  *Dittrichia viscosa* (L.) Greuter  KM102 | (Inula, Terrahla, Magraman) | Herb | Leaf, Root | Leaf and stem are dipped in water for 2 days to make an infusion | Triterpenoids ([Grande et al., 1992](#_ENREF_100)) | 54 | 0.22 | 0.004 | 1 ○, 2 ○, 3 ○, 4 ○, 5 ●, 6 ○, 7 ○, 8 ○, 9 ○, 10 ○, 11 ○, 12 ○, 13 ○, 14 ○, 15 ○, 16 ○, 17 ○, 18 ○, 19 ○, 20 ○, 21 ○, 22 ○, 23 ○, 24 ○, 25 ○, 26 ○, 27 ○, 28 ○, 29 ○, 30 ○, 31○ | Plant is used in the treatment of (5) hypertension and diabetes |
| Asteraceae  *Glebionis coronaria* (L.) Cass. [ex Spach](http://mpns.kew.org/mpns-portal/plantDetail?plantId=gcc-28875&query=Glebionis+coronaria+&filter=&fuzzy=false&nameType=all&dbs=gcc)  KM43 | (Santa Maria (s)) | Herb | Aerial part | A standard decoction of aerial parts of plant is prepared and taken orally,150cc, three times per day | Phenolics compound ([Hosni et al., 2013](#_ENREF_6)) | 54 | 0.22 | 0.003 | 1 ○, 2 ○, 3 ○, 4 ○, 5 ○, 6 ○, 7 ○, 8 ○, 9 ○, 10 ○, 11 ○, 12 ○, 13 ○, 14 ○, 15 ○, 16 ○, 17 ○, 18 ○, 19 ○, 20 ○, 21 ○, 22 ○, 23 ○, 24 ○, 25 ○, 26 ○, 27 ○, 28 ○, 29 ○, 30 ○, 31○ | Not reported |
| Asteraceae  *Gnaphalium uliginosum* L.  KM83 | (Cudweed) | Herb | Leaf, Flower | Fruit will be dry and dipped in boiled water | Phenolics ([Shikov et al., 2010](#_ENREF_12)) | 56 | 0.22 | 0.018 | 1 ○, 2 ○, 3 ○, 4 ○, 5 ○, 6 ○, 7 ○, 8 ○, 9 ○, 10 ○, 11 ○, 12 ○, 13 ○, 14 ○, 15 ○, 16 ○, 17 ○, 18 ○, 19 ○, 20 ○, 21 ○, 22 ○, 23 ○, 24 ○, 25 ○, 26 ○, 27 ○, 28 ○, 29 ○, 30 ○, 31○ | Not reported |
| Asteraceae  *Helianthus annuus* L.  KM93 | Suraj Mukhi  (Hiongngidkui, Baya’a Chems) | Shrub | Leaf, Aerial parts | Leaf and Flower are dried | Flavonoids ([Macías et al., 1997](#_ENREF_156)) | 52 | 0.21 | 0.022 | 1 ○, 2 ○, 3 ●, 4 ●, 5 ○, 6 ○, 7 ○, 8 ○, 9 ○, 10 ○, 11 ○, 12 ○, 13 ○, 14 ○, 15 ○, 16 ○, 17 ○, 18 ○, 19 ○, 20 ○, 21 ○, 22 ○, 23 ○, 24 ○, 25 ○, 26 ○, 27 ○, 28 ○, 29 ○, 30 ○, 31○ | The plant is used in the treatment of (3) diabetes, cardiac and renal diseases, (4) diabetes mellitus, hypertension and cardiac diseases |
| Asteraceae  *Lactuca sativa* L.  KM108 | Salad  (Alface) | Herb | Leaf, Root | Powder of the root (about 200g and 3 times a day) is given with water | Sesquiterpene lactones, phytols, carotenoids ([Mahmoud et al., 1986](#_ENREF_159)) | 62 | 0.25 | 0.003 | 1 ○, 2 ○, 3 ○, 4 ●, 5 ○, 6 ○, 7 ○, 8 ○, 9 ○, 10 ○, 11 ○, 12 ○, 13 ○, 14 ○, 15 ○, 16 ○, 17 ○, 18 ○, 19 ○, 20 ○, 21 ○, 22 ○, 23 ○, 24 ○, 25 ○, 26 ○, 27 ○, 28 ○, 29 ○, 30 ○, 31○ | Plant used in the treatment of (4) diabetes, hypertension and cardiac disease |
| Asteraceae  *Lactuca serriola* L.  KM109 | Kahu  (Khouss) | Herb | Leaf, Seed | Leaf and Root extract | Sesquiterpenes ([Marco et al., 1992](#_ENREF_9)) | 63 | 0.25 | 0.003 | 1 ○, 2 ○, 3 ○, 4 ○, 5 ○, 6 ○, 7 ○, 8 ○, 9 ○, 10 ○, 11 ○, 12 ○, 13 ○, 14 ○, 15 ○, 16 ○, 17 ○, 18 ○, 19 ○, 20 ○, 21 ○, 22 ○, 23 ○, 24 ○, 25 ○, 26 ○, 27 ○, 28 ○, 29 ○, 30 ○, 31○ | Not reported |
| Asteraceae  *Matricaria aurea* (Loefl.) Sch.Bip.  KM120 | Babuna  (Camomilla aurea (Italian)) | Shrub | Flower, shoots | Infusion of flower |  | 54 | 0.22 | 0.006 | 1 ○, 2 ○, 3 ○, 4 ○, 5 ○, 6 ○, 7 ○, 8 ○, 9 ○, 10 ○, 11 ○, 12 ○, 13 ○, 14 ○, 15 ○, 16 ○, 17 ○, 18 ○, 19 ○, 20 ○, 21 ○, 22 ○, 23 ○, 24 ○, 25 ○, 26 ○, 27 ○, 28 ○, 29 ○, 30 ○, 31○, | Not reported |
| Asteraceae  *Matricaria chamomilla* L.  KM121 | Babuna  (Babounge alhmir) | Herb | Fruits | Fruit extract | Glucoside, ß-sitosterol oleanolic acid, stigmasterol, and ([Ahmad and Misra, 1997](#_ENREF_12)) | 72 | 0.29 | 0.002 | 1 ○, 2 ○, 3 ○, 4 ○, 5 ○, 6 ●, 7 ○, 8 ○, 9 ○, 10 ○, 11 ○, 12 ○, 13 ○, 14 ○, 15 ○, 16 ○, 17 ○, 18 ○, 19 ○, 20 ○, 21 ○, 22 ○, 23 ○, 24 ○, 25 ○, 26 ○, 27 ○, 28 ○, 29 ○, 30 ○, 31○ |  |
| Asteraceae  *Sonchus asper* (L.) Hill  KM172 | Phut kanda  (Didhi) | Herb | Fruits, Stem | Leaf buds are boiled in water for about 20 min, strained and the juice | Sesquiterpene lactones glucosides ([Helal et al., 2000](#_ENREF_6)) | 106 | 0.42 | 0.005 | 1 ○, 2 ○, 3 ○, 4 ○, 5 ○, 6 ○, 7 ○, 8 ○, 9 ○, 10 ○, 11 ○, 12 ○, 13 ○, 14 ○, 15 ○, 16 ●, 17 ●, 18 ○, 19 ○, 20 ○, 21 ○, 22 ○, 23 ○, 24 ○, 25 ○, 26 ○, 27 ○, 28 ○, 29 ○, 30 ●, 31 ○ | The plant is used in (16) livestock for  enhancing lactation, (17) fever and constipation, (30) fodder |
| Asteraceae  *Stevia rebaudiana* (Bertoni)  KM174 | (Sweetleaf) | Herb | Stem | Methanolic extract of stem | Alkaloids, flavonoids, tannins, phenolic compounds ([Tadhani and Subhash, 2006](#_ENREF_249)) | 135 | 0.54 | 0.001 | 1 ○, 2 ○, 3 ○, 4 ○, 5 ○, 6 ○, 7 ○, 8 ○, 9 ○, 10 ○, 11 ○, 12 ○, 13 ○, 14 ○, 15 ○, 16 ○, 17 ○, 18 ○, 19 ○, 20 ○, 21 ○, 22 ○, 23 ○, 24 ○, 25 ○, 26 ○, 27 ○, 28 ○, 29 ○, 30 ○, 31 ○ | Not reported |
| Asteraceae  *Taraxacum officinale* aggr. F.H. Wigg.  KM179 | Kakrunda  (custu giancu, piscianlettu) | Herb | Leaf, Root | Leaf and Root are crushed and are dissolved in water to take for 5-6 days | Flavonoids, carotenoides, sterols, sequiterpenes lactones([Schütz et al., 2006](#_ENREF_227)) | 54 | 0.22 | 0.014 | 1 ○, 2 ○, 3 ○, 4 ○, 5 ○, 6 ○, 7 ○, 8 ○, 9 ○, 10 ○, 11 ○, 12 ○, 13 ○, 14 ○, 15 ○, 16 ○, 17 ●, 18 ○, 19 ○, 20 ○, 21 ○, 22 ○, 23 ○, 24 ○, 25 ○, 26 ○, 27 ○, 28 ○, 29 ○, 30 ●, 31 ● | Plant is used in treatment of (17) jaundice, (30) constipation, (31) purgative, diuretic, laxative,  Kidney and liver diseases |
| Asteraceae  *Vernonia amygdalina* Delile  KM190 | Oriwo | Shrub | Leaf | Extract of Leaf | Steroid glucosides, sesquiterpene lactones, flavonoids ([Adeniyi et al., 2010](#_ENREF_10)) | 54 | 0.22 | 0.005 | 1 ○, 2 ○, 3 ○, 4 ○, 5 ○, 6 ○, 7 ○, 8 ○, 9 ○, 10 ○, 11 ○, 12 ○, 13 ○, 14 ○, 15 ○, 16 ○, 17 ○, 18 ○, 19 ○, 20 ○, 21 ○, 22 ○, 23 ○, 24 ○, 25 ○, 26 ○, 27 ○, 28 ●, 29 ○, 30 ○, 31 ○ | Plant is used in treatment of (28) hypertension |
| Asteraceae  *Gnaphalium affine* D.Don [Tzvelev](http://mpns.kew.org/mpns-portal/plantDetail?plantId=gcc-31294&query=Gnaphalium+affine+&filter=&fuzzy=false&nameType=all&dbs=gcc) KM24 | Jangli dodal | Herb | Leaf | Leaf decoction is for hypertension. | Phenolics glycosides ([Li et al., 2013](#_ENREF_8)) | 54 | 0.22 | 0.006 | 1 ○, 2 ○, 3 ○, 4 ●, 5 ●, 6 ●, 7 ○, 8 ○, 9 ○, 10 ○, 11 ○, 12 ○, 13 ○, 14 ○, 15 ○, 16 ○, 17 ○, 18 ○, 19 ○, 20 ○, 21 ○, 22 ○, 23 ○, 24 ○, 25 ○, 26 ○, 27 ○, 28 ○, 29 ○, 30 ○, 31○ , 32 ○. | Plant is used in treatment of (4) hypertension and cardiac diseases, (5)hypertension and diabetes, (6) hepatic pain, menstruation  pain, conjunctivitis, burnings,  kidney pain, influenza and  headache |
| Berberidaceae  *Berberis vulgaris* L.  KM21 | Zarshak  (Hamida) | Shrub | Seed, Leaf and Fruits | Extract of Seed and Leaf | Terpenoids, lupeol, oleanolic acid, steroids, stigmasterol and stigmasterol glucoside  ([Saied and Begum, 2004](#_ENREF_15)) | 81 | 0.32 | 0.003 | 1 ○, 2 ○, 3 ○, 4 ●, 5 ○, 6 ○, 7 ○, 8 ○, 9 ○, 10 ○, 11 ○, 12 ○, 13 ○, 14 ○, 15 ○, 16 ○, 17 ○, 18 ○, 19 ●, 20●, 21 ○, 22 ○, 23 ○, 24 ○, 25 ○, 26 ○, 27 ○, 28 ○, 29 ○, 30 ○, 31○ | Plant is used in treatment of (4) hypertension and cardiac diseases, (19) gastrointestinal pain and  remove gall stone, (20) have medicinal properties |
| Betulaceae  *Betula alba* [Ehrh.](http://mpns.kew.org/mpns-portal/plantDetail?plantId=21636&query=Betula+pubescens+&filter=&fuzzy=false&nameType=all&dbs=wcs)  KM22 | Bhooj Pathar  Batoula | Shrub | Aerial parts | The extract obtain from the aerial parts of the plant |  | 54 | 0.22 | 0.003 | 1 ○, 2 ○, 3 ○, 4 ●, 5 ○, 6 ○, 7 ○, 8 ○, 9 ○, 10 ○, 11 ○, 12 ○, 13 ○, 14 ○, 15 ○, 16 ○, 17 ○, 18 ○, 19 ○, 20 ○, 21 ○, 22 ○, 23 ○, 24 ○, 25 ○, 26 ○, 27 ○, 28 ○, 29 ○, 30 ○, 31○ | Plant is used in treatment of (4) hypertension and cardiac diseases |
| Bignoniaceae  *Kigelia africana* (Lam.) Benth.  KM105 | Babunah  (Sausage tree) | Tree | Root | Root is boiled in water for about 5 min,  strained and the liquid (about 6 teaspoons) | Alkaloids, saponins, tannins, flavonoids, carbohydrates, sapogenetic glycosides ([Grace et al., 2002](#_ENREF_99)) | 54 | 0.22 | 0.004 | 1 ○, 2 ○, 3 ○, 4 ○, 5 ○, 6 ○, 7 ○, 8 ○, 9 ○, 10 ○, 11 ○, 12 ○, 13 ○, 14 ○, 15 ○, 16 ○, 17 ○, 18 ○, 19 ○, 20 ○, 21 ○, 22 ○, 23 ○, 24 ○, 25 ○, 26 ○, 27 ○, 28 ○, 29 ○, 30 ○, 31○ | Not reported |
| Boraginaceae  *Cordia trichotoma* (Vell.) Arráb. ex Steud.  KM 60 | (Pau-de-morro) | Tree | Flower, Leaf | Leaf and flower decoction | Alkaloids ([Matias et al., 2015](#_ENREF_167)) | 30 | 0.12 | 0.003 | 1 ○, 2 ○, 3 ○, 4 ○, 5 ○, 6 ○, 7 ○, 8 ○, 9 ○, 10 ○, 11 ○, 12 ○, 13 ○, 14 ○, 15 ○, 16 ○, 17 ○, 18 ○, 19 ○, 20 ○, 21 ○, 22 ○, 23 ○, 24 ○, 25 ○, 26 ○, 27 ○, 28 ○, 29 ○, 30 ○, 31○ | Not reported |
| Boraginaceae  *Heliotropium angiospermum* Murray  KM94 | (Crista-de-galo) | Herb | Flower, Leaf, Root | Dried Leaf and Root are used to be eaten and to make a decoction | Flavonoids ([Erosa-Rejón et al., 2009](#_ENREF_84)) | 53 | 0.21 | 0.003 | 1 ○, 2 ○, 3 ○, 4 ○, 5 ○, 6 ○, 7 ○, 8 ○, 9 ○, 10 ○, 11 ○, 12 ○, 13 ○, 14 ○, 15 ○, 16 ○, 17 ○, 18 ○, 19 ○, 20 ○, 21 ○, 22 ○, 23 ○, 24 ○, 25 ○, 26 ○, 27 ○, 28 ○, 29 ○, 30 ○, 31○ | Not reported |
| Boraginaceae  *Heliotropium indicum* L.  KM96 | Siryari  (Indian heliotrope) | Herb | Whole plant | Extract of whole plant | Pyrrolizidine alkaloids ([Souza et al., 2005](#_ENREF_1)) | 54 | 0.22 | 0.007 | 1 ○, 2 ○, 3 ○, 4 ○, 5 ○, 6 ○, 7 ○, 8 ○, 9 ○, 10 ○, 11 ○, 12 ○, 13 ○, 14 ○, 15 ○, 16 ○, 17 ○, 18 ○, 19 ○, 20 ○, 21 ○, 22 ○, 23 ○, 24 ○, 25 ○, 26 ○, 27 ○, 28 ○, 29 ○, 30 ○, 31○ | Not reported |
| Boraginaceae  *Heliotropium lasiocarpum* Fisch.  & C.A.Mey.  KM95 | Chulai  (Hathi Sundi) | Herb | Whole plant | Whole plant decoction | Alkaloids ([Menshikov and Kuzovkov, 1949](#_ENREF_170)) | 135 | 0.54 | 0.001 | 1 ○, 2 ○, 3 ○, 4 ○, 5 ○, 6 ○, 7 ○, 8 ○, 9 ○, 10 ○, 11 ○, 12 ○, 13 ○, 14 ○, 15 ○, 16 ○, 17 ○, 18 ○, 19 ○, 20 ○, 21 ○, 22 ○, 23 ○, 24 ○, 25 ○, 26 ○, 27 ○, 28 ○, 29 ○, 30 ○, 31○ | Not reported |
| Boraginaceae  *Varronia multispicata* (Cham.) Borhidi  KM61 | (Chumbinho, Maria-preta) | Shrub | Flower, Leaf, Aerial part | Infusion of Leaf and Flower | None | 31 | 0.12 | 0.004 | 1 ○, 2 ○, 3 ○, 4 ○, 5 ○, 6 ○, 7 ○, 8 ○, 9 ○, 10 ○, 11 ○, 12 ○, 13 ○, 14 ○, 15 ○, 16 ○, 17 ○, 18 ○, 19 ○, 20 ○, 21 ○, 22 ○, 23 ○, 24 ○, 25 ○, 26 ○, 27 ○, 28 ○, 29 ○, 30 ○, 31 ○ | Not reported |
| Brassicaceae  *Capsella bursa-pastoris* (L.)Medik.  KM33 | Chambraka, Chambraka | Herb | Aerial parts, Seed | The dried aerial parts are crushed with water to make paste and add 1 teaspoon of this paste in milk | Phenolics, and flavonoids ([*Grosso et al., 2011*](#_ENREF_6)*)* | 27 | 0.11 | 0.019 | 1 ○, 2 ○, 3 ○, 4 ○, 5 ○, 6 ○, 7 ○, 8 ○, 9 ○, 10 ○, 11 ○, 12 ○, 13 ○, 14 ○, 15 ○, 16 ●, 17 ●, 18 ○, 19 ○, 20 ○, 21 ○, 22 ○, 23 ○, 24 ●, 25 ○, 26 ○, 27 ○, 28 ○, 29 ○, 30 ○, 31○ , 32 ○. | Plant is used in treatment of (16) gastrointestinal disorder, urinary disorder, wounds healing, cuts, narcotic, tonic, tumor,  anticancer and stimulant, (24) different medicinal uses |
| Brassicaceae  *Eruca sativa* (L.) Cav.  KM77 | Tera mera | Herb | Leaf | Leaf should be collected and extract made | Isothiocyanates, phenolics, carotenoids ([Gulfraz et al., 2011](#_ENREF_105)) | 42 | 0.17 | 0.003 | 1 ○, 2 ○, 3 ○, 4 ○, 5 ○, 6 ○, 7 ○, 8 ○, 9 ○, 10 ○, 11 ○, 12 ○, 13 ○, 14 ○, 15 ○, 16 ○, 17 ○, 18 ○, 19 ○, 20 ○, 21 ○, 22 ○, 23 ○, 24 ○, 25 ○, 26 ○, 27 ○, 28 ○, 29 ○, 30 ○, 31○ | Not reported |
| Brassicaceae  *Lepidium sativum*  L.  KM107 | Halim  (Hebb rchad) | Herb | Seed | Extract of Seed are for 5 days | Essential oisl, geranial, neral, limonene ([Gokavi et al., 2004](#_ENREF_96)) | 61 | 0.24 | 0.003 | 1 ○, 2 ○, 3 ○, 4 ○, 5 ○, 6 ○, 7 ○, 8 ○, 9 ○, 10 ○, 11 ○, 12 ○, 13 ○, 14 ○, 15 ○, 16 ○, 17 ○, 18 ○, 19 ○, 20 ○, 21 ○, 22 ○, 23 ○, 24 ○, 25 ○, 26 ○, 27 ○, 28 ○, 29 ○, 30 ○, 31○ | Not reported |
| Capparaceae  *Capparis spinosa* L.  KM32 | Kabar  (Caper) | Shrub | Seed | Powder of the Seed is given with water 3 times a day | Bitter flavonoid glycosides ([Germano et al., 2002](#_ENREF_91)) | 54 | 0.22 | 0.011 | 1 ○, 2 ○, 3 ●, 4 ●, 5 ●, 6 ○, 7 ○, 8 ○, 9 ○, 10 ○, 11 ○, 12 ○, 13 ○, 14 ○, 15 ○, 16 ○, 17 ○, 18 ○, 19 ●, 20 ○, 21 ○, 22 ○, 23 ●, 24 ○, 25 ○, 26 ○, 27 ○, 28 ○, 29 ○, 30 ○, 31○ | Plant is used in treatment of (3) diabetes, cardiac and renal diseases, (4) diabetes mellitus, hypertension and cardiac diseases, (5) hypertension and diabetes, (19) joint pain, diuretic, dropsy, (23) medicinal use |
| Caprifoliaceae  *Valeriana jatamansi* Jones ex Roxb KM188 | Mash Wali  (Murma) | Herb | Root | Juice of Root. | Essential oils, limonene, cineole, isovaleric acid, caproic acid ([Bhatt et al., 2012](#_ENREF_52)) | 50 | 0.20 | 0.004 | 1 ○, 2 ○, 3 ○, 4 ○, 5 ○, 6 ○, 7 ○, 8 ○, 9 ○, 10 ○, 11 ○, 12 ○, 13 ○, 14 ○, 15 ○, 16 ○, 17 ○, 18 ○, 19 ○, 20 ○, 21 ○, 22 ○, 23 ○, 24 ○, 25 ○, 26 ○, 27 ○, 28 ○, 29 ○, 30 ○, 31○ | Not reported |
| Caprifoliaceae  *Valeriana officinalis* L.  KM189 | Baalchar  (Valeriana, Baymout) | Herb | Root, Leaf | Extract of Leaf and Root are for 2 weeks | Iridoidvalepotriates, volatile essential oil, alkaloids ([Corsi et al., 1984](#_ENREF_68)) | 61 | 0.24 | 0.003 | 1 ○, 2 ○, 3 ○, 4 ○, 5 ○, 6 ○, 7 ○, 8 ○, 9 ○, 10 ○, 11 ○, 12 ○, 13 ○, 14 ○, 15 ○, 16 ○, 17 ○, 18 ○, 19 ○, 20 ○, 21 ○, 22 ○, 23 ○, 24 ○, 25 ○, 26 ○, 27 ○, 28 ○, 29 ○, 30 ○, 31 ○ |  |
| Caricaceae  *Carica papaya* L.  KM37 | Papita  (Papaya, Paw Paw, Okodu) | Tree | Leaf, Fruits, Bark | Aqueous extract of Leaf and bark | Alkaloids , anthroquinones, catachol, flavonoids, phenols, saponins, steriods, triterpenoids, tannins ([Ayoola and Adeyeye, 2010](#_ENREF_43)) | 54 | 0.22 | 0.008 | 1 ○, 2 ○, 3 ○, 4 ○, 5 ○, 6 ○, 7 ○, 8 ○, 9 ○, 10 ○, 11 ○, 12 ○, 13 ○, 14 ○, 15 ○, 16 ○, 17 ○, 18 ○, 19 ○, 20 ○, 21 ○, 22 ○, 23 ○, 24 ○, 25 ○, 26 ○, 27 ○, 28 ●, 29○, 30 ○, 31○ | Plant is used in treatment of (28) hypertension |
| Celastraceae  *Gymnosporia royleana* Wall. ex M.A.Lawson  KM122 | Tokvugri | Shrub | Leaf and Root | Decoctions of Leaf and Root | Terpenoids, traces of phenolic principles ([Hassan et al., 1991](#_ENREF_4)) | 73 | 0.29 | 0.003 | 1 ○, 2 ○, 3 ○, 4 ○, 5 ○, 6 ○, 7 ○, 8 ○, 9 ○, 10 ○, 11 ○, 12 ○, 13 ○, 14 ○, 15 ○, 16 ○, 17 ○, 18 ○, 19 ○, 20 ○, 21 ○, 22 ○, 23 ○, 24 ○, 25 ○, 26 ○, 27 ○, 28 ○, 29 ○, 30 ○, 31○ | Not reported |
| Chenopodiaceae  *Chenopodium ambrosioides* Hance  KM42 | (Wormseed, Mkhinza) | Herb | Leaf, Flower | Juice made from Leaf or Flower is mixed with honey and taken orally | Flavanol glycosides ([Jain et al., 1990](#_ENREF_123)) | 108 | 0.43 | 0.003 | 1 ○, 2 ○, 3 ●, 4 ●, 5 ●, 6 ○, 7 ○, 8 ○, 9 ○, 10 ○, 11 ○, 12 ○, 13 ○, 14 ○, 15 ○, 16 ○, 17 ○, 18 ○, 19 ○, 20 ○, 21 ○, 22 ○, 23 ○, 24 ○, 25 ○, 26 ○, 27 ○, 28 ○, 29 ○, 30 ○, 31○ | Plant is used in treatment of (3) diabetes, cardiac and renal diseases, (4) diabetes mellitus, hypertension and cardiac diseases, (5) hypertension and diabetes |
| Combretaceae  *Terminalia bellerica* (Gaertn.) Roxb.  KM180 | Bahera  (belleric myrobalan, Balela) | Tree | Fruits | Extract of fruits | Polyphenolics ([Pfundstein et al., 2010](#_ENREF_9)) | 108 | 0.43 | 0.003 | 1 ○, 2 ○, 3 ○, 4 ○, 5 ○, 6 ○, 7 ○, 8 ○, 9 ○, 10 ○, 11 ○, 12 ○, 13 ○, 14 ○, 15 ○, 16 ○, 17 ○, 18 ○, 19 ○, 20 ○, 21 ○, 22 ○, 23 ○, 24 ○, 25 ○, 26 ○, 27 ○, 28 ○, 29 ○, 30 ○, 31 ○ | Not reported |
| Commelinaceae  *Callisia gracilis* (Kunth) D.R. Hunt  KM28 | Calcha | Herb | Flower | Dry Flower are dipped in water overnight. |  | 47 | 0.19 | 0.008 | 1 ○, 2 ○, 3 ○, 4 ○, 5 ○, 6 ●, 7 ○, 8 ○, 9 ○, 10 ○, 11 ○, 12 ○, 13 ○, 14 ○, 15 ○, 16 ○, 17○, 18 ○, 19 ○, 20 ○, 21 ○, 22 ○, 23 ○, 24 ○, 25 ○, 26 ○, 27 ○, 28 ○, 29 ○, 30 ○, 31○ , 32 ○. | Plant is used in treatment of (6) hair tonic, high blood  Pressure and rheumatism |
| Commelinaceae  *Commelina benghalensis* L*.*KM58 | Bokhna | Herb | Whole plant | Whole plants are crushed to obtain juice | Terpenoids, saponins, tannins, flavonoids, steroids, phenolic compounds, alkaloids and cardiac glycosides ([Kokilavani et al., 2014](file:///C:\Users\Khafsa\Desktop\Ranier%20skin\frontier\Revision%201\Manuscript.docx#_ENREF_155)) | 54 | 0.22 | 0.001 | 1 ○, 2 ○, 3 ○, 4 ○, 5 ○, 6 ○, 7 ○, 8 ○, 9 ○, 10 ○, 11 ○, 12 ○, 13 ○, 14 ○, 15 ○, 16 ○, 17 ○, 18 ○, 19 ○, 20 ○, 21 ○, 22 ○, 23 ○, 24 ○, 25 ○, 26 ○, 27 ○, 28 ○, 29 ○, 30 ○, 31○ | Not reported |
| Convolvulaceae  *Convolvulus sepium*  (L.) RBr.  KM59 | (Tarbouche la ghrabe) | Herb | Aerial part | Leaf and stems are chopped and cooked alone or with other vegetables | None | 54 | 0.22 | 0.001 | 1 ○, 2 ○, 3 ○, 4 ○, 5 ○, 6 ○, 7 ○, 8 ○, 9 ○, 10 ○,11 ○, 12 ○, 13 ○, 14 ○, 15 ○, 16 ○, 17 ○, 18 ○, 19 ○, 20 ○, 21 ○, 22 ○, 23 ○, 24 ○, 25 ○, 26 ○, 27 ○, 28 ○, 29 ○, 30 ○, 31○ | Not reported |
| Convolvulaceae  *Cuscuta reflexa* Roxb  KM70 | Aftimoon  (Huangtengzi (H), wugencao (HML)) | Herb | Leaf | Dried leaf decoction | Coumarine alpha-amyrin, beta-amyrin, alpha- amyrin acetate, beta-amyrin acetate, oleanolic acetate, oleanolic acid, stigmasterol ([Anis et al., 1999](#_ENREF_35)) | 108 | 0.43 | 0.005 | 1 ○, 2 ○, 3 ○, 4 ○, 5 ○, 6 ○, 7 ○, 8 ●, 9 ○, 10 ○, 11 ○, 12 ○, 13 ○, 14 ○, 15 ○, 16 ●, 17 ○, 18 ○, 19 ○, 20 ○, 21 ○, 22 ○, 23 ○, 24 ○, 25 ○, 26 ○, 27 ○, 28 ○, 29 ○, 30 ●, 31○ | Plant is used in the treatment of (8) hypertension, (16) Dermatological, topical diseases, Cardio-vascular complaints, circulatory diseases and urinary complaints, (30) skin diseases |
| Costaceae  *Costus arabicus* L.  KM63 | (Cana do brejo) | Herb | Leaf | Leaf infusion | Alkaloids, saponins, tannins, anthraquinones, flavonoids ([Paes et al., 2013](#_ENREF_195)) | 33 | 0.13 | 0.003 | 1 ○, 2 ○, 3 ○, 4 ○, 5 ○, 6 ○, 7 ○, 8 ○, 9 ○, 10 ○, 11 ○, 12 ○, 13 ○, 14 ○, 15 ○, 16 ○, 17 ○, 18 ○, 19 ○, 20 ○, 21 ○, 22 ○, 23 ○, 24 ○, 25 ○, 26 ○, 27 ○, 28 ○, 29 ○, 30 ○, 31○ | Not reported |
| Crassulaceae  *Bryophyllum pinnatum* (Lam.) Oken  KM104 | Zakham e Hayat | Herb | Leaf | A decoction of the Leaf | Phenols and triterpenoids and steroids ([Kamboj and Saluja, 2009](#_ENREF_128)) | 59 | 0.24 | 0.003 | 1 ○, 2 ○, 3 ○, 4 ○, 5 ○, 6 ●, 7 ○, 8 ○, 9 ○, 10 ○, 11 ○, 12 ○, 13 ○, 14 ○, 15 ○, 16 ○, 17 ○, 18 ○, 19 ○, 20 ○, 21 ○, 22 ○, 23 ○, 24 ○, 25 ○, 26 ○, 27 ○, 28 ●, 29 ○, 30 ○, 31○ , 32 ○. | Plant is used in treatment of (6) Blood purification”, cancer,  menstruation pain and espanto, (28) hypertension |
| Cucurbitaceae  *Citrullus colocynthis* (L.) Schrad.  KM47 | Toonbi  (Bitter apple, Colocynth, Hdja, Hntel, Khor Tumma) | Herb | Leaf, Aerial part, Fruits, stem | About 15 g of the Leaf is boiled with about 500 ml water, strained and boiled again to reduce to 1/4 of the amount | Alkaloids, glycosides, flavonoids, saponins, tannins, carbohydrate and essential oils. ([Al-Ghaithi et al., 2004](#_ENREF_24)) | 54 | 0.22 | 0.001 | 1 ○, 2 ○, 3 ●, 4 ●, 5 ●, 6 ○, 7 ○, 8 ○, 9 ○, 10 ○, 11 ○, 12 ○, 13 ○, 14 ○, 15 ○, 16 ○, 17 ○, 18 ○, 19 ○, 20 ○, 21 ○, 22 ○, 23 ○, 24 ○, 25 ○, 26 ○, 27 ○, 28 ○, 29 ○, 30 ○, 31○ , 32 ○. | Plant is used in treatment of (3) diabetes, cardiac and renal diseases, (4) diabetes mellitus, hypertension and cardiac diseases, (5) hypertension and diabetes |
| Cucurbitaceae  *Coccinia grandis* (L.) Voigt  KM55 | Kanrori  (Telakucha) | Vine | Leaf | Juice of the root (about 6 teaspoons with curd and molasses) is given twice a day | Glycosides, flavonoids ([Umamaheswari and Chatterjee, 2008](#_ENREF_262)) | 28 | 0.11 | 0.047 | 1 ○, 2 ○, 3 ○, 4 ○, 5 ○, 6 ○, 7 ○, 8 ○, 9 ○, 10 ○, 11 ○, 12 ○, 13 ○, 14 ○, 15 ○, 16 ○, 17 ○, 18 ○, 19 ○, 20 ○, 21 ○, 22 ○, 23 ○, 24 ○, 25 ○, 26 ○, 27 ○, 28 ○, 29 ○, 30 ○, 31○ | Not reported |
| Cucurbitaceae  *Momordica charantia* L.  KM129 | Karela  (Bitter melon, bitter gourd) | Herb | Leaf | Decoction of 100 g Leaf is 1 litre of water and taken orally, 150 cc, three  times/day | Alkaloids, steroids, phenolic compounds, flavonoids tannins, anthraquinones, amino acids ([Britto and Gracelin, 2011](#_ENREF_57)) | 78 | 0.31 | 0.003 | 1 ○, 2 ○, 3 ○, 4 ○, 5 ○, 6 ○, 7 ○, 8 ○, 9 ○, 10 ○, 11 ○, 12 ○, 13 ○, 14 ○, 15 ○, 16 ○, 17 ○, 18 ○, 19 ○, 20 ○, 21 ○, 22 ○, 23 ○, 24 ○, 25 ○, 26 ○, 27 ○, 28 ○, 29 ○, 30 ○, 31○ | Not reported |
| Cyperaceae  *Carex baccans* Nees  KM36 | (Yegaoliang) | Herb | Seed | Seed are boiled in water for about 20 min, strained and take 1 cup 4 times a day | [Resveratrol](https://www.sciencedirect.com/topics/chemistry/resveratrol) and  [phenylpropanoid](https://www.sciencedirect.com/topics/chemistry/phenylpropanoid) [glycosides](https://www.sciencedirect.com/topics/chemistry/glycoside) ([Kumar et al., 2013](#_ENREF_140)) | 27 | 0.11 | 0.034 | 1 ○, 2 ○, 3 ○, 4 ○, 5 ○, 6 ○, 7 ○, 8 ○, 9 ○, 10 ○, 11 ○, 12 ○, 13 ○, 14 ○, 15 ○, 16 ○, 17 ○, 18 ○, 19 ○, 20 ○, 21 ○, 22 ○, 23 ○, 24 ○, 25 ○, 26 ○, 27 ○, 28 ○, 29 ○, 30 ○, 31○ | Not reported |
| Elaeagnaceae  *Hippophae rhamnoides* (L.)  KM98 | (Buckthorn) | Shrub | Bark, Fruit, Stem, Leaf | Decoctions obtain for bark and a leaf | Proanthocyanidins, carotenoids ([Michel et al., 2012](#_ENREF_172)) | 81 | 0.32 | 0.002 | 1 ○, 2 ○, 3 ○, 4 ○, 5 ○, 6 ○, 7 ○, 8 ○, 9 ○, 10 ○, 11 ○, 12 ○, 13 ○, 14 ○, 15 ○, 16 ○, 17 ○, 18 ○, 19 ●, 20 ○, 21 ○, 22 ○, 23 ●, 24 ○, 25 ○, 26 ○, 27 ○, 28 ○, 29 ○, 30 ○, 31○ | The plant is used in the treatment of (19) cough and arthritic pain, (23) medicinal use, (23) medicinally important |
| Equisetaceae  *Equisetum ramosissimum* Desf.  KM76 | Dnab l’khil | Herb | Aerial parts | Leaf and fruit extract | Phenolic compounds, benzoic and cinnamic acid, flavonoids ([Štajner et al., 2009](#_ENREF_245)) | 41 | 0.16 | 0.022 | 1 ○, 2 ○, 3 ○, 4 ○, 5 ○, 6 ○, 7 ○, 8 ○, 9 ○, 10 ○, 11 ○, 12 ○, 13 ○, 14 ○, 15 ○, 16 ○, 17 ○, 18 ○, 19 ○, 20 ○, 21 ○, 22 ○, 23 ○, 24 ○, 25 ○, 26 ○, 27 ○, 28 ○, 29 ○, 30 ○, 31○ | NR |
| Euphorbiaceae  *Euphorbia hirta* L.  KM80 | Dudhi  (Tawa tawa) | Herb | Whole plant | Whole plant juice i | Catechol, flavonoids, phenols, saponins, steriods, triterpenoids, tannins ([Basma et al., 2011](#_ENREF_49)) | 44 | 0.18 | 0.003 | 1 ○, 2 ○, 3 ○, 4 ○, 5 ○, 6 ○, 7 ○, 8 ○, 9 ○, 10 ○, 11 ○, 12 ○, 13 ○, 14 ●, 15 ●, 16 ○, 17 ○, 18 ○, 19 ○, 20 ○, 21 ○, 22 ○, 23 ○, 24 ○, 25 ○, 26 ○, 27 ○, 28 ●, 29 ○, 30 ○, 31○ | Plant is used in the treatment of (14) bronchial infection, asthma and  Warts, (15)  boils, cuts, wounds, asthma, cough, cardiovascular  complaints, asthma, spleen disorders, bronchodilator, antispasmodic,  anti-asthmatic,  expectorant,  anti-catarrhal and  antibacterial activity |
| Fabaceae  *Abrus precatorius* L.  KM01 | Ratti  (Mudepu) | Climber | Leaf | Leaf are shade dried and boil in water to make decoction. Take 2 cups a day | 8-C-glucosylscutelarein 6,7-dimethylether (abrusin) and its 2″-O-apioside ([Markham et al., 1989](#_ENREF_16)) | 27 | 0.11 | 0.009 | 1 ○, 2 ○, 3 ○, 4 ○, 5 ○, 6 ○, 7 ○, 8 ○, 9 ○, 10○, 11 ○, 12 ○, 13 ○, 14 ○, 15 ○, 16 ○, 17 ○, 18 ○, 19 ○, 20 ○, 21 ○, 22 ○, 23 ○, 24 ○, 25 ○, 26 ○, 27 ○, 28 ●, 29 ○, 30 ○, 31○ | Plant for treatment of (28) hypertension. |
| Fabaceae  *Butea monosperma* (Lam.) Kuntze.  KM26 | Plaspara  (Dhak) | Tree | Bark | Bark of the plant is dipped in water to made infusion and used on empty stomach | Starch, tannins, flavonoid and, glycosides ([Sahu and Padhy, 2013](#_ENREF_217)) Isobutrin  and butrin ([Krolikiewicz-Renimel et al., 2013](#_ENREF_139)) | 27 | 0.11 | 0.044 | 1 ○, 2 ○, 3 ○, 4 ○, 5 ○, 6 ○, 7 ○, 8 ○, 9 ○, 10 ○, 11 ○, 12 ○, 13 ○, 14 ○, 15 ○, 16 ○, 17 ○, 18 ○, 19 ○, 20 ○, 21 ○, 22 ○, 23 ○, 24 ○, 25 ○, 26 ○, 27 ○, 28 ○, 29 ○, 30 ○, 31○ , 32 ○. | Not reported |
| Fabaceae  *Erythrina suberosa Roxb* KM78 | Guato | Tree | Stem | Stem decoction is used to made infusion | Erysubins C–F, four isoflavonoids ([Tanaka et al., 2001](#_ENREF_251)) | 108 | 0.43 | 0.001 | 1 ○, 2 ○, 3 ○, 4 ○, 5 ○, 6 ○, 7 ○, 8 ○, 9 ○, 10 ○, 11 ○, 12 ○, 13 ○, 14 ○, 15 ○, 16 ○, 17 ○, 18 ○, 19 ○, 20 ○, 21 ○, 22 ○, 23 ○, 24 ○, 25 ○, 26 ○, 27 ○, 28 ○, 29 ○, 30 ○, 31○ | NR |
| Fabaceae  *Glycyrrhiza glabra* L.  KM90 | Mulathi  (Mulethi, Arq’souss) | Herb | Root &Aerial parts | Root extraction | Catechol, flavonoids, phenols, saponins, steriods, triterpenoids, tannins ([Varsha et al., 2013](#_ENREF_265)) | 54 | 0.22 | 0.003 | 1 ○, 2 ○, 3 ●, 4 ●, 5 ○, 6 ○, 7 ○, 8 ○, 9 ○, 10 ○, 11 ○, 12 ○, 13 ○, 14 ○, 15 ○, 16 ○, 17 ○, 18 ○, 19 ○, 20 ○, 21 ○, 22 ○, 23 ○, 24 ○, 25 ○, 26 ○, 27 ○, 28 ○, 29 ○, 30 ○, 31○ | The plant is used in the treatment of (3) diabetes, cardiac and renal diseases, (4) diabetes mellitus, hypertension and cardiac diseases |
| Fabaceae  *Melilotus officinalis* (L.) Pall.  KM125 | Aspang  (Cornilla real) | Herb | Stem | Stem infusion | Alkaloids, flavonoids and saponin ([Mojab et al., 2010](#_ENREF_175)) | 75 | 0.30 | 0.003 | 1 ○, 2 ○, 3 ○, 4 ○, 5 ○, 6 ○, 7 ○, 8 ○, 9 ○, 10 ○, 11 ○, 12 ○, 13 ○, 14 ○, 15 ○, 16 ○, 17 ○, 18 ○, 19 ○, 20 ○, 21 ○, 22 ○, 23 ○, 24 ○, 25 ○, 26 ○, 27 ○, 28 ○, 29 ○, 30 ○, 31○ |  |
| Fabaceae  *Senna tora* (L.) Roxb.  KM169 | Panwar  (Stinking cassia) | Herb | Leaf | Flower and stem are grinded and are dissolved in water and taken for 5-6 days | Alkaloids, saponins ([Ogunkunle and Ladejobi, 2006](#_ENREF_188)) | 108 | 0.43 | 0.012 | 1 ○, 2 ○, 3 ○, 4 ○, 5 ○, 6 ○, 7 ○, 8 ○, 9 ○, 10 ○, 11 ○, 12 ○, 13 ○, 14 ○, 15 ○, 16 ○, 17 ○, 18 ○, 19 ○, 20 ○, 21 ○, 22 ○, 23 ○, 24 ○, 25 ○, 26 ○, 27 ○, 28 ○, 29 ○, 30 ○, 31 ○ | Not reported |
| Fabaceae  *Tamarindus indica* L.  KM177 | Imli  (Tamarind) | Tree | Root | A decoction is prepared from 50g boiled Root in 1 liter water for 30 min and taken internally, 150 cc, four times/day until the condition improves. | Saponins, alkaloids and glycosides ([Abukakar et al., 2008](#_ENREF_8)) | 27 | 0.11 | 0.013 | 1 ○, 2 ○, 3 ○, 4 ○, 5 ○, 6 ●, 7 ○, 8 ○, 9 ○, 10 ○, 11 ○, 12 ○, 13 ○, 14 ○, 15 ○, 16 ○, 17 ○, 18 ○, 19 ○, 20 ○, 21 ○, 22 ○, 23 ○, 24 ○, 25 ○, 26 ○, 27 ○, 28 ○, 29 ○, 30 ○, 31 ○ | Plant is used in treatment of (6) Stomach pain and hepatic pain |
| Fabaceae  *Trigonella foenum-graecum* L.  KM186 | Methi  (Halba) | Herb | Seed | Extract of Seed | Phenol, flavanol, amino acids, alkaloides, steroides, tannin, polysaccarides, pectin, volatile oil ([Ahmadiani et al., 2001](#_ENREF_19)) | 114 | 0.46 | 0.001 | 1 ○, 2 ○, 3 ●, 4 ○, 5 ●, 6 ○,, 7 ○, 8 ○, 9 ○, 10 ○, 11 ○, 12 ○, 13 ○, 14 ○, 15 ○, 16 ○, 17 ○, 18 ○, 19 ○, 20 ○, 21 ○, 22 ○, 23 ○, 24 ○, 25 ○, 26 ○, 27 ○, 28 ○, 29 ○, 30 ○, 31 ○ | Plant is used in treatment of (3) diabetes, cardiac and renal diseases |
| Fabaceae  *Bauhinia forficata* Link  KM18 | (Pata de vaca) | Tree | Leaf | 1 cup of a standard leaf infusion is taken three times daily with meals | Phenols and flavonoids ([da Silva et al., 2000](#_ENREF_69)) | 108 | 0.43 | 0.004 | 1 ○, 2 ○, 3 ○, 4 ○, 5 ○, 6 ○, 7 ○, 8 ○, 9 ○, 10 ○, 11 ○, 12 ○, 13 ○, 14 ○, 15 ○, 16 ○, 17 ○, 18 ○, 19 ○, 20 ○, 21 ○, 22 ○, 23 ○, 24 ○, 25 ○, 26 ○, 27 ○, 28 ○, 29 ○, 30 ○, 31○ | Not reported |
| Fabaceae  *Prosopis juliflora* (SW.)DC KM 30 | Kikri | Shrub | Whole plant, aerial parts | Oil of the plant is used to cure muscles pain | Tannins, phenolics, flavonoids, alkaloids, terpenes and steroids([Singh, 2012](#_ENREF_238)) | 65 | 0.26 | 0.003 | 1 ○, 2 ○, 3 ○, 4 ○, 5 ○, 6 ○, 7 ○, 8 ○, 9 ○, 10 ○, 11 ○, 12 ○, 13 ○, 14 ○, 15 ○, 16 ○, 17 ○, 18 ○, 19 ○, 20 ○, 21 ○, 22 ○, 23 ○, 24 ○, 25 ○, 26 ○, 27 ○, 28 ○, 29 ○, 30 ○, 31○ , 32 ○. | Not reported |
| Fagaceae  *Quercus ilex* L.  KM156 | (Tabor oak) | Tree | Stem, Bark, Fruits | Decoction of stem and bark is taken twice a day in the morning | Polyphenols ([Gharzouli et al., 1999](#_ENREF_92)) | 97 | 0.39 | 0.012 | 1 ○, 2 ○, 3 ○, 4 ○, 5 ○, 6 ○, 7 ○, 8 ○, 9 ○, 10 ○, 11 ○, 12 ○, 13 ○, 14 ○, 15 ○, 16 ○, 17 ○, 18 ○, 19 ○, 20 ○, 21 ○, 22 ○, 23 ○, 24 ○, 25 ○, 26 ○, 27 ○, 28 ○, 29 ○, 30 ○, 31 ○ | Not reported |
| Gentianaceae  *Gentiana purpurea* L..  KM88 | Charaita  (Trumpet gentian) | Herb | Leaf | Extract of Leaf | Alkaloids ([Singh, 2008](#_ENREF_237)) | 49 | 0.20 | 0.004 | 1 ○, 2 ○, 3 ○, 4 ○, 5 ○, 6 ○, 7 ○, 8 ○, 9 ○, 10 ○, 11 ○, 12 ○, 13 ○, 14 ○, 15 ○, 16 ○, 17 ○, 18 ○, 19 ○, 20 ○, 21 ○, 22 ○, 23 ○, 24 ○, 25 ○, 26 ○, 27 ○, 28 ○, 29 ○, 30 ○, 31○ | Not reported |
| Geraniaceae  *Geranium wallichianum* D. Don ex Sweet  KM89 | Ratan jog | Herb | Root | Root dried overnight and then extracted | Flavonoids ([Ismail et al., 2012](#_ENREF_121)) | 50 | 0.20 | 0.004 | 1 ○, 2 ○, 3 ○, 4 ○, 5 ○, 6 ○, 7 ○, 8 ○, 9 ○, 10 ○, 11 ○, 12 ○, 13 ○, 14 ○, 15 ○, 16●, 17 ○, 18 ●, 19 ○, 20 ●, 21 ○, 22 ○, 23 ○, 24 ○, 25 ○, 26 ○, 27 ○, 28 ○, 29 ○, 30 ○, 31● | The plant is used in the treatment of (16) respiratory illness. fever, headache, analgesic, genital and sexual diseases, (18) backache, mouth ulceration and  chronic diarrhea, (20) medicinal value, (31) kidney diseases, cough and fever |
| Juglandaceae  *Juglans regia* L.  KM103 | Akhrot  (Walnut) | Tree | Leaf, stems, Aerial parts | Decoction and extract of Leaf and Root | Phenols ([Oliveira et al., 2008](#_ENREF_22)) | 58 | 0.23 | 0.003 | 1 ○, 2 ○, 3 ●, 4 ○, 5 ●, 6 ○, 7 ○, 8 ○, 9 ○, 10 ○, 11 ○, 12 ●, 13 ○, 14 ○, 15 ○, 16 ●, 17 ○, 18 ○, 19 ○, 20 ○, 21 ○, 22 ○, 23 ○, 24 ○, 25 ○, 26 ○, 27 ○, 28 ○, 29 ○, 30 ●, 31 ● | Plant is used in the treatment of (3) diabetes, cardiac and renal diseases, (5) hypertension and diabetes, (12) wound healing, (16) dermatological, topical diseases, dental problem, wounds, cuts, narcotic, tonic, tumor,  anticancer and stimulant, (30) Eczema, fruit, timber, (31) Brain tonic, antiseptic and  toothbrush. |
| Lamiaceae  *Ajuga integrifolia* Buch-Ham. ex D. Don KM04 | Butey | Herb | Leaf | Fresh Leaf are used to make decoction |  | 28 | 0.11 | 0.010 | 1 ○, 2 ○, 3 ○, 4 ○, 5 ○, 6 ○, 7 ○, 8 ○, 9 ○, 10 ○, 11 ○, 12 ○, 13 ○, 14 ○, 15 ○, 16 ○, 17 ○, 18 ○, 19 ○, 20 ○, 21 ○, 22 ○, 23 ○, 24 ○, 25 ○, 26 ○, 27 ○, 28 ○, 29 ○, 30 ○, 31○ | Not Reported |
| Lamiaceae  *Calamintha hydaspidis* (Falc. ex Benth.) Hedge  KM27 | (Abies) | Herb | Stem | Infusion made from stem |  | 54 | 0.22 | 0.003 | 1 ○, 2 ○, 3 ○, 4 ○, 5 ○, 6 ○, 7 ○, 8 ○, 9 ○, 10 ○, 11 ○, 12 ○, 13 ○, 14 ○, 15 ○, 16 ○, 17 ○, 18 ○, 19 ○, 20 ○, 21 ○, 22 ○, 23 ○, 24 ○, 25 ○, 26 ○, 27 ○, 28 ○, 29 ○, 30 ○, 31○ , 32 ○. | Not reported |
| Lamiaceae*Volkameria inermis*LGaertn. KM53 | Guldamdam  (Indian privet, glory bower) | Shrub | Leaf | Three Leaf are powdered and taken with warm water | Flavonoid ([Krishnadhas et al.](#_ENREF_137)) | 26 | 0.10 | 0.004 | 1 ○, 2 ○, 3 ○, 4 ○, 5 ○, 6 ○, 7 ○, 8 ○, 9 ○, 10 ○, 11 ○, 12 ○, 13 ○, 14 ○, 15 ○, 16 ○, 17 ○, 18 ○, 19 ○, 20 ○, 21 ○, 22 ○, 23 ○, 24 ○, 25 ○, 26 ○, 27 ○, 28 ○, 29 ○, 30 ○, 31○ | Not reported |
| Lamiaceae  *Lallemantia royleana* (Benth.) Benth.  KM110 | Tukhme Balanga  (Salvia Seed) | Herb | Seed | Seed are swallowing orally with water | Carbohydrates, fiber, oil, proteins, tannins ([Sharifi‐Rad et al., 2015](#_ENREF_229)) | 64 | 0.26 | 0.003 | 1 ○, 2 ○, 3 ○, 4 ○, 5 ○, 6 ○, 7 ○, 8 ○, 9 ○, 10 ○, 11 ○, 12 ○, 13 ○, 14 ○, 15 ○, 16 ○, 17 ○, 18 ○, 19 ○, 20 ○, 21 ○, 22 ○, 23 ○, 24 ○, 25 ○, 26 ○, 27○, 28 ○, 29 ○, 30 ○, 31○ | Not reported |
| Lamiaceae  *Lavandula angustifolia* Mill  KM112 | Khushbudar  (English lavender) | Shrub | Leaf | Decoction of Leaf and Root | Linalool, linalyl acetate and some other mono- and sesquiterpenes, flavonoids like luteolin, triterpenoids like ursolic acid and coumarins like umbelliferone and coumarin ([Hajhashemi et al., 2003](#_ENREF_8)) | 66 | 0.26 | 0.005 | 1 ○, 2 ○, 3 ○, 4 ○, 5 ●, 6 ○, 7 ○, 8 ○, 9 ○, 10 ○, 11 ○, 12 ○, 13 ○, 14 ○, 15 ○, 16 ○, 17 ○, 18 ○, 19 ○, 20 ○, 21 ○, 22 ○, 23 ○, 24○, 25 ○, 26 ○, 27 ○, 28 ○, 29 ○, 30 ○, 31○ | Plant is used in treatment of (5) hypertension and diabetes |
| Lamiaceae  *Marrubium radiatum* Delile ex Benth.  KM118 |  | Shrub | Aerial parts | Decoction of aerial parts | None | 71 | 0.28 | 0.002 | 1 ○, 2 ○, 3 ○, 4 ○, 5 ○, 6 ○, 7 ○, 8 ○, 9 ○, 10 ○, 11 ○, 12 ○, 13 ○, 14 ○, 15 ○, 16 ○, 17 ○, 18 ○, 19 ○, 20 ○, 21 ○, 22 ○, 23 ○, 24 ○, 25 ○, 26 ○, 27 ○, 28 ○, 29 ○, 30 ○, 31○ | Not reported |
| Lamiaceae  *Marrubium vulgare* L.  KM119 | Lehsan Koi  (Horehound, Merriwa, Merriwta) | Shrub | Leaf, Aerial part | Extract of Leaf | (E)-caffeoyl-l-malic acid,  glycosidic derivatives: acteoside ,forsythoside B 3, arenarioside,  ballotetroside ([Sahpaz et al., 2002](#_ENREF_215)) | 243 | 0.97 | 0.020 | 1 ○, 2 ○, 3 ○, 4 ●, 5 ●, 6 ○, 7 ○, 8 ○, 9 ○, 10 ○, 11 ○, 12 ○, 13 ○, 14 ○, 15 ○, 16 ○, 17 ○, 18 ○, 19 ○, 20 ○, 21 ○, 22 ○, 23 ○, 24 ○, 25 ○, 26 ○, 27 ○, 28 ○, 29 ○, 30 ○, 31○, | Plant is used in treatment of (4) diabetes, hypertension and cardiac diseases, (5) hypertension and diabetes |
| Lamiaceae  *Melissa officinalis* L.  KM126 | Zarnab  (Melissa, Erva cidreira) | Herb | Leaf | Infusion is made by soaking 30g of Leaf in water and drinks 2 cups daily | Essencial oils (containing citral and  citronellal monoterpenes), flavonoids and rosmarinic,  caffeic and chlorogenic acids ([Nascimento et al., 2000](#_ENREF_183)) | 81 | 0.32 | 0.003 | 1 ○, 2 ○, 3 ○, 4 ○, 5 ○, 6 ○, 7 ○, 8 ○, 9 ○, 10 ○, 11 ○, 12 ○, 13 ○, 14 ○, 15 ○, 16 ○, 17 ○, 18 ○, 19 ○, 20 ○, 21 ○, 22 ○, 23 ○, 24 ○, 25 ○, 26 ○, 27 ○, 28 ○, 29 ○, 30 ○, 31○ |  |
| Lamiaceae  *Mentha aquatica* L.  KM127 | Podina  (Ehohwa) | Herb | Leaf | Leaf are used to make teas | Flavonoids ([Voirin et al., 1999](#_ENREF_31)) | 76 | 0.30 | 0.003 | 1 ○, 2 ○, 3 ○, 4 ○, 5 ○, 6 ○, 7 ○, 8 ○, 9 ○, 10 ○, 11 ○, 12 ○, 13 ○, 14 ○, 15 ○, 16 ○, 17 ○, 18 ○, 19 ○, 20 ○, 21 ○, 22 ○, 23 ○, 24 ○, 25 ○, 26 ○, 27 ○, 28 ○, 29 ○, 30 ○, 31○ | Not reported |
| Lamiaceae  *Mentha longifolia* (L.) Huds.  KM128 | Jangli Podina  (Whadan, Filil) | Herb | Leaf, Flower | Extract made from the Leaf | Pulegone,isomenthone,1,8-cineole,borneol, and piperitenoneoxide ([Idrissi and Fkih-Tetouani, 2001](#_ENREF_120)) | 77 | 0.31 | 0.003 | 1 ○, 2 ○, 3 ○, 4 ○, 5 ○, 6 ○, 7 ○, 8 ●, 9 ○, 10 ○, 11 ○, 12 ○, 13 ○, 14 ○, 15 ○, 16 ○, 17 ○, 18 ●, 19 ○, 20 ○, 21 ○, 22 ○, 23 ●, 24 ○, 25 ○, 26 ○, 27 ○, 28 ○, 29 ○, 30●, 31● | Plant is used in treatment of (8) hypertension, (18) Joint pain and digestive disorders, (23) veterinary, fed to calves  and yak with skin problems and  'sun-burns', (30) stimulant, aromatic and carminative,  Vegetables, (31) diarrhea and vomiting. |
| Lamiaceae  *Ocimum basilicum* L.  KM137 | Jangli Tulsi  (Albahaca) | Herb | Leaf | An infusion of 20 g Leaf in 1 liter water is prepared and taken three times/day as needed until improvement occurs | Volatile oils, unusual flavones, sterols, triterpenes, sesquiterpene alcohols of copane series, rosmarinic acid ([Daniel et al., 2011](#_ENREF_72)) | 84 | 0.34 | 0.003 | 1 ○, 2 ○, 3 ●, 4 ○ , 5 ○ , 6 ●, 7 ○, 8 ●, 9 ○, 10 ○, 11 ○, 12 ○, 13 ○, 14 ○, 15 ○, 16 ○, 17 ○, 18 ○, 19 ○, 20 ○, 21 ○, 22 ○, 23 ○, 24 ○, 25 ○, 26 ○, 27 ○, 28 ○, 29 ○, 30 ●, 31○ | Plant is used in treatment of (3) diabetes, cardiac and renal diseases, (6) stomach pain, fever, gastritis,  influenza, high blood  pressure, internal infections and  relaxant, (8) hypertension, (30) demulcent and diuretic. |
| Lamiaceae  *Origanum vulgare* L.  KM139 |  | Shrub | Leaf | Extract made from the Leaf. | 1,7-Dioxaspiro[5,5]undec-2-ene, 2,4-Dihydroxy-2,5-dimethyl-39(2H)-furan-3-one, 2,4-Difurobenzene and 1-benzyloxy, α-D-Glucopyranoside, O-α- Glucopyranosyl ([Al-Tameme et al., 2015](#_ENREF_25)) | 85 | 0.34 | 0.004 | 1 ○, 2 ○, 3 ○, 4 ●, 5 ○, 6 ○, 7 ○, 8 ●, 9 ○, 10 ○, 11 ○, 12 ○, 13○, 14 ●, 15 ○, 16 ●, 17 ●, 18 ●, 19 ○, 20 ○, 21 ○, 22 ○, 23 ○, 24 ○, 25 ○, 26 ○, 27 ○, 28 ○, 29 ○, 30 ○, 31○ | Plant is used in treatment of (4) diabetes and cardiac diseases, (hypertension, (8) hypertension (16) Diuretic and against toothache and earache, (17) toothache and mouth gums, (18) skin infections, Sexual weakness,digestive disorders, Intestinal pain and urinary disorder |
| Lamiaceae  *Prunella vulgaris* L.  KM153 | Kalveuth  (Xiagudcao) | Herb | Whole plant | Decoction of Leaf is used with *Aloe vera* | Alkaloids, saponins, phenolics, tannins ([Rasool et al., 2010](#_ENREF_207)) | 94 | 0.38 | 0.003 | 1 ○, 2 ○, 3 ○, 4 ○, 5 ○, 6 ○ , 7 ○, 8 ○, 9 ○, 10 ○, 11 ○, 12 ○, 13 ○, 14 ○, 15 ○, 16 ○, 17 ○, 18 ○, 19 ●, 20 ○, 21 ○, 22 ○, 23 ○, 24 ○, 25 ○, 26 ○, 27 ○, 28 ○, 29 ○, 30 ○, 31 ○ | Plant is used in treatment of (6) Stomachache, sore throat, diuretic, tonic |
| Lamiaceae  *Salvia rosmarinus* Schleid KM157 | Akeel Kohistani  (Azir) | Herb | Aerial parts | Extract of the aerial parts are given at evening time | Essential oils, carnosol and betulinic acid ([Machado et al., 2013](#_ENREF_1)) | 54 | 0.22 | 0.003 | 1 ○, 2 ○, 3 ●, 4 ●, 5 ●, 6 ●, 7 ○, 8 ○, 9 ○, 10 ○, 11 ○, 12 ○, 13 ○, 14 ○, 15 ○, 16 ○, 17 ○, 18 ○, 19 ○, 20 ○, 21 ○, 22 ○, 23 ○, 24 ○, 25 ○, 26 ○, 27 ○, 28 ○, 29 ○, 30 ○, 31 ○ | Plant is used in treatment of (3) diabetes, cardiac and renal diseases, (4) diabetes, hypertension and cardiac, (5) hypertension and diabetes, (6) espanto, headache,  stomach pain, anemia and  internal infections |
| Lamiaceae  *Rydingia persica* (Burm.f.) Scheen & V.A.Albert  KM140 | Golder | Shrub | Leaf, Flower | Leaf decoction | Diterpenoids ([Ayatollahi et al., 2015](#_ENREF_42)) | 86 | 0.34 | 0.001 | 1 ○, 2 ○, 3 ○, 4 ○, 5 ○, 6 ○, 7 ○, 8 ○, 9 ○, 10 ○, 11 ○, 12 ○, 13 ○, 14 ○, 15 ○, 16 ○, 17 ○, 18 ○, 19 ○, 20 ○, 21 ○, 22 ○, 23 ○, 24 ○, 25 ○, 26 ○, 27 ○, 28 ○, 29 ○, 30 ○, 31 ○ | Not reported |
| Lamiaceae  *Salvia fruticosa* Mill.  KM168 | Saloia  (Greek sage) | Herb | Flower, Stems | An infusion of 50 g in 1 litre is prepared  and taken orally, 150 cc, 1/3 times/day until improvement occurs | Volatile compounds ([Kamatou et al., 2008](#_ENREF_127)) | 103 | 0.41 | 0.003 | 1 ○, 2 ○, 3 ○, 4 ○, 5 ○, 6 ○, 7 ○, 8 ○, 9 ○, 10 ○, 11 ○, 12 ○, 13 ○, 14 ○, 15 ○, 16 ○, 17 ○, 18 ○, 19 ○, 20 ○, 21 ○, 22 ○, 23 ○, 24 ○, 25 ○, 26 ○, 27 ○, 28 ○, 29 ○, 30 ○, 31 ○ | Not reported |
| Lamiaceae  *Salvia officinalis* L.  KM167 | Sepakass  (Salva, Salmia) | Herb | Flower, Leaf | Decoction of Leaf is taken 3 times daily | Flavonoids and phenolic glycosides ([Lu and Foo, 2000](#_ENREF_155)) | 102 | 0.41 | 0.001 | 1 ○, 2 ○, 3 ○, 4 ●, 5 ●, 6 ○, 7 ○, 8 ○, 9 ○, 10 ○, 11 ○, 12 ○, 13 ○, 14 ○, 15 ○, 16 ○, 17 ○, 18 ○, 19 ○, 20 ○, 21 ○, 22 ○, 23 ○, 24 ○, 25 ○, 26 ○, 27 ○, 28 ○, 29 ○, 30 ○, 31 ○ | Plant is used in treatment of (4)diabetes, hypertension and cardiac diseases, (5) hypertension and diabetes |
| Lamiaceae  *Teucrium rogleanom* L.  KM181 | Kalpurak | Shrub | Flower, Stem | A decoction of the root is boiled to a gelatinous  Mass. Take 3 cup of hot water add 2 spoons of grinded plant mix well and use trice a day. | Iridoids, flavonoids, diterpenoids ([Menichini et al., 2009](#_ENREF_169)) | 109 | 0.44 | 0.003 | 1 ○, 2 ○, 3 ○, 4 ○, 5 ○, 6 ○, 7 ○, 8 ○, 9 ○, 10 ○, 11 ○, 12 ○, 13 ○, 14 ○, 15 ○, 16 ○, 17 ○, 18 ○, 19 ○, 20 ○, 21 ○, 22 ○, 23 ○, 24 ○, 25 ○, 26 ○, 27 ○, 28 ○, 29 ○, 30 ○, 31 ○ | Not reported |
| Lamiaceae  *Thymus linearis* Benth.  KM182 | Hasha  (Azoukni, Zaatar) | Shrub | Leaf | Aqueous extract | Thymol, carvacrol, thymyl acetate, β-caryophyllene , carvacrol, o-cymene, α-terpineol , α-pinene and β-caryophyllene ([Hussain et al., 2013](#_ENREF_117)) | 110 | 0.44 | 0.004 | 1 ○, 2 ○, 3 ○, 4 ○, 5 ○, 6 ○, 7 ○, 8 ○, 9 ○, 10 ○, 11 ○, 12 ○, 13 ○, 14 ○, 15 ○, 16 ●, 17 ○, 18 ○, 19 ○, 20 ○, 21 ○, 22 ○, 23 ○, 24 ○, 25 ○, 26 ○, 27 ○, 28 ○, 29 ○, 30 ○, 31 ● | Plant is used in treatment of (16) pain and fever, (31) Carminative, antispasmodic, cough and cold |
| Linaceae  *Linum usitatissimum* L.  KM114 | Alsi  (Zarriat al Kettan) | Shrub | Seed | Dried powdered Leaf are mixed with water and juice is extracted and taken | Phytoestrogens (lignans), flavonoids, amino acids, minerals ([Chung et al., 2005](#_ENREF_67)) | 81 | 0.32 | 0.002 | 1 ○, 2 ○, 3 ●, 4 ●, 5 ○, 6 ○, 7 ○, 8 ○, 9 ○, 10 ○, 11 ○, 12 ○, 13 ○, 14 ○, 15 ○, 16 ○, 17 ○, 18 ○, 19 ○, 20 ○, 21 ○, 22 ○, 23 ○, 24 ○, 25 ○, 26 ○, 27 ○, 28 ○, 29 ○, 30 ○, 31○ | Plant is used in the treatment of (3) diabetes, cardiac and renal diseases, (4) diabetes, hypertension and cardiac diseases |
| Lythraceae  *Lawsonia inermis* L.  KM113 | Mehndi  (Hanna) | Tree | Leaf | Leaf juice | Carbohydrates, proteins,  flavonoids, tannins and phenolic compounds, alkaloids, terpenoids, quinones, coumarins, xanthones and fatty acids ([Chaudhary et al., 2010](#_ENREF_63)) | 67 | 0.27 | 0.003 | 1 ○, 2 ○, 3 ○, 4 ●, 5 ○, 6 ○, 7 ○, 8 ○, 9 ○, 10 ○, 11 ○, 12 ○, 13 ○, 14 ○, 15 ○, 16 ○, 17 ○, 18 ○, 19 ○, 20 ○, 21 ○, 22 ○, 23 ○, 24 ○, 25 ○, 26 ○, 27 ○, 28 ○, 29 ○, 30 ○, 31○ | Plant is used in treatment of (4) diabetes, hypertension and cardiac diseases |
| Lythraceae  *Punica granatum* L.  KM155 | Anar  (Pomegranate) | Tree | Fruit | Juice of the fruits is given for 1 week | Phenolic acids, flavonoids, tannins ([Mena et al., 2011](#_ENREF_168)) | 96 | 0.38 | 0.001 | 1 ○, 2 ○, 3 ●, 4 ●, 5 ●, 6 ○, 7 ○, 8 ○, 9 ○, 10 ○, 11 ○, 12 ○, 13 ○, 14 ○, 15 ○, 16 ○, 17 ○, 18 ○, 19 ○, 20 ○, 21 ○, 22 ○, 23 ○, 24 ○, 25 ○, 26 ○, 27 ○, 28 ○, 29 ○, 30 ●, 31 ○ | Plant is used in treatment of (3) diabetes, cardiac and renal, (4) diabetes, hypertension and cardiac, (30) antipyretic, and fruit |
| Malvaceae  *Cola acuminata* (P. Beauv.) Schott, Endl.  KM57 | Kola  (Colatier) | Tree | Fruits | Fruits are taken orally | Flavonoids ([Sonibare et al., 2009](#_ENREF_242)) | 29 | 0.12 | 0.011 | 1 ○, 2 ○, 3 ○, 4 ○, 5 ○, 6 ○, 7 ○, 8 ○, 9 ○, 10 ○, 11 ○, 12 ○, 13 ○, 14 ○, 15 ○, 16 ○, 17 ○, 18 ○, 19 ○, 20 ○, 21 ○, 22 ○, 23 ○, 24 ○, 25 ○, 26 ○, 27 ○, 28 ○, 29 ○, 30 ○, 31○ | Not reported |
| Malvaceae  *Hibiscus sabdariffa* L.  KM97 | Karkader  (Chaii torsh, Sorrel) | Herb | Flower | Infusion of Flower | Anthocyanins, Hibiscus protocatechuic acid, glycinebetaine ([Ali et al., 2005](#_ENREF_27)) | 55 | 0.22 | 0.004 | 1 ○, 2 ○, 3 ○,, 4 ○,, 5 ○, 6 ○, 7 ○, 8 ○, 9 ●, 10 ○, 11 ○, 12 ○, 13 ○, 14 ○, 15 ○, 16 ○, 17 ○, 18 ○, 19 ○, 20 ○, 21 ○, 22 ○, 23 ○, 24 ○, 25 ●, 26 ○, 27 ○, 28 ●, 29 ○, 30 ○, 31○ | The plant is used in the treatment of (9) hypertenison, (25) hypertension, (28) hypertension |
| Malvaceae  *Malva sylvestris* L.  KM116 | Khubazi  Narba | Herb | Root | Decoction of Root is used 2 cups a day | Phenols, flavonoids, carotenoids, tocopherols, 8-hydroxyflavonoid glucuronides ([Billeter et al., 1991](#_ENREF_5)) | 69 | 0.28 | 0.003 | 1 ○, 2 ○, 3 ○, 4 ○, 5 ○, 6 ○, 7 ○, 8 ○, 9 ○, 10 ○, 11 ○, 12 ○, 13 ○, 14 ○, 15 ○, 16 ○, 17 ○, 18 ○, 19 ○, 20 ○, 21 ○, 22 ○, 23 ○, 24 ○, 25 ○, 26 ○, 27 ○, 28 ○, 29 ○, 30 ○, 31○ | Not reported |
| Meliaceae  *Melia azedarach* L.  KM123 | Bakayan  (Bakanra) | Tree | Leaf, Seed | Leaf decoction are for 2-3 days | Alkaloids, Tannins, Saponins, Phenols ([Ahmed et al., 2012](#_ENREF_20)) | 54 | 0.22 | 0.006 | 1 ○, 2 ○, 3 ○, 4 ○, 5 ○, 6 ○, 7 ○, 8 ○, 9 ○, 10 ○, 11 ○, 12 ○, 13 ○, 14 ○, 15 ○, 16 ●, 17 ○, 18 ●, 19 ○, 20 ○, 21 ○, 22 ○, 23 ○, 24 ○, 25 ○, 26 ○, 27 ○, 28 ○, 29 ○, ○, 30 ●, 31○ | Plant is used in treatment of (16) gastrointestinal diseases, respiratory illness, fever, headache and analgesic, (18) Leprosy and Urinary disorders, (30) anthelmintic, fodder, fuel wood, TSR and  timber |
| Menispermaceae  *Cissampelos capensis* L. f.  KM45 | Dawidjie | Shrub | Leaf | Decoction is prepared from two tsps of dried Leaf boiled in 1 liter water for 20 min, five tsps are taken daily until improvement occurs | Alkaloids and flavonoids ([Babajide et al., 2015](#_ENREF_4)) | 22 | 0.09 | 0.003 | 1 ○, 2 ○, 3 ○, 4 ○, 5 ○, 6 ○, 7 ○, 8 ○, 9 ○, 10 ○, 11 ○, 12 ○, 13 ○, 14 ○, 15 ○, 16 ○, 17 ○, 18 ○, 19 ○, 20 ○, 21 ○, 22 ○, 23 ○, 24 ○, 25 ○, 26 ○, 27 ○, 28 ○, 29 ○, 30 ○, 31○ | Not reported |
| Menispermaceae  *Tinospora sinensis (*Lour.) Merr[.](http://mpns.kew.org/mpns-portal/plantDetail?plantId=516871&query=Tinospora+malabarica+%28Lam.%29+&filter=&fuzzy=false&nameType=all&dbs=wcsCmp)  KM183 | Amrita  (Amruthaballi) | Herb | Leaf | Leaf paste is mixed with milk and drink one glass a day | Cycloeucalenone, sterols, β-sitosterol and stigmasterol ([Parveen et al., 2016](#_ENREF_198)) | 111 | 0.44 | 0.003 | 1 ○, 2 ○, 3 ○, 4 ○, 5 ○, 6 ○, 7 ○, 8 ○, 9 ○, 10 ○, 11 ○, 12 ○, 13 ○, 14 ○, 15 ○, 16 ○, 17 ○, 18 ○, 19 ○, 20 ○, 21 ○, 22 ○, 23 ○, 24 ○, 25 ○, 26 ○, 27 ○, 28 ○, 29 ○, 30 ●, 31 ○ | Plant is used in treatment of (30) fever and ornamental |
| Moraceae  *Ficus palmata* [Forssk.](http://mpns.kew.org/mpns-portal/plantDetail?plantId=811619&query=Ficus+palmate+&filter=&fuzzy=true&nameType=all&dbs=wcsCmp)  KM81 | Pakodo | Shrub | Leaf | Leaf extract | Furanocoumarin derivatives, rutin, germanicol acetate, vanillic acid and psoralenoside methyl ether ([Alqasoumi et al., 2014](#_ENREF_31)) | 43 | 0.17 | 0.006 | 1 ○, 2 ○, 3 ○, 4 ○, 5 ○, 6 ○, 7 ○, 8 ○, 9 ○, 10 ○, 11 ○, 12 ○, 13 ○, 14 ○, 15 ○, 16 ○, 17 ○, 18 ○, 19 ○, 20 ○, 21 ○, 22 ○, 23 ○, 24 ○, 25 ○, 26 ○, 27 ○, 28 ○, 29 ○, 30 ○, 31○ | Not reported |
| Moraceae  *Ficus carica* L.  KM82 | Pakodo  (Brahma’s banyan) | Shrub | Fruits | Fruits are eaten | Alkaloids ([Ahmad and Beg, 2001](#_ENREF_14)) | 45 | 0.18 | 0.003 | 1 ○, 2 ○, 3 ●, 4 ○, 5 ●, 6 ●, 7 ○, 8 ○, 9 ○, 10 ○, 11 ○, 12 ○, 13 ○, 14 ○, 15 ○, 16 ○, 17 ○, 18 ○, 19 ○, 20 ○, 21 ○, 22 ○, 23 ○, 24 ○, 25 ○, 26 ○, 27 ○, 28 ○, 29 ○, 30 ○, 31○ | The plant is used in the treatment of (3) diabetes, cardiac and renal diseases, (5) diabetes, hypertension, (6) bronchitis, menstruation, pain and sedative |
| Moraceae  *Morus alba* L.  KM131 | Toot  (Pawi bush) | Tree | Fruits | Fruit is eaten raw to avoid hypertension | Alkaloids, tannins, steroids, flavonoids, saponin ([Wang et al., 2013](#_ENREF_269)) | 80 | 0.32 | 0.003 | 1 ○, 2 ○, 3 ○, 4 ○, 5 ○, 6 ○, 7 ○, 8 ○, 9 ○, 10 ○, 11 ○, 12 ○, 13 ○, 14 ○, 15 ○, 16 ●, 17 ○, 18 ○, 19 ○, 20 ○, 21 ○, 22 ○, 23 ○, 24 ○, 25 ○, 26○, 27 ○, 28 ○, 29 ○, 30 ●, 31● | Plant is used in treatment of (16) Constipation , respiratory illness and cough , (30) laxative, fodder, fuel wood, fruit, TSR and  Timber, (31) edible, laxative, purgative,  Emollient and fodder, thatching,  basket making. |
| Moringaceae  *Moringa oleifera* Lam.  KM130 | Sohanjna  (Horseradish tree, Nugge) | Tree | Stem, Leaf | Leaf and Flower extract are given to patients with hypertension.  Juice of plant (about 4 teaspoons) is given | Ascorbic acid, ﬂavonoids, phenolics, carotenoids ([Anwar et al., 2007](#_ENREF_37)) | 79 | 0.32 | 0.002 | 1 ○, 2 ○, 3 ○, 4 ○, 5 ○, 6 ○, 7 ○, 8 ○, 9 ○, 10 ○, 11 ○, 12 ○, 13 ○, 14 ○, 15 ○, 16 ○, 17 ○, 18 ○, 19 ○, 20 ○, 21 ○, 22 ○, 23 ○, 24 ○, 25 ○, 26 ○, 27 ○, 28 ○, 29 ○, 30 ○, 31○ | Not reported |
| Musaceae  *Musa paradisiaca* L.  KM132 | Kela | Tree | Stem , Flower, Fruit | Fruits Flower and stems decoctions | Steryl glycosides and acyl steryl glycosides ([Ghosal, 1985](#_ENREF_93)) | 54 | 0.22 | 0.008 | 1 ○, 2 ○, 3 ○, 4 ○, 5 ○, 6 ○, 7 ○, 8 ○, 9 ○, 10 ○, 11 ○, 12 ○, 13 ○, 14 ○, 15 ○, 16 ○, 17 ○, 18 ○, 19 ○, 20 ○, 21 ○, 22 ○, 23 ○, 24 ○, 25 ○, 26 ○, 27 ○, 28 ○, 29 ○, 30 ○, 31○ | Not reported |
| Myristicaceae  *Myristica fragrans* Houtt  KM133 | Jaifal  (Nutmeg) | Tree | Stems |  | Myristicin, safrole, myristic acid, trimyristicin, eugenol, elimicin, meso-dihydroguaiaretic acid ([Mary Helen et al., 2012](#_ENREF_165)) | 81 | 0.32 | 0.001 | 1 ○, 2 ○, 3 ○, 4 ○, 5 ○, 6 ○, 7 ○, 8 ○, 9 ○, 10 ○, 11 ○, 12 ○, 13 ○, 14 ○, 15 ○, 16 ○, 17 ○, 18 ○, 19 ○, 20 ○, 21 ○, 22 ○, 23 ○, 24 ○, 25 ○, 26 ○, 27 ○, 28 ○, 29 ○, 30 ○, 31○ | Not reported |
| Myrtaceae  *Eucalyptus globulus* Labill.  KM75 | Safaida  (Kalitous) | Tree | Leaf, Fruits | Leaf extract | Stigmasterol, 3-O-b-D-glucopyranoside, ursolic acid, a-amyrin, a-amyrin acetate, 4,5,7-trimethoxykaempferol ([Pereira et al., 2005](#_ENREF_200)) | 40 | 0.16 | 0.004 | 1 ○, 2 ○, 3 ●, 4 ●, 5 ●, 6 ○, 7 ○, 8 ●, 9 ○, 10 ○, 11 ○, 12 ○, 13 ○, 14 ○, 15 ○, 16 ○, 17 ○, 18 ○, 19 ○, 20 ○, 21 ○, 22 ○, 23 ○, 24 ○, 25 ○, 26 ○, 27 ○, 28 ○, 29 ○, 30 ○, 31○ | Plant is used in the treatment of (3) diabetes, cardiac and renal diseases, (4) diabetes mellitus, hypertension and cardiac diseases. (5) hypertension and diabetes, (8) hypertension |
| Myrtaceae  *Myrtus communis* L.  KM134 | Mushak Mehandi  (Myrtle, Raihane) | Shrub | Seed | Seed decoctions and infusions | Taninis, flavanoids alkaloids, protein, saponins ([Sumbul et al., 2011](#_ENREF_247)) | 82 | 0.33 | 0.003 | 1 ○, 2 ○, 3 ●, 4 ●, 5 ●, 6 ○, 7 ○, 8 ○, 9 ○, 10 ○, 11 ○, 12 ○, 13 ○, 14 ○, 15 ○, 16 ○, 17 ○, 18 ○, 19 ○, 20 ○, 21 ○, 22 ○, 23 ○, 24 ○, 25 ○, 26 ○, 27 ○, 28 ○, 29 ○, 30 ○, 31○, 11 ○, 12 ○, 13 ○, 14 ○, 15 ○, 16 ○, 17 ○, 18 ○, 19 ○, 20 ○, 21 ○, 22○, 23 ○, 24 ○, 25 ○, 26 ○, 27 ○, 28 ○, 29 ○, 30 ○, 31○ | Plant is used in treatment of (3) diabetes, cardiac and renal diseases, (4) diabetes, hypertension and cardiac diseases, (5) hypertension and diabetes |
| Myrtaceae  *Psidium guajava* L.  KM154 | Amrood  (Apple guava) | Tree | Fruit | Extract obtained from the fruit. | Flavonoids, glycosides, alkaloids, steroids ([Metwally et al., 2010](#_ENREF_171)) | 95 | 0.38 | 0.004 | 1 ○, 2 ○, 3 ○, 4 ○, 5 ○, 6 ●, 7 ○, 8 ○, 9 ○, 10 ○, 11 ○, 12 ○, 13 ○, 14 ○, 15 ○, 16 ○, 17 ○, 18 ○, 19 ○, 20 ○, 21 ○, 22 ○, 23 ○, 24 ○, 25 ○, 26 ○, 27 ○, 28 ●, 29 ○, 30 ○, 31 ○ | Plant is used in treatment of (6) diarrhoea, (28) hypertension |
| Myrtaceae  *Syzygium aromaticum* (L.) Merr. & L.M. Perry  KM175 | Loung  (Qrunful) | Tree | Leaf, Fruits | Ethanolic extract of Leaf | Favonoide triglycosides ([Pundir et al., 2010](#_ENREF_205)) | 135 | 0.54 | 0.000 | 1 ○, 2 ○, 3 ○, 4 ○, 5 ○, 6 ○, 7 ○, 8 ○, 9 ○, 10 ○, 11 ○, 12 ○, 13 ○, 14 ○, 15 ○, 16 ○, 17 ○, 18 ○, 19 ○, 20 ○, 21 ○, 22 ○, 23 ○, 24 ○, 25 ○, 26 ○, 27 ○, 28 ●, 29 ○, 30 ○, 31 ○ | Plant is used in treatment of (28) hypertension |
| Nitrariaceae  *Peganum harmala* L.  KM141 | Asfand, Harmal  (Harmel) | Herb | Seed | Seed extract | Alkaloids, flavonoids and anthraquinones ([Bukhari et al., 2008](#_ENREF_9)) | 87 | 0.35 | 0.003 | 1 ○, 2 ○, 3 ●, 4 ●, 5 ●, 6 ○, 7 ○, 8 ○, 9 ○, 10 ○, 11 ○, 12 ○, 13 ○, 14 ○, 15 ○, 16 ○, 17 ○, 18 ○, 19 ○, 20 ○, 21 ○, 22 ○, 23 ●, 24 ○, 25 ○, 26 ○, 27 ○, 28 ○, 29 ○, 30 ○, 31○ | Plant is used in the treatment of (3) diabetes, cardiac and renal diseases, (4) diabetes, hypertension and cardiac diseases (5) hypertension and diabetes |
| Oleaceae  *Olea europaea subsp. cuspidata*(Wall. & G.Don) Cif.  KM138 | Kauh | Tree | Fruit | Fruit oil or leaf decoction are taken orally | Glycosides, secoiridoid, flavonoids, poly-unsaturated fatty acids ([Hayes et al., 2011](#_ENREF_113)) | 54 | 0.22 | 0.008 | 1 ○, 2 ○, 3 ○, 4 ○, 5 ○, 6 ○, 7 ○, 8 ●, 9 ○, 10 ○, 11 ○, 12 ○, 13 ○, 14 ○, 15 ○, 16 ●, 17 ○, 18 ○, 19 ○, 20 ○, 21 ○, 22 ○, 23 ○, 24 ○, 25 ○, 26 ○, 27 ○, 28 ○, 29 ○, 30 ○, 31○ | Plant is used in treatment of (2) antiatherosclerotic (4) diabetes and cardiac diseases, (5) diabetes, (8) hypertension ,(16) anthelmintic,  diuretic; juice , skin diseases, headache and  mouth rash |
| Papaveraceae  *Argemone mexicana* L.  KM13 | Bharband  (Mexican poppy) | Herb | Whole plant | Whole plant is crushed to make paste. This paste is mixed with water and taken orally | Alkaloids,  terpenoids, flavonoids, phenolic, long-chain aliphatic  compounds, ([Abubacker and Ramanathan, 2012](#_ENREF_7)) | 55 | 0.22 | 0.006 | 1 ○, 2 ○, 3 ○, 4 ○, 5 ○, 6 ●, 7 ○, 8 ○, 9 ○, 10 ○, 11 ○, 12 ○, 13 ○, 14 ○, 15 ○, 16 ○, 17 ○, 18 ○, 19 ○, 20 ○, 21 ○, 22 ○, 23 ○, 24 ○, 25 ○, 26 ○, 27 ○, 28 ○, 29 ○, 30 ○, 31○ , 32 ○. | Plant is used in treatment of (6) Internal infections and  antiparasite |
| Papaveraceae  *Fumaria officinalis* L.  KM87 | Shahtara  (Dimnjaˇca, El Bakoula) | Herb | Aerial parts | Dried Leaf decoction and juice | Alkaloids ([Sengul et al., 2009](#_ENREF_228)) | 48 | 0.19 | 0.004 | 1 ○, 2 ○, 3 ○, 4 ●, 5 ○, 6 ○, 7 ○, 8 ○, 9 ○, 10 ○, 11 ○, 12 ○, 13 ○, 14 ○, 15 ○, 16 ○, 17 ○, 18 ○, 19 ○, 20 ○, 21 ○, 22 ○, 23 ○, 24 ○, 25 ○, 26 ○, 27 ○, 28 ○, 29 ○, 30 ○, 31○ | The plant is used in the treatment of (4) diabetes, hypertension and cardiac diseases |
| Papaveraceae  *Papaver somniferum* L.  KM142 | Khashkhash  (Kokinar) | Herb | Seed, Fruit | Decoction made from the Seeds | Morphine, codeine ([Dittbrenner et al., 2007](#_ENREF_78)) | 54 | 0.22 | 0.008 | 1 ○, 2 ○, 3 ○, 4 ●, 5 ○, 6 ○, 7 ○, 8 ○, 9 ○, 10 ○, 11 ●, 12 ○, 13 ○, 14 ○, 15 ○, 16 ●, 17 ○, 18 ○, 19 ○, 20 ○, 21 ○, 22 ○, 23 ○, 24 ○, 25 ○, 26 ○, 27 ○, 28 ○, 29 ○, 30 ○, 31○ | Plant is used in treatment of (4) diabetes, hypertension and cardiac diseases, (11) use for various medicinal purposes, (16) cough and fever |
| Passifloraceae  *Passiflora ligularis* Juss.  KM143 | (Granadilla) | Tree | Leaf, Flower | 100ml of infusion is recommended for 4-5 days | None | 88 | 0.35 | 0.003 | 1 ○, 2 ○, 3 ○, 4 ○, 5 ○, 6 ●, 7 ○, 8 ○, 9 ○, 10 ○, 11 ○, 12 ○, 13 ○, 14 ○, 15 ○, 16 ○, 17 ○, 18 ○, 19 ○, 20 ○, 21 ○, 22 ○, 23 ○, 24 ○, 25 ○, 26 ○, 27 ○, 28 ○, 29 ○, 30 ○, 31○ | The plant is for the treatment of (6) inflammation, hepatic pain,  high cholesterol, scurvy and high  blood pressure |
| Pedaliaceae  *Sesamum indicum* L.  KM170 | Mugose | Herb | Leaf | Juice of root (about 4 teaspoons 3 times a day) | Saponins, cardenolides, flavonoids, polyphenols ([Park et al., 2010](#_ENREF_197)) | 104 | 0.42 | 0.003 | 1 ○, 2 ○, 3 ○, 4 ○, 5 ○, 6 ○, 7 ○, 8 ○, 9 ○, 10 ○. 11 ○, 12 ○, 13 ○, 14 ○, 15 ○, 16 ○, 17 ○, 18 ○, 19 ○, 20 ○, 21 ○, 22 ○, 23 ○, 24 ○, 25 ○, 26 ○, 27 ○, 28 ○, 29 ○, 30 ○, 31 ○ | Not reported |
| Phyllanthaceae  *Phyllanthus emblica* L.  KM146 | Amla  (Iu’gam’iab) | Tree | Leaf | Powder of Leaf and fruits is taken 2 times a day after every meal | Alkaloids, oil, fat, glyceroids, carbohydrates, phenolics, tannins, lignin, saponins, flavonoids,terpenoids ([Dhale and Mogle, 2011](#_ENREF_74)) | 135 | 0.54 | 0.008 | 1 ○, 2 ○, 3 ○, 4 ○, 5 ○, 6 ○, 7 ○, 8 ○, 9 ○, 10 ○, 11 ○, 12 ○, 13 ○, 14 ○, 15 ○, 16 ○, 17 ○, 18 ○, 19 ○, 20 ○, 21 ○, 22 ○, 23 ○, 24 ○, 25 ○, 26 ○, 27 ○, 28 ○, 29 ○, 30 ○, 31○ | Not reported |
| Phytolaccaceae  *Phytolacca dioica* (Moq.) H. Walter  KM147 | Humilla | Tree | Leaf | Infusion of Root is taken 3 times daily | Alkaloid, tannin, saponins, phenolics, lectins, flavonoids ([Ashafa et al., 2010](#_ENREF_41)) | 135 | 0.54 | 0.001 | 1 ○, 2 ○, 3 ○, 4 ○, 5 ○, 6 ○, 7 ○, 8 ○, 9 ○, 10 ○, 11 ○, 12 ○, 13 ○, 14 ○, 15 ○, 16 ○, 17 ○, 18 ○, 19 ○, 20 ○, 21 ○, 22 ○, 23 ○, 24 ○, 25 ○, 26 ○, 27 ○, 28 ○, 29 ○, 30 ○, 31○ | Not Reported |
| Pinaceae  *Pinus wallichiana* A.B. Jacks.  KM149 |  | Tree | Leaf | Few grams of Seed and fruits are used to make extract | Monoterpene alcohols terpinen and α-terpineol ([Satyal et al., 2013](#_ENREF_225)) | 54 | 0.22 | 0.009 | 1 ○, 2 ○, 3 ○, 4 ○, 5 ○, 6 ○, 7 ○, 8 ○, 9 ○, 10 ○. 11 ○, 12 ○, 13 ○, 14 ○, 15 ○, 16 ●, 17 ○, 18 ○, 19 ○, 20 ○, 21 ○, 22 ○, 23 ○, 24 ○, 25 ○, 26 ○, 27 ○, 28 ○, 29 ○, 30 ●, 31● | Plant is used in the treatment of (16) musculoskeletal disorder, (30) fuel wood, TSR and timber, (31) fuel wood, furniture and healing  agent. |
| Piperaceae  *Piper nigrum* L.  KM150 | Kali Mirch  (Black pepper) | Herb | Leaf | The aqueous extract of Leaf is taken twice a day | Piperine,amides, piperidines, pyrrolidines, trace amounts of safrole ([Siddiqui et al., 2005](#_ENREF_235)) | 91 | 0.36 | 0.003 | 1 ○, 2 ○, 3 ○, 4 ○, 5 ○, 6○, 7 ○, 8 ○, 9 ○, 10 ○, 11 ○, 12 ○, 13 ○, 14 ○, 15 ○, 16 ○, 17 ○, 18 ○, 19 ○, 20 ○, 21 ○, 22 ○, 23 ○, 24 ○, 25 ○, 26 ○, 27 ○, 28 ○, 29 ○, 30 ○, 31○ | Not reported |
| Piperaceae  *Piper umbellatum* L.  KM151 | (Cow foot leaf) | Herb | Leaf | The extract of Leaf is taken for a week | Alkaloid, saponin, tannin, Inulin ([Ahmad and Tawan, 2002](#_ENREF_13)) | 92 | 0.37 | 0.003 | 1 ○, 2 ○, 3 ○, 4 ○, 5 ○, 6 ○, 7 ○, 8 ○, 9 ○, 10 ○, 11 ○, 12 ○, 13 ○, 14 ○, 15 ○, 16 ○, 17 ○, 18 ○, 19 ○, 20 ○, 21 ○, 22 ○, 23 ○, 24 ○, 25 ○, 26 ○, 27 ○, 28 ○, 29 ○, 30 ○, 31○ | Not reported |
| Plantaginaceae  *Plantago major* L.  KM152 | Bartang  (Bag yapragı) | Herb | Leaf | Decoction of the Leaf is given for 2 week | Polysaccharides, lipids, caffeic acid derivatives, flavonoids, iridoid glycosides and terpenoids ([Samuelsen, 2000](#_ENREF_221)) | 93 | 0.37 | 0.003 | 1 ○, 2 ○, 3 ●, 4 ○, 5 ○, 6 ○, 7 ○, 8 ○, 9 ○, 10 ○, 11 ○,12 ○, 13 ○, 14 ○, 15 ○, 16 ●, 17 ○, 18 ○, 19 ○, 20 ○, 21 ○, 22 ○, 23 ○, 24 ○, 25 ○, 26 ○, 27 ○, 28 ○, 29 ○, 30 ●, 31 ○ | Plant is used in treatment of (3) diabetes, cardiac and renal diseases, (16) bedsores and  Candidiasis, (30) diarrhea and dysentery |
| Poaceae  *Cymbopogon citratus* (Dc.) Stapf  KM72 | Agan Ghas  (Citronelle, Lemon grass) | Herb | Leaf | Leaf should be dried at shade and then decoction made | Essential oil ([Asaolu et al., 2009](#_ENREF_39)) | 135 | 0.54 | 0.002 | 1 ○, 2 ○, 3 ○, 4 ○, 5 ○, 6 ●, 7 ○, 8 ○, 9 ○, 10 ○, 11 ○, 12 ○, 13 ○, 14 ○, 15 ○, 16 ○, 17 ○, 18 ○, 19 ○, 20 ○, 21 ○, 22 ○, 23 ○, 24 ○, 25 ○, 26 ○, 27 ○, 28 ○, 29 ○, 30 ○, 31○ | Plant is used in the treatment of (6) Gastritis, relaxant, stomach  Pain and diarrhea |
| Poaceae  *Hordeum vulgare* L.  KM100 | Jao  (Gebs, York) | Herb | Seed | Juice obtaied from Seed | Phenolic compounds, including flavonols, phenolic acids, procyanidins ([Anwar et al., 2010](#_ENREF_36)) | 67 | 0.27 | 0.002 | 1 ○, 2 ○, 3 ○, 4 ○, 5 ○, 6 ○, 7 ○, 8 ○, 9 ○, 10 ○, 11 ○, 12 ○, 13 ○, 14 ○, 15 ○, 16 ○, 17 ○, 18 ○, 19 ○, 20 ○, 21 ○, 22 ○, 23 ●, 24 ○, 25 ○, 26 ○, 27 ○, 28 ○, 29 ○, 30 ○, 31○ | The plant is used in the treatment of (23) medicinal important in area |
| Poaceae  [*Cenchrus americanu*s (L.) Morrone](http://mpns.kew.org/mpns-portal/plantDetail?plantId=462670&query=Pennisetum+typhoides+&filter=&fuzzy=false&nameType=all&dbs=wcs) KM144 | Bajra  (Illan) | Herb | Leaf | A decoction is prepared by boiling 50 g of Leaf in 1 litre water for 15 min and taken orally, one cup/day until improvement occurs. | None | 89 | 0.36 | 0.007 | 1 ○, 2 ○, 3 ○, 4 ●, 5 ●, 6 ○, 7 ○, 8 ○, 9 ○, 10 ○, 11 ○, 12 ○, 13 ○, 14 ○, 15 ○, 16 ○, 17 ○, 18 ○, 19 ○, 20 ○, 21 ○, 22 ○, 23 ○, 24 ○, 25○, 26○, 27 ○, 28 ○, 29 ○, 30 ○, 31○ | Plant is used in the treatment of (4) diabetes, hypertension and cardiac diseases (5) hypertension and diabetes |
| Poaceae  *Saccharum officinarum* L.  KM166 | Gana  (Caña, Sugar cane) | Herb | Leaf | Leaf is boiled in water | Triterpenoids, flavonoids, phenolics, phytosterols ([Bhore et al., 2012](#_ENREF_53)) | 101 | 0.40 | 0.003 | 1 ○, 2 ○, 3 ○, 4 ○, 5 ○, 6 ○, 7 ○, 8 ○, 9 ○, 10 ○, 11 ○, 12 ○, 13 ○, 14 ○, 15 ○, 16 ○, 17 ○, 18 ○, 19 ○, 20 ○, 21 ○, 22 ○, 23 ○, 24 ○, 25 ○, 26 ○, 27 ○, 28 ○, 29 ○, 30 ○, 31 ○ | Not reported |
| Poaceae  *Sorghum halepense* (L.) Pers.  KM173 | Baru  (Edra) | Herb | Aerial parts | Aqueous Extract obtained for aerial part of the plant | Flavonolignans tricin-4'-O-(threo-β-guaiacylglyceryl) ether and tricin-4'-O-(erythro-β-guaiacylglyceryl) ether ([Huang et al., 2010](#_ENREF_116)) | 107 | 0.43 | 0.005 | 1 ○, 2 ○, 3 ○, 4 ●, 5 ○, 6 ○, 7 ○, 8 ○, 9 ○, 10 ○, 11 ○, 12 ○, 13 ○, 14 ○, 15 ○, 16 ○, 17 ○, 18 ○, 19 ○, 20 ○, 21 ○, 22 ○, 23 ○, 24 ○, 25 ○, 26 ○, 27 ○, 28 ○, 29 ○, 30 ○, 31 ○ | Plant is used in treatment of (4) diabetes, hypertension and cardiac diseases |
| Poaceae  *Zea mays* L.  KM194 | Makai  (Milho, Maize, Corn) | Shrub | Seed, Leaf, Fruits | Dried Seed are dipped whole night in water | Flavonoids, saponin, tannins,  phlobatannins, phenols, alkaloids and cardiac glycosides ([Solihah et al., 2012](#_ENREF_240)) | 65 | 0.26 | 0.004 | 1 ○, 2 ○, 3 ●, 4 ○, 5 ●, 6 ●, 7 ○, 8 ○, 9 ○, 10 ○, 11 ○, 12 ○, 13 ○, 14 ○, 15 ○, 16 ○, 17 ○, 18 ○, 19 ○, 20 ○, 21 ○, 22 ○, 23 ○, 24 ○, 25 ○, 26 ○, 27 ○, 28 ●, 29 ○, 30 ○, 31 ○ | Plant is used in treatment of (3) diabetes, cardiac and renal diseases, (5) hypertension and diabetes, (6) Stomach pain and dermatitis, (28) hypertension |
| Polygonaceae  *Rheum australe*D.Don KM161 | Rewand Chini  (Syrian rhubarb) | Herb | Root | Infusion of the Root is given for 2 weeks | Anthraquinone glycoside ([Gupta et al., 2014](#_ENREF_107)) | 135 | 0.54 | 0.002 | 1 ○, 2 ○, 3 ○, 4 ○, 5 ○, 6 ○, 7 ○, 8 ○, 9 ○, 10 ○, 11 ○, 12 ○, 13 ○, 14 ○, 15 ○, 16 ○, 17 ○, 18 ○, 19 ○, 20 ○, 21 ○, 22 ○, 23 ○, 24 ○, 25 ○, 26 ○, 27 ○, 28 ○, 29 ○, 30 ○, 31 ○ | Not reported |
| Polygonaceae  *Rumex* *hastatus* D. Don KM165 | Mekmeko, Meqmoqo | Shrub | Leaf, Root | An infusion is prepared from 30 g Leaf in 1 litre water and taken orally, 2/3times/day until the condition improves | Resveratrol, rumexoside, torachrysone‐8‐yl *β*‐D‐glucopyranoside, rutin, nepodin, and orientaloside ([Sahreen et al., 2011](#_ENREF_216)) | 81 | 0.32 | 0.005 | 1 ○, 2 ○, 3 ○, 4 ○, 5 ○, 6 ○, 7 ○, 8 ●, 9 ○, 10 ○, 11 ○, 12 ○, 13 ○, 14 ○, 15 ○, 16 ○, 17 ○, 18 ○, 19 ○, 20 ○, 21 ○, 22 ○, 23 ○, 24 ○, 25 ○, 26 ○, 27 ○, 28 ○, 29 ○, 30 ●, 31 ● | Plant is used in treatment of (8) hypertension, (30) diuretic and stomachic, (31) vegetable and refrigerant enhance  digestion |
| Ranunculaceae  *Nigella sativa* L.  KM136 | Kalonji  (Siadon) | Herb | Seed | 0.5 tsp seed oil in one cup water is taken orally, two times/day | Alkaloids, organic acids, tannins, resins, toxic glucoside, metarbin, bitter principles, glycosidal saponins ([Javed et al., 2012](#_ENREF_125)) | 83 | 0.33 | 0.001 | 1 ○, 2 ○, 3 ○, 4 ●, 5 ●, 6 ○, 7 ○, 8 ○, 9 ○, 10 ○, 11 ○, 12 ○, 13 ○, 14 ○, 15 ○, 16 ○, 17 ○, 18 ○, 19 ○, 20 ○, 21 ○, 22 ○, 23 ○, 24 ○, 25 ○, 26 ○, 27 ○, 28 ○, 29 ○, 30 ○, 31○ | Plant is used in treatment of (4) diabetes, hypertension and cardiac diseases, (5) hypertension and diabetes |
| Rhamnaceae  *Ziziphus mauritiana* Lam.  KM196 | Anab  (Bair) | Tree | Fruit, Leaf | Decoction of Leaf, Juice extracted from fruits | Phenolic compounds, tannins, saponins ([Dahiru et al., 2005](#_ENREF_70))/ | 67 | 0.27 | 0.003 | 1 ○, 2 ○, 3 ○, 4 ○, 5 ○, 6 ○, 7 ○, 8 ○, 9 ○, 10 ○, 11 ○, 12 ○, 13 ○, 14 ○, 15 ○, 16 ○, 17 ○, 18 ○, 19 ○, 20 ○, 21 ○, 22 ○, 23 ○, 24 ○, 25 ○, 26 ○, 27 ○, 28 ○, 29 ○, 30 ○, 31 ○ | Not reported |
| Rosaceae  *Crataegus aronia* L.KM64 | Zaeroor | Shrub | Fruit, Flower | Flower and fruit decoction | Phenolics ([Çalişkan et al., 2012](#_ENREF_60)) | 34 | 0.14 | 0.071 | 1 ○, 2 ○, 3 ○, 4 ○, 5 ○, 6 ○, 7 ○, 8 ○, 9 ○, 10 ○, 11 ○, 12 ○, 13 ○, 14 ○, 15 ○, 16 ○, 17 ○, 18 ○, 19 ○, 20 ○, 21 ○, 22 ○, 23 ○, 24 ○, 25 ○, 26 ○, 27 ○, 28 ○, 29 ○, 30 ○, 31○ | Not reported |
| Rosaceae  *Crataegus rhipidophylla* Gand.KM65 | Kateli jari  (Adam) | Tree | Leaf | Leaf decoction. | None | 35 | 0.14 | 0.048 | 1 ○, 2 ○, 3 ○, 4 ○, 5 ○, 6 ○, 7 ○, 8 ○, 9 ○, 10 ○, 11 ○, 12 ○, 13 ○, 14 ○, 15 ○, 16 ○, 17 ○, 18 ○, 19 ○, 20 ○, 21 ○, 22 ○, 23 ○, 24○, 25 ○, 26 ○, 27 ○, 28 ○, 29 ○, 30 ●, 31● | Plant is used in the treatment of (30) fodder, fuel wood, fruit and fencing, (31) edible and heart tonic. |
| Rosaceae  *Crataegus songarica* K.Koch  KM66 | Dakh | Shrub | Flower, Fruits | Dried Flower and fruits. | Alkaloids, flavonoids, triterpenoids, saponin, steroids and tannins ([Mohan and Middha](#_ENREF_174)) | 162 | 0.65 | 0.001 | 1 ○, 2 ○, 3 ○, 4 ○, 5 ○, 6 ○, 7 ○, 8 ○, 9 ○, 10 ○, 11 ○, 12 ○, 13 ○, 14 ○, 15 ○, 16 ○, 17 ○, 18 ○, 19 ○, 20 ○, 21 ○, 22 ○, 23 ○, 24 ○, 25 ○, 26 ○, 27 ○, 28 ○, 29 ○, 30 ○, 31○ | NR |
| Rosaceae  *Crataegus pinnatifida* Bunge  KM67 | (Chinese Hawthorn) | Tree | Aerial part | Aerial parts decoction | Poly phenolics ([Tassell et al., 2010](#_ENREF_254)) | 135 | 0.54 | 0.002 | 1 ○, 2 ○, 3 ○, 4 ○, 5 ○, 6 ○, 7 ○, 8 ○, 9 ○, 10 ○, 11 ○, 12 ○, 13 ○, 14 ○, 15 ○, 16 ○, 17 ○, 18 ○, 19 ○, 20 ○, 21 ○, 22 ○, 23 ○, 24 ○, 25 ○, 26 ○, 27 ○, 28 ○, 29 ○, 30 ○, 31○ | Not reported |
| Rosaceae  *Cydonia oblonga* Mill. KM71 | Behi  (Marmeleiro) | Tree | Leaf | Leaf are dried under shade and boiled in water to obtain decoction | Phenolic compounds, l-ascorbic acid ([Fattouch et al., 2007](#_ENREF_87)) | 37 | 0.15 | 0.003 | 1 ○, 2 ○, 3 ○, 4 ○, 5 ●, 6 ○, 7 ○, 8 ○, 9 ○, 10 ○, 11 ○, 12 ○, 13 ○, 14 ○, 15 ○, 16 ○, 17 ○, 18 ○, 19 ○, 20 ○, 21 ○, 22 ○, 23 ○, 24 ○, 25 ○, 26 ○, 27 ○, 28 ○, 29 ○, 30 ○, 31○ | Plant is used in the treatment of (5) hypertension and diabetes |
| Rosaceae  *Filipendula ulmaria* (L.) Maxim.  KM84 | Boukissi | Herb | Aerial parts | Aerial part extract | Flavonoids ([Genig and Ladnaya, 1980](#_ENREF_90)) | 46 | 0.18 | 0.003 | 1 ○, 2 ○, 3 ○, 4 ○, 5 ○, 6 ○, 7 ○, 8 ○, 9 ○, 10 ○, 11 ○, 12 ○, 13 ○, 14 ○, 15 ○, 16 ○, 17 ○, 18 ○, 19 ○, 20 ○, 21 ○, 22 ○, 23 ○, 24 ○, 25 ○, 26 ○, 27 ○, 28 ○, 29 ○, 30 ○, 31○ | Not reported |
| Rosaceae  *Fragaria vesca* L.  KM86 | Jangli  (Strawberry, Morangueiro) | Herb | Leaf | Dried Leaf decoction and fresh fruit extract. | Phenolocs ([Mudnic et al., 2009](#_ENREF_178)) | 47 | 0.19 | 0.003 | 1 ○, 2 ○, 3 ○, 4 ○, 5 ○, 6 ●, 7 ○, 8 ○, 9 ○, 10 ○, 11 ○, 12 ○, 13 ○, 14 ○, 15 ○ , 16 ○, 17 ○, 18 ○, 19 ○, 20 ○, 21 ○, 22 ○, 23 ○, 24 ○, 25 ○, 26 ○, 27 ○, 28 ○, 29 ○, 30 ○, 31○ | The plant is used in the treatment of (6) tonsillitis, disinfectant and  healing of wounds |
| Rosaceae  *Malus communis* Desf.  KM115 | Saib  (Teffah) | Tree | Aerial parts | Extract of Seed | Flavonoids, phenolic acids ([Eberhardt et al., 2000](#_ENREF_79)) | 68 | 0.27 | 0.004 | 1 ○, 2 ○, 3 ○, 4 ○, 5 ○, 6 ○, 7 ○, 8 ○, 9 ○, 10 ○, 11 ○, 12 ○, 13 ○, 14 ○, 15 ○, 16 ○, 17 ○, 18 ○, 19 ○, 20 ○, 21 ○, 22 ○, 23 ○, 24 ○, 25 ○, 26 ○, 27 ○, 28 ○, 29 ○, 30 ○, 31○ | Not reported |
| Rosaceae  *Rosa indica* L.  KM162 | Gulab  Rose | Shrub | Flower | Flower are boiled in water and add honey | Alkaloids, carbohydrates, flavonoids, triterpenoides, steroids ([Krishnaiah et al., 2009](#_ENREF_138)) | 98 | 0.39 | 0.003 | 1 ○, 2 ○, 3 ○, 4 ○, 5 ○, 6 ○, 7 ○, 8 ○, 9 ○, 10 ○, 11 ○, 12 ○, 13 ○, 14 ○, 15 ○, 16 ○, 17 ○, 18 ○, 19 ○, 20 ○, 21 ○, 22 ○, 23 ○, 24 ○, 25 ○, 26 ○, 27 ○, 28 ○, 29 ○, 30 ○, 31 ○ | Not reported |
| Rosaceae  *Rubus ellipticus* L.  KM163 | (Yellow Himalayan raspberry ) | Herb | Root | Extract of Root | Phenolics, flavonoids ([Saklani et al., 2012](#_ENREF_218)) | 99 | 0.40 | 0.002 | 1 ○, 2 ○, 3 ○, 4 ○, 5 ○, 6 ○, 7 ○, 8 ○, 9 ○, 10 ○, 11 ○, 12 ○, 13 ○, 14 ○, 15 ○, 16 ○, 17 ○, 18 ○, 19 ○, 20 ○, 21 ○, 22 ○, 23 ○, 24 ○, 25 ○, 26 ○, 27 ○, 28 ○, 29 ○, 30 ●, 31 ○ | Plant is used in treatment of (30) fodder, and fencing |
| Rosaceae  *Rubus ulmifolius* Schott  KM164 | () | Shrub | Leaf | Decoction is prepared from Leaf and taken internally, 150 cc, three times/day. | Phenolics, flavonoids, ascorbic acid, lycopene ([Dall’Acqua et al., 2008](#_ENREF_71)) | 100 | 0.40 | 0.004 | 1 ○, 2 ○, 3 ○, 4 ○, 5 ○, 6 ○, 7 ○, 8 ○, 9 ○, 10 ○, 11 ○, 12 ○, 13 ○, 14 ○, 15 ○, 16 ○, 17 ○, 18 ○, 19 ○, 20 ○, 21 ○, 22 ○, 23 ○, 24 ●, 25 ○, 26 ○, 27 ○, 28 ○, 29 ○, 30 ●, 31 ○ | Plant is used in treatment of (24) raw, snacks, (30) carminative, fodder, fruit and fencing |
| Rubiaceae  *Coffea Arabica* L.  KM56 | Cooffee  (Coffee) | Shrub | Leaf | Leaf extract is prepared by mixing several compounds to | Myo-inositol, phosphorylated form, phytic acid and Quinic acid ([Rogers et al., 1999](#_ENREF_210)) | 108 | 0.43 | 0.001 | 1 ○, 2 ○, 3 ○, 4 ○, 5 ○, 6 ○, 7 ○, 8 ○, 9 ○, 10 ○, 11 ○, 12 ○, 13 ○, 14 ○, 15 ○, 16 ○, 17 ○, 18 ○, 19 ○, 20 ○, 21 ○, 22 ○, 23 ○, 24 ○, 25 ○, 26 ○, 27 ○, 28 ○, 29 ○, 30 ○, 31○ | Not reported |
| Rutaceae  *Citrus aurantiifolia* (Christm.) Swingle  KM48 | Moosambi  (Kaghazi lemon, bartender's lime) | Shrub | Fruits, Leaf | Fruit and leaf extract | Alkaloids and cardiac gyycosides ([Ebana et al., 1991](#_ENREF_13)) | 24 | 0.10 | 0.063 | 1 ○, 2 ○, 3 ○, 4 ○, 5 ○, 6 ○, 7 ○, 8 ○, 9 ○, 10 ○, 11 ○, 12 ○, 13 ○, 14 ○, 15 ○, 16 ○, 17 ○, 18 ○, 19 ○, 20 ○, 21 ○, 22 ○, 23 ○, 24 ○, 25 ○, 26 ○, 27 ○, 28 ○, 29 ○, 30 ○, 31○ | Not reported |
| Rutaceae  *Citrus aurantium* L.  KM49 | Naranji  (Zhar limoun) | Tree | Fruits | Tea prepared from fruit peels | Phenolic compounds ([Karimi et al., 2012](#_ENREF_130)) | 108 | 0.43 | 0.003 | 1 ○, 2 ○, 3 ○, 4 ●, 5 ●, 6 ○, 7 ○, 8 ○, 9 ○, 10 ○, 11 ○, 12 ○, 13 ○, 14 ○, 15 ○, 16 ○, 17 ○, 18 ○, 19 ○, 20 ○, 21 ○, 22 ○, 23 ○, 24 ○, 25 ○, 26 ○, 27 ○, 28 ○, 29 ○, 30 ○, 31○ | Plant is used in treatment of (4) diabetes mellitus, hypertension and cardiac diseases, (5) hypertension and diabetes |
| Rutaceae  *Citrus limon* (L.) Osbeck  KM50 | Leemu  (Lemon) | Tree | Fruits | Fresh fruits are eaten | Phenolic compounds as well as vitamins, minerals, dietary fiber, essential oils and carotenoids ([González-Molina et al., 2010](#_ENREF_97)) | 25 | 0.10 | 0.233 | 1 ○, 2 ○, 3 ○, 4 ○, 5 ○, 6 ●, 7 ○, 8 ○, 9 ○, 10 ○, 11 ○, 12 ○, 13 ○, 14 ○, 15 ○, 16 ○, 17 ○, 18 ○, 19 ○, 20 ○, 21 ○, 22 ○, 23 ○, 24 ○, 25 ○, 26 ○, 27 ○, 28 ○, 29 ○, 30 ○, 31○ | Plant is used in treatment of (6) relaxant |
| Rutaceae  *Citrus sinensis* L.  KM52 | Malta  (Sweet orange) | Tree | Fruits | An infusion is prepared from one tsp dried fruit in one cup boiling water and taken daily until improvement occurs | Flavanones, phenolic acids and their ester derivatives. ([Roussos, 2011](#_ENREF_211)) | 54 | 0.22 | 0.002 | 1 ○, 2 ○, 3 ○, 4 ○, 5 ○, 6 ●, 7 ○, 8 ○, 9 ○, 10 ○, 11 ○, 12 ○, 13 ○, 14 ○, 15 ○, 16 ○, 17 ○, 18 ○, 19 ○, 20 ○, 21 ○, 22 ○, 23 ○, 24 ○, 25 ○, 26 ○, 27 ○, 28 ○, 29 ○, 30 ○, 31○ | Plant is used in treatment of (6) Hair tonic, cold and kidney |
| Rutaceae  *Zanthoxylum gilletii* (De Wild.) P.G. Waterman  KM193 | Mutatembwa | Tree | Stem | Stem is dipped in warm water for 2 hours and used this infusion early in morning without eating anything | Alkaloids, tannins, saponins, sterols ([MASINDE, 2014](#_ENREF_166)) | 64 | 0.26 | 0.004 | 1 ○, 2 ○, 3 ○, 4 ○, 5 ○, 6 ○, 7 ○, 8 ○, 9 ○, 10 ○, 11 ○, 12 ○, 13 ○, 14 ○, 15 ○, 16 ○, 17 ○, 18 ○, 19 ○, 20 ○, 21 ○, 22 ○, 23 ○, 24 ○, 25 ○, 26 ○, 27 ○, 28 ○, 29 ○, 30 ○, 31 ○ | Not reported |
| Santalaceae *Viscum album* L.  KM191 | Amar Bail  (Mistletoe, Dbake, Cekem) | Shrub | Leaf, Aerial part | Extract of whole plant. Decoction of Leaf is also effective. | Flavanones, flavanone glycosides, triterpenenes ([Samuelsson, 1958](#_ENREF_222)) | 62 | 0.25 | 0.003 | 1 ○, 2 ○, 3 ○, 4 ●, 5 ○, 6 ○,, 7 ○, 8 ○, 9 ○, 10 ●, 11 ○, 12 ○, 13 ○, 14 ○, 15 ○, 16 ○, 17 ○, 18 ○, 19 ○, 20 ○, 21 ○, 22 ○, 23 ○, 24 ○, 25 ○, 26 ○, 27 ○, 28 ●, 29 ○, 30 ○, 31 ○ | Plant is used in treatment of (4) diabetes, hypertension and cardiac diseases, (10) hypertension, (28) hypertension |
| Sapindaceae  Dodonaea viscosa (L.) Jacq  KM19 | (Grapeleaf begonia) | Shrub | Flower | Dried Flower infusion | Alkaloids, terpenoids, saponins, tannins, sugars, phenolics, flavonoids  and cardiac glycosides ([Riaz et al., 2012](#_ENREF_209)) | 27 | 0.11 | 0.042 | 1 ○, 2 ○, 3 ○, 4 ○, 5 ○, 6 ○, 7 ○, 8 ○, 9 ○, 10 ○, 11 ○, 12 ○, 13 ○, 14 ○, 15 ○, 16 ○, 17 ○, 18 ○, 19 ○, 20 ○, 21 ○, 22 ○, 23 ○, 24 ○, 25 ○, 26 ○, 27 ○, 28 ○, 29 ○, 30 ○, 31○ | Not reported |
| Sapindaceae  *Melicoccus bijugatus* Jacq.  KM124 | (Spanish lime, genip) | Tree | Leaf | Decoction of Leaf and Seed | Phenolics ([Bystrom et al., 2009](#_ENREF_59)) | 74 | 0.30 | 0.004 | 1 ○, 2 ○, 3 ○, 4 ○, 5 ○, 6 ○, 7 ○, 8 ○, 9 ○, 10 ○, 11 ○, 12 ○, 13 ○, 14 ○, 15 ○, 16 ○, 17 ○, 18 ○, 19 ○, 20 ○, 21 ○, 22 ○, 23 ○, 24 ○, 25 ○, 26 ○, 27 ○, 28 ○, 29 ○, 30 ○, 31○ | Not reported |
| Solanaceae  *Cestrum racemosum* Ruiz & Pav.  KM41 | (Sauco negro) | Shrub | Whole plant | An infusion is prepared from one tsp. of the plant material in one cup water and taken orally |  | 162 | 0.65 | 0.003 | 1 ○, 2 ○, 3 ○, 4 ○, 5 ○, 6 ●, 7 ○, 8 ○, 9 ○, 10 ○, 11 ○, 12 ○, 13 ○, 14 ○, 15 ○, 16 ○, 17 ○, 18 ○, 19 ○, 20 ○, 21 ○, 22 ○, 23 ○, 24 ○, 25 ○, 26 ○, 27 ○, 28 ○, 29 ○, 30 ○, 31○ | Plant is used in treatment of (6) tooth decay, headache,  stomach pain, fever, gastritis,  influenza and high blood |
| Solanaceae  *Datura stramonium* L.  KM74 | Dhatura  (Chdak ejmel) | Herb | Aerial parts | Dried fruits are used to make extract. | Tropane alkaloids, atropine, hyoscyamine  ([Banso and Adeyemo, 2006](#_ENREF_47)) | 39 | 0.16 | 0.003 | 1 ○, 2 ○, 3 ●, 4 ●, 5 ●, 6 ○, 7 ○, 8 ○, 9 ○, 10 ○, 11 ○, 12 ○, 13 ○, 14 ○, 15 ○, 16 ●, 17 ○, 18 ○, 19 ○, 20 ○, 21 ○, 22 ○, 23 ○, 24 ○, 25 ○, 26 ○, 27 ○, 28 ○, 29 ○, 30 ○, 31● | Plant is used in the treatment of (3) diabetes, cardiac and renal diseases, (4) diabetes mellitus, hypertension and cardiac diseases, (5) hypertension and diabetes, (16) dermatological, topical diseases, wounds, cuts, narcotic, tonic, tumor,  anticancer and stimulant, (31) earache and antipyretic. |
| Solanaceae  *Solanum nigrum* Mill.  KM171 | Makoh | Herb | Leaf | 5-10 gm. of powder of Leaf is taken with amla juice | Solasonine, solanine ([Akubugwo et al., 2008](#_ENREF_23)) | 105 | 0.42 | 0.003 | 1 ○, 2 ○, 3 ○, 4 ○, 5 ○, 6 ●, 7 ○, 8 ○, 9 ○, 10 ○, 11 ○, 12 ○, 13 ○, 14 ○, 15 ○, 16 ○, 17 ○, 18 ○, 19 ○, 20 ○, 21 ○, 22 ○, 23 ○, 24 ○, 25 ○, 26 ○, 27 ○, 28 ○, 29 ○, 30 ○, 31 ○ | The plant is used in treatment of (6) headache, after getting drunk  effects, stomach pain, hepatic  pain, hematoma, dermatitis,  fever, influenza, internal  infections, pneumonia, cold and  kidney problem |
| Tamaricaceae  *Tamarix aphylla (*L.) H.Karst.  . KM178 | Jhau  (El aadba) | Tree | Aerial part | Aqueous extract of aerial part | Triterpene ([Merfort et al., 1992](#_ENREF_37)) | 54 | 0.22 | 0.011 | 1 ○, 2 ○, 3 ○, 4 ●, 5 ●, 6 ○,, 7 ○, 8 ○, 9 ○, 10 ○, 11 ○, 12 ○, 13 ○, 14 ○, 15 ○, 16 ○, 17 ○, 18 ○, 19 ○, 20 ○, 21 ○, 22 ○, 23 ○, 24 ○, 25 ○, 26 ○, 27 ○, 28 ○, 29 ○, 30 ○, 31 ○ | Plant is used in treatment of (4) diabetes, hypertension and cardiac diseases, (5)hypertension and diabetes |
| Theaceae  *Camellia sinensis* (L.) Kuntze  KM31 | Chaye  (Casuguong, Pagcufa) | Shrub | Root, Flower | Root and Flower are boiled in water and this boiled water is used as decoction | Polyphenolic, monomeric flavonoids (flavan 3-ols or tea catechins)  and volatile compounds ([Kim et al., 2011](#_ENREF_136)) | 78 | 0.31 | 0.003 | 1 ○, 2 ○, 3 ○, 4 ○, 5 ○, 6 ○, 7 ○, 8 ○, 9 ○, 10 ○, 11 ○, 12 ○, 13 ○, 14 ○, 15 ○, 16 ○, 17 ○, 18 ○, 19 ○, 20 ○, 21 ○, 22 ○, 23 ○, 24 ○, 25 ○, 26 ○, 27 ○, 28 ○, 29 ○, 30 ○, 31○ , 32 ○. | Not reported |
| Urticaceae  *Urtica dioica* L.  KM187 | (Bicho Booti  Harrigua | Herb | Leaf, Seed | Extract of Seed | Saponins, tannins, steroids, terpenoids, flavonoids ([Gülçin et al., 2004](#_ENREF_104)) | 81 | 0.32 | 0.002 | 1 ○, 2 ○, 3 ○, 4 ○, 5 ○, 6 ○, 7 ○, 8 ○, 9 ○, 10 ○, 11 ○, 12 ○, 13 ○, 14 ○, 15 ○, 16 ○, 17 ○, 18 ○, 19 ○, 20 ○, 21 ○, 22 ○, 23 ○, 24 ○, 25 ○, 26 ○, 27 ○, 28 ○, 29 ○, 30 ○, 31 ○ | Not reported |
| Verbenaceae  *Aloysia citrodora* Palau  KM106 | Louiza | Shrub | Leaf | Decoctions of Leaf is for a week | Flavonoids, monoterpenes, sesquiterpenes, sesquiterpenoids, and fatty alcohols ([Bahramsoltani et al., 2018](#_ENREF_1)) | 60 | 0.24 | 0.004 | 1 ○, 2 ○, 3 ○ , 4 ○ , 5 ●, 6 ○, 7 ○, 8 ●, 9 ○, 10 ○, 11 ○, 12 ○, 13 ○, 14 ○, 15 ○, 16 ○, 17 ○, 18 ○, 19 ○, 20 ○, 21 ○, 22 ○, 23 ○, 24 ○, 25 ○, 26 ○, 27 ○, 28 ○, 29 ○, 30 ○, 31○ | Plant is used in treatment of (5) hypertension and diabetes, (8) hypertension |
| Verbenaceae  *Lantana camara* L.  KM111 | Panjpahle | Shrub | Leaf, Root | Decoction of Leaf and Root boiled in water is used to treat the disease for 3 days | Triterpenoids, steroids, lactones, resins, tannins and flavonoids ([Verma and Verma, 2006](#_ENREF_267)) | 65 | 0.26 | 0.003 | 1 ○, 2 ○, 3 ○, 4 ○, 5 ○, 6 ○, 7 ○, 8 ○, 9 ○, 10 ○, 11 ○, 12 ○, 13 ○, 14 ○, 15 ○, 16 ○, 17 ○, 18 ○, 19 ○, 20 ○, 21 ○, 22 ○, 23 ○, 24 ○, 25 ○, 26 ○, 27 ○, 28 ○, 29 ○, 30 ○, 31○ | Not reported |
| Vitaceae  *Cissus simsiana* Roem. & Schult.  KM46 | Hrajora  (Cipo-parreira) | Shrub | Flower, Leaf | An infusion made of 50 g Leaf are soaked in Luke warm water for 30 min, one cup per day is taken orally. | None | 23 | 0.09 | 0.017 | 1 ○, 2 ○, 3 ○, 4 ○, 5 ○, 6 ○, 7 ○, 8 ○, 9 ○, 10 ○, 11 ○, 12 ○, 13 ○, 14 ○, 15 ○, 16 ○, 17 ○, 18 ○, 19 ○, 20 ○, 21 ○, 22 ○, 23 ○, 24 ○, 25 ○, 26 ○, 27 ○, 28 ○, 29 ○, 30 ○, 31○ , 32 ○. | Not reported |
| Vitaceae  *Vitis vinifera* L.  KM192 | Angoor  (Inab) | Vine | Leaf, Fruits | Leaf and fruits extract | Phenolics, flavonoids, flavonols ([Guendez et al., 2005](#_ENREF_103)) | 63 | 0.25 | 0.003 | 1 ○, 2 ○, 3 ○, 4 ●, 5 ●, 6 ○,, 7 ○, 8 ○, 9 ○, 10 ○, 11 ○, 12 ○, 13 ○, 14 ○, 15 ○, 16 ○, 17 ○, 18 ○, 19 ○, 20 ○, 21 ○, 22 ○, 23 ○, 24 ○, 25 ○, 26 ○, 27 ○, 28 ○, 29 ○, 30 ○, 31 ○ | Plant is used in treatment of (4) diabetes, hypertension and cardiac diseases, (5) hypertension and diabetes |
| Xanthorrhoeaceae  *Aloe vera* (L.) Burm.f.  KM07 | Kanwargandal  (Aloe) | Herb | Leaf | Leaf of the aloe vera are cut; liquid exudes from the wounds. This aloin rich liquid is dried and harvested, to obtain curacao aloe. This gel is mixed with milk and taken orally | Aloin, glucomannans, salicylic acid ([Arunkumar and Muthuselvam, 2009](#_ENREF_38)) | 27 | 0.11 | 0.003 | 1 ○, 2 ○, 3 ○ , 4 ○ , 5 ●, 6 ○, 7 ○, 8 ●, 9 ○, 10 ○, 11 ○, 12 ○, 13 ○, 14 ○, 15 ○, 16 ○, 17 ○, 18 ○, 19 ○, 20 ○, 21 ○, 22 ○, 23 ○, 24 ○, 25 ○, 26 ○, 27 ○, 28 ●, 29 ○, 30 ○, 31○ | Plant is for treatment of (5) hypertension and diabetes , (8) Hypertension, (28) hypertension |
| Zingiberacae  *Zingiber officinale* Roscoe  KM195 | Adrak  (Ginger) | Herb | Seed | Seed extract | Flavonoids,tannins,polyphenols ([Ali et al., 2008](#_ENREF_26)) | 66 | 0.26 | 0.004 | 1 ○, 2 ○, 3 ○, 4 ○, 5 ○, 6 ○, 7 ○, 8 ○, 9 ○, 10 ○, 11 ○, 12 ○, 13 ○, 14 ○, 15 ○, 16 ○, 17 ○, 18 ○, 19 ○, 20 ○, 21 ○, 22 ○, 23 ○, 24 ○, 25 ○, 26 ○, 27 ○, 28 ●, 29 ○, 30 ○, 31 ○ | Plant is used in treatment of (28) hypertension |
| Zygophyllaceae  *Tribulus terrestris* L. KM185 | Gokhru  Bindii | Herb | Seed | Decoction of the Seed are taken for 7 days | Alkaloids, carbohydrates, cardiac glycosides, tannins, saponins ([Usman et al., 2007](#_ENREF_264)) | 113 | 0.45 | 0.002 | 1 ○, 2 ○, 3 ○, 4 ○, 5 ○, 6 ○, 7 ○, 8 ○, 9 ○, 10 ○, 11 ○, 12 ○, 13 ○, 14 ○, 15 ○, 16 ○, 17 ○, 18 ○, 19 ○, 20 ○, 21 ○, 22 ○, 23 ○, 24 ○, 25 ○, 26 ○, 27 ○, 28 ○, 29 ○, 30 ●, 31 ○ | Plant is used in treatment of (30) urinary disorder |
| Zygophyllaceae  *Zygophyllum fabago* L. KM197 | Akeel Kohistani  Aggaya | Herb | Leaf | Extract of Leaf | Disulphated Triterpenoids ([Khan et al., 2010](#_ENREF_135)) | 27 | 0.11 | 0.001 | 1 ○, 2 ○, 3 ○, 4 ○, 5 ○, 6 ○, 7 ○, 8 ○, 9 ○, 10 ○, 11 ○, 12 ○, 13 ○, 14 ○, 15 ○, 16 ○, 17 ○, 18 ○, 19 ○, 20 ○, 21 ○, 22 ○, 23 ○, 24 ○, 25 ○, 26 ○, 27 ○, 28 ○, 29 ○, 30 ○, 31 ○ | Not reported |
